# Transcriptomic response to different heme sources in *Trypanosoma cruzi* epimastigotes

**DOI:** 10.15698/mic2026.01.865

**Published:** 2026-01-23

**Authors:** Evelyn Tevere, María G. Mediavilla, Cecilia B. Di Capua, Marcelo L. Merli, Carlos Robello, Luisa Berná, Julia A. Cricco

**Affiliations:** 1 Instituto de Biología Molecular y Celular de Rosario (IBR), Consejo Nacional de Investigaciones Científicas y Técnicas (CONICET)— Universidad Nacional de Rosario (UNR), Rosario, Argentina; 2 Laboratorio de Interacciones Hospedero-patógeno — UBM, Institut Pasteur de Montevideo, Montevideo, Uruguay; 3 Departamento de Bioquímica, Facultad de Medicina, Universidad de la República, Montevideo, Uruguay; 4 Laboratory of Apicomplexan Biology, Institut Pasteur de Montevideo, Uruguay; 5 Bioinformatic Unit, Institut Pasteur de Montevideo, Uruguay; 6 Laboratorio de Genómica Evolutiva, Facultad de Ciencias, Universidad de la República, Uruguay

**Keywords:** Chagas disease, *Trypanosoma cruzi*, heme, hemoglobin, transcriptome

## Abstract

Heme is an essential molecule for most organisms, yet some parasites, like *Trypanosoma cruzi*, the causative agent of Chagas disease, cannot synthesize it. These parasites must acquire heme from their hosts, making this process critical for their survival. In the midgut of the insect vector, *T. cruzi* epimastigotes are exposed to both hemoglobin (Hb) and free heme resulting from its degradation. Despite the importance of this nutrient, how different heme sources influence parasite gene expression remains poorly understood.

Here, we showed that heme restitution either as hemin or Hb to heme-starved parasites induces an early and distinct transcriptional response in *T. cruzi* epimastigotes. Using RNA sequencing at 4- and 24-hours post-supplementation, we identified gene subsets commonly or uniquely regulated by each heme source, including genes putatively linked to heme acquisition and metabolism. The study includes the first focused characterization of CRAL/TRIO domain-containing protein (TcCRAL/TRIO), a novel heme-responsive hemoprotein. Our results provide a more detailed picture of *T. cruzi* biology and highlights heme acquisition as a promising point of vulnerability to control parasite proliferation.

## INTRODUCTION

*Trypanosoma cruzi* is the causative agent of Chagas disease, a human neglected disease. This parasite presents a digenetic life cycle comprising two hosts (a mammalian host and a hematophagous triatomine vector) and, at least, four well defined developmental stages.

Trypanosomatids are aerobic organisms that rely on hemoproteins for key metabolic processes, including ergosterol biosynthesis, fatty acid desaturation, and mitochondrial respiration 1. However, *T. cruzi*, as well as other trypanosomatids, cannot synthesize heme and must acquire it from its hosts 2, 3. This parasite can incorporate the cofactor during its replicative life-cycle stages, epimastigotes and amastigotes 4. In the triatomine midgut, hemoglobin (Hb, a heme-bound tetrameric protein), serves as a significant heme source, while free heme (released from protein degradation) is directly bioavailable for epimastigotes 5. However, free heme also poses a significant threat due to its capacity to catalyze the formation of reactive oxygen species (ROS), which can damage cellular components 6. To counteract the toxic effects of ROS and enhance its survival, *T. cruzi* has evolved several defense mechanisms, including the trypanothione system—a functional analog of the glutathione system in most eukaryotes—along with robust DNA repair pathways and unique DNA polymerases 7.

Previously, we studied the participation of *Tc*HRG (former *Tc*HTE) in epimastigote heme uptake both as free heme (added as hemin) 4, 8 and as Hb 9. Our findings revealed that the parasite modulates heme uptake in response to intracellular heme levels to balance the supply of this essential cofactor for hemoproteins while mitigating its potential toxicity. Specifically, heme starvation triggers an increase in *Tc*HRG at mRNA and protein levels which are subsequently downregulated within the first 24 hours of hemin or Hb restoration 8, 9. However, the precise mechanisms underlying the regulation of *Tc*HRG gene expression, the intracellular trafficking of heme, and its utilization as a cofactor or as an iron source remain to be elucidated. Our results also support that *Tc*HRG is involved in the control of heme homeostasis in the amastigotes stage 8. Since the host-cell cytoplasm is a compartment where heme is tightly regulated and mostly bound to proteins (“hardly coordinated” or non-exchangeable, and “labile heme” or exchangeable heme) 10, 11, amastigotes might acquire part of the required heme through endocytosis of hemoproteins via the cytostome–cytopharynx complex, which was demonstrated that is functional in this stage 12 and via a *Tc*HRG mediated process.

In this study, we used transcriptome analysis as a tool to investigate the molecular response of the epimastigote life-cycle stage to heme stress, analyzing the replenishment of the heme source, provided as either hemin or Hb, in cultures previously starved of this cofactor.

The analysis of the differentially expressed genes (DEGs) over time indicates that *T. cruzi* epimastigote exhibits both shared and distinct transcriptional responses to different sources of heme. A core set of genes commonly modulated by hemin and Hb suggests a conserved heme-responsive program, involving redox balance and metabolic adaptations. Hemin triggers extensive remodeling of gene expression related to structural components, glycolysis, and signaling pathways. In contrast, Hb supplementation elicits a more limited but distinct transcriptional shift, marked by the induction of proteolytic enzymes and modulation of signaling components.

Additionally, the analysis of the heme-supplemented samples (hemin or Hb) *vs.* heme-deprived samples (no heme addition after starvation) revealed a small but consistent set of DEGs, most of them downregulated. Notably, a CRAL/TRIO domain-containing protein (named here *Tc*CRAL/TRIO), consistently repressed by heme, emerged as a key candidate for further study. Biochemical assays confirmed *Tc*CRAL/TRIO as a hemoprotein, suggesting a role in heme homeostasis in *T. cruzi*.

Therefore, the aim of this study is to use RNA-sequencing to characterize the transcriptomic response of *T. cruzi* epimastigotes to different heme sources. These results will not only advance our understanding of basic heme homeostasis in this life stage but will also serve as a crucial foundation for exploring key vulnerabilities in human-infective forms, including intracellular amastigotes.

## RESULTS AND DISCUSSION

### *TcHRG* expression presents a fine-tuned response to heme

**Figure 1:**
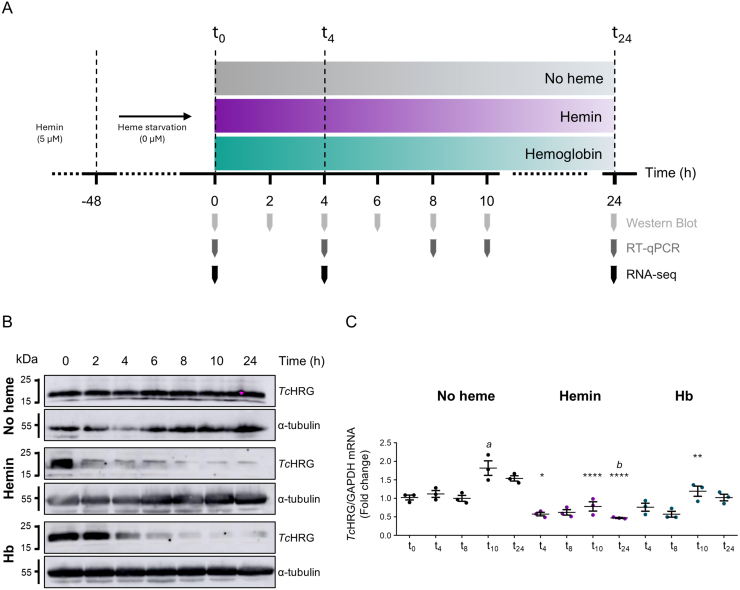
**(A)** Experimental design scheme. Epimastigotes cultured in LIT+10% FBS + 5 μ M hemin were subjected to heme starvation for 48 h. Parasites were then transferred to fresh media supplemented with 5 μ M hemin, 1.25 μ M hemoglobin (Hb), or without heme. The time points for sample collection used in *Tc*HRG analysis by western blot and RT-qPCR as well as for the samples included in the RNA-seq analysis are indicated. **(B)** Western blot analysis of *Tc*HRG protein levels and α -tubulin in the three conditions analyzed (no heme, 5 μ M hemin, and 1.25 μ M Hb) at different time points. **(C)** RT-qPCR analysis of *Tc*HRG mRNA levels in the three conditions: no heme (black dots), 5 μ M hemin (purple dots), and 1.25 μ M Hb (blue dots), at different time points post-treatment, using GAPDH as the reference gene. Data are presented as the mean ± SD of three biological replicas. Statistical significance was determined by one-way ANOVA followed by Tukey’s multiple comparisons test (^⁎⁎⁎⁎^: p < 0.0001; ^⁎⁎⁎^: p < 0.001; ^⁎⁎^: p < 0.01; ^⁎^: p < 0.05, significantly different from no heme condition at the same time of incubation. *a*: p < 0.001; *b*: p < 0.05, significantly different against no heme t ${}_{0}$ sample).

To gain insight and identify genes and pathways involved in heme homeostasis of *T. cruzi* epimastigotes, we investigated its transcriptional response to heme by RNA-sequencing (RNA-seq) using free heme (added as hemin) and Hb as heme sources. We chose *TcHRG* as a model heme responsive gene 8, 9 and performed a heme-response time course experiment in epimastigotes. Briefly, epimastigotes routinely cultured in the presence of 5 μ M hemin were subjected to heme starvation (LIT+10% FBS without the addition of a heme source) for 48 h. Then, parasites were transferred to fresh media supplemented with 5 μ M hemin, 1.25 μ M Hb (equivalent to 5 μ M heme as Hb) or without heme (heme-deprived samples, identified as “No heme” in figures). The starvation step ensured uniform starting conditions for assessing the effect of heme restitution for both sources. Samples were collected immediately before repletion (t ${}_{0}$ ) and at different time intervals over 24 hours (Figure 1**A**).

Western blotting using anti-*Tc*HRG polyclonal antibodies 8 showed that *Tc*HRG protein signal remained stable in heme-deprived parasites but decreased markedly 6–8 hours after supplementation with either hemin or Hb, being this effect more pronounced in hemin-treated samples (Figure 1**B**).

*TcHRG* transcript levels were also analyzed by RT-qPCR (Figure 1**C**). In heme-deprived epimastigotes, they remained stable up to 8 hours, followed by an increase at 10 and 24 hours after medium renewal. Upon heme reintroduction, transcript levels dropped approximately 50 % and 25 % after 4 hours of hemin and Hb refeeding, respectively. These changes in mRNA levels mirrored the protein expression patterns, with a more marked response to hemin than to Hb, consistent with our previous observations 9.

Based on these observations, we conducted the RNA-seq assay using the experimental scheme shown in Figure 1**A**. Samples were collected at three time points: after heme starvation (t ${}_{0}$ ), 4 hours (t ${}_{4}$ ), and 24 hours post heme refeeding (t ${}_{24}$ ). Heme-deprived cultures were included to account for experimental noise arising only from medium replacement and the dilution steps used to keep the parasites in logarithmic growth.

For this study we selected the *T. cruzi* Dm28c strain, classified as DTU TcI, because this lineage is the most prevalent in South America and is highly relevant for human infections 13. Also, the Dm28c strain is widely used as a reference model, with a well-annotated and high-quality genome that makes it suitable for transcriptomic analyses 14. Nonetheless, responses may differ across *T. cruzi* lineages, and therefore our findings should be validated in additional genetic backgrounds.

### Heme restitution induces rapid and mild changes in epimastigotes’ transcriptome

**Figure 2:**
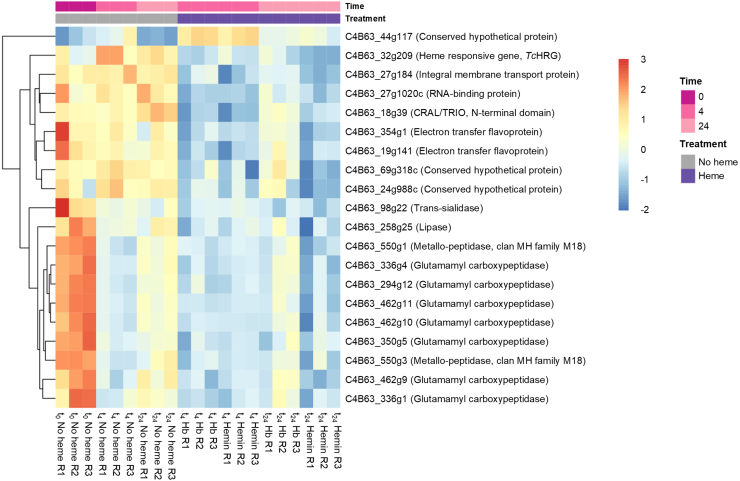
Heatmap illustrating the expression patterns of differentially expressed genes in heme-supplemented (hemin or hemoglobin, Hb) and heme-deprived (no heme) conditions, irrespective of time. Gene IDs and annotations listed on the right correspond to the *Trypanosoma cruzi* Dm28c 2018 genome sequence and annotation (*TriTrypDB*). Sample nomenclature is as follows (example): “t ${}_{0}$ No heme R1” refers to a No heme sample from experimental replica 1 at time 0 h.

A global comparison between heme-supplemented (hemin or Hb) and heme-deprived conditions, irrespective of time, identified 51 DEGs (Table S1). In this initial analysis, *TcHRG* showed a log ${}_{2}$ (fold change) (logFC) of −0.48, consistent with our previous results 8, 9. Based on this and considering the moderate variations detected in the overall transcriptional response, we established a logFC threshold of |0.40| as a *bona fide* cutoff for subsequent transcriptomic analyses. Applying this threshold and a statistical significance criterion of adjusted p-value (padj) < 0.05, we identified 20 DEGs (Figure 2). Although the overall number of DEGs is relatively small, the affected genes suggest potential transcriptional adjustments related to protein processing, metabolism, and electron transport. The specific contributions of these pathways will be explored in detail in the following sections.

**Figure 3:**
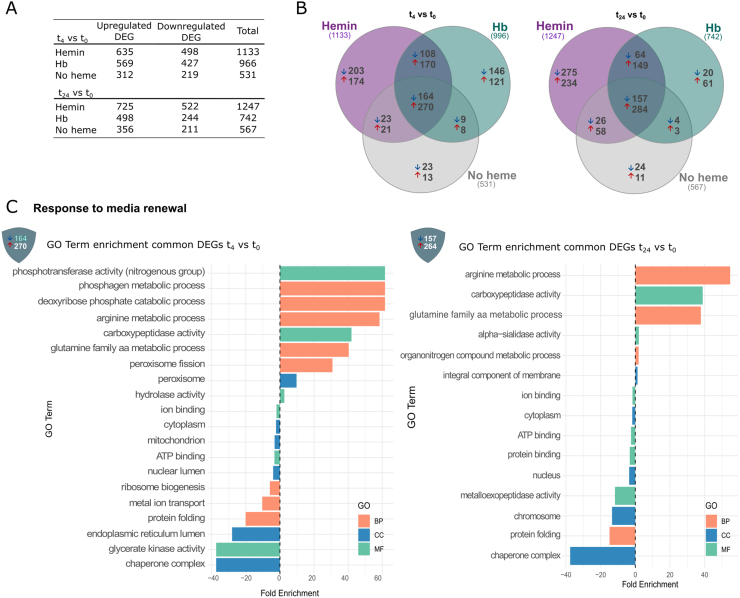
**(A)** Differentially expressed genes (DEGs) at t ${}_{4}$ and t ${}_{24}$ after media renewal compared to baseline levels (t ${}_{0}$ ) in each of the three culture conditions: no heme, hemin, and hemoglobin (Hb). **(B)** Venn diagrams showing DEGs among the no heme, hemin, and Hb conditions at t ${}_{4}$ vs*.* t ${}_{0}$ and t ${}_{24}$ vs*.* t ${}_{0}$ . **(C)** Gene Ontology (GO) enrichment analysis of genes shared among the three conditions at t ${}_{4}$ vs. t ${}_{0}$ and t ${}_{24}$ vs*.* t ${}_{0}$ . BP: Biological Process. CC: Cellular Component. MF: Molecular Function.

To further dissect the transcriptional response to heme over time, we analyzed DEGs at t ${}_{4}$ and t ${}_{24}$ against baseline levels (t ${}_{0}$ ) in heme-deprived, hemin, and Hb conditions (Figure 3**A** and Table S2).

At first, a subset of DEGs shared across all conditions, comprising 434 genes (t ${}_{4}$ vs. t ${}_{0}$ ) and 441 genes (t ${}_{24}$ vs. t ${}_{0}$ ) (Figure 3**B**, Table S2) were excluded from further analysis. We considered they represent a general response to media renewal and parasite dilution rather than a specific effect of variations in heme availability (Figure 3**C**).

As mentioned earlier, very few DEGs exhibited a logFC greater than |1| (doubling or halving their expressions) (Table S2) and more than 75% showed moderate expression changes, with logFC values between |0.4| and |0.7| (Figure S1). Still, transcriptional changes were already detectable at t ${}_{4}$ , indicating an early adjustment to heme restoration.

#### Genes involved in the core transcriptional response to heme

We further analyze if pathways involved in heme metabolism and oxidative stress response in *T. cruzi* are affected by both sources of heme. In this case, the overlapping DEGs to both heme sources-hemin and Hb (hemin ∩ Hb)-will arise as the core regulation by the cofactor. This group included 108 downregulated and 170 upregulated genes in the t ${}_{4}$ vs*.* t ${}_{0}$ dataset, and 64 downregulated and 149 upregulated genes in the t ${}_{24}$ vs*.* t ${}_{0}$ dataset (Table 1, Table S3).

**Figure 4:**
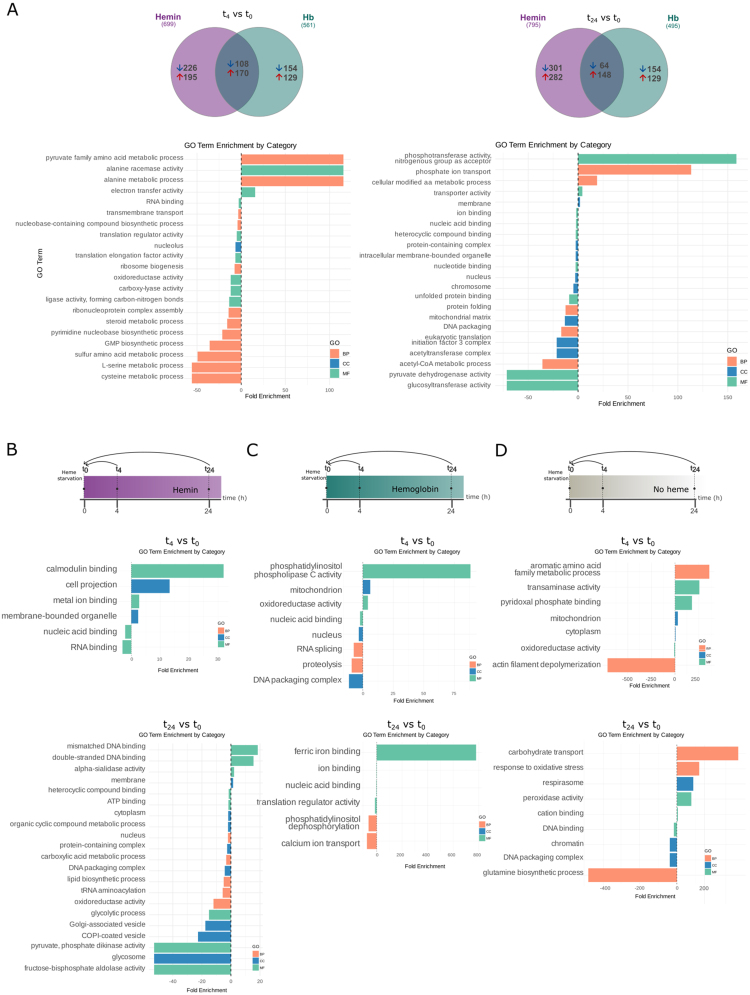
Differentially expressed genes (DEGs) in no heme, hemin, hemoglobin (Hb) conditions, and merged heme (hemin and Hb) conditions. **(A)** Common genes detected simultaneously in hemin- and Hb-supplemented conditions, along with Gene Ontology (GO) analysis of the response to heme. **(B)** GO analysis of DEGs in the hemin condition at t ${}_{4}$ *vs.* t ${}_{0}$ and t ${}_{24}$ vs*.* t ${}_{0}$ . **(C)** GO analysis of DEGs in the Hb condition at t ${}_{4}$ vs*.* t ${}_{0}$ and t ${}_{24}$ vs*.* t ${}_{0}$ . **(D)** GO analysis of DEGs in the no heme condition at t ${}_{4}$ vs*.* t ${}_{0}$ and t ${}_{24}$ vs*.* t ${}_{0}$ . BP: Biological Process. CC: Cellular Component. MF: Molecular Function.

Gene Ontology (GO) analysis (Figure 4**A**, Table S3) of upregulated genes at t ${}_{4}$ vs. t ${}_{0}$ dataset showed enrichment in GO terms including serine and cysteine metabolism, transmembrane transport, ribosome biogenesis, pyrimidine biosynthesis, steroid metabolism, oxidoreductase activity, and nucleolus components, among others. In the t ${}_{24}$ vs. t ${}_{0}$ dataset, enriched processes included terms such as protein folding, acetyl-CoA metabolism, and DNA packaging. Many of the identified genes were associated with cellular components from the mitochondrial matrix and nucleus. Additional upregulated genes were involved in binding to small molecules and unfolded protein binding, as well as displaying hexosyl transferase and pyruvate dehydrogenase activities. Notably, a gene encoding the glycolytic enzyme enolase was upregulated at t ${}_{24}$ . Terms involving translation regulation were also significantly enriched at both time points.

**Table 1:** Differentially expressed genes uniquely modulated under the no heme, hemin, or hemoglobin (Hb) conditions. These genes were identified in the t ${}_{4}$ vs. t ${}_{0}$ and t ${}_{24}$ vs*.* t ${}_{0}$ comparisons, excluding those whose expression changes were attributed to media renewal (*i.e.*, genes common to all conditions).

	**Upregulated DEGs**	**Downregulated DEGs**	**Total**
t ${}_{4}$ vs*.* t ${}_{0}$			

No heme	13	23	36

Hemin	174	203	377

Hb	121	146	267

t ${}_{24}$ vs*.* t ${}_{0}$			

No heme	11	24	35

Hemin	234	275	509

Hb	61	20	81

A thorough analysis of the upregulated hemin ∩ Hb DEGs identified several genes involved in trypanothione biosynthesis and regeneration and ROS detoxification 47. These include a diphthine synthase gene (t ${}_{4}$ ), 19 cystathionine beta synthase (CBS) genes (t ${}_{4}$ ), a trypanothione reductase gene (t ${}_{4}$ ), a spermidine synthase gene (t ${}_{4}$ and t ${}_{24}$ ), a pyridoxal kinase gene (t ${}_{24}$ ), and a putrescine-cadaverine transporter gene (t ${}_{24}$ ). Also, we found upregulated two peroxidoxin genes (t ${}_{24}$ ), which are likely involved in detoxifying ROS. Additionally, two genes encoding for enzymes that produce reducing equivalents as NADPH also appeared upregulated: glucose-6-phosphate dehydrogenase (G6PD) gene (t ${}_{4}$ and t ${}_{24}$ ) and malic enzyme gene (t ${}_{4}$ ).

Regarding downregulated genes, GO analysis at t ${}_{4}$ vs. t ${}_{0}$ revealed enrichment in molecular function terms such as alanine metabolic process and electron transfer activities. For example, we observed the downregulation of genes encoding subunits VI and VIII of cytochrome c oxidase and two electron transfer flavoproteins.This observation aligns with previous reports that heme inhibits cytochrome c oxidase activity in *T. cruzi*, leading to reduced oxygen consumption and increased mitochondrial ROS production 6. Another possible interpretation is that heme starvation promotes the synthesis of key apo-hemoproteins to maximize heme capture and incorporation when this cofactor becomes available. Upon heme repletion, expression of these genes may be downregulated since adequate protein reserves have already been synthesized.

In the t ${}_{24}$ vs. t ${}_{0}$ comparison, the enriched terms include phosphotransferase activity, transporter activity, and membrane components.

It is also worth mentioning the downregulation of genes involved in arginine metabolism and fatty acid metabolism. We identified two (at t ${}_{4}$ ) and six (at t ${}_{24}$ ) copies of the arginine permease gene and three copies of the arginine kinase gene (at t ${}_{24}$ ). *T. cruzi* epimastigotes convert L-arginine into L-phosphoarginine via arginine kinase, generating phosphagens that serve as energy reservoirs under nutrient-limiting conditions 15, 16. The downregulation of these genes after heme refeeding might reflect a shift in energy, as need for this buffering system diminished once normal metabolism is restored.

Also, analysis at t ${}_{4}$ revealed a downregulation of genes encoding for oleate desaturase and associated proteins (cytochrome b5-dependent oleate desaturase, cytochrome b5, and cytochrome b5 domain-containing protein 1) in response to heme supplementation. A homolog of oleate desaturase has been characterized in *T. brucei*, where it contributes to the production of unsaturated fatty acids, modulating membrane fluidity 17. It is plausible that these genes were upregulated during heme starvation to initiate metacyclogenesis and enhance membrane fluidity 18. In fact, there is evidence that heme plays a central role as a metabolic regulator of *T. cruzi* metacyclogenesis 19. Upon heme refeeding, the return to the epimastigote state would lessen the requirement for such adaptations, consistent with the observed transcriptional downregulation.

Altogether, these findings suggest that heme availability not only modulates redox balance and energy metabolism but also contributes to reverse starvation-induced differentiation processes, likely through signaling pathways yet to be clarified.

Complementary to this core transcriptional signature that balances heme utilization and detoxification, we also found heme source-specific metabolic adaptations across the three culture conditions examined.

#### Hemin induces changes in the expression of specific genes

To assess the specific transcriptional response to hemin, we analyzed DEGs uniquely modulated under this condition, excluding those shared with the Hb treatment or the heme-deprived samples. In this group, we identified 203 downregulated and 174 upregulated genes in the t ${}_{4}$ vs. t ${}_{0}$ dataset, while in the t ${}_{24}$ vs. t ${}_{0}$ comparison, 275 genes were downregulated and 234 were upregulated (Table 1, Table S4 and Figure S2). In this analysis, *TcHRG* displayed a logFC of −0.45 (for t ${}_{24}$ *vs.* t ${}_{4}$ ), consistent with RT-qPCR results (Figure 1**C**).

GO enrichment analysis (Figure 4**B** and Table S4) of upregulated genes were enriched for the nucleic acid binding function at t ${}_{4}$ , while at t ${}_{24}$ , genes related to lipid biosynthesis, amino acid activation, carboxylic acids, organic cyclic compound metabolism, and glycolysis were significantly overrepresented. Within the glycolysis term, we found genes encoding two fructose-bisphosphate aldolases, two 3-phosphate dehydrogenases, and one phosphoglycerate kinase, consistent with the pattern observed in the shared hemin ∩ Hb group. These findings support the model proposed by Paes *et al.*, in which heme triggers a metabolic shift from mitochondrial respiration to aerobic fermentation in *T. cruzi* 20.

Regarding downregulated genes, analysis highlighted a reduction in the expression of genes associated with the flagellum (t ${}_{4}$ ) and membrane (t ${}_{24}$ ). For example, 18 and 31 trans-sialidase (TS) genes showed decreased expression at t ${}_{4}$ and t ${}_{24}$ , respectively. Additionally, 19 mucin-associated surface proteins (MASP) genes were downregulated at t ${}_{24}$ . Although certain members of these gene families are also active in epimastigotes 21, most of TS and MASP genes are predominantly expressed in trypomastigotes. As previously proposed, heme deprivation might promote metacyclogenesis, potentially explaining the observed TS and MASP upregulation. Again, hemin refeeding may have reversed this process, promoting a return to the epimastigote stage and suppressing the expression of these surface proteins.

The GO terms calmodulin binding and metal ion binding (t ${}_{4}$ ) and mismatched DNA binding (t ${}_{24}$ ) revealed downregulation of 11 genes encoding paraflagellar rod proteins (PRP), and 18 flagellar calcium-binding proteins (FCaBP), among others 22. In trypanosomatids, the flagellum is not only a motility organelle but also plays a key role in sensing the extracellular environment (23 and references therein). FCaBPs are calcium sensors that interact with other flagellar proteins in a Ca ${}^{2+}$ -dependent manner 24. Thus, downregulation of these components may reflect a metabolic adaptation where active environmental sensing becomes less essential following hemin-mediated alleviation of nutritional stress.

Apart from the genes identified in the hemin ∩ Hb dataset, two additional genes encoding proteins related to mitochondrial electron transport chain were downregulated in this condition: NADH-ubiquinone oxidoreductase mitochondrial (t ${}_{4}$ ) and cytochrome c oxidase assembly protein (t ${}_{24}$ ). Finally, we also found a subset of DEGs encoding proteins that may participate in signaling pathways in response to hemin (highlighted in yellow in Table S4).

In summary, the hemin-specific response was marked by a coordinated remodeling of metabolic and cell-surface functions: upregulation of glycolytic and other metabolic genes, together with downregulation of mitochondrial, membrane-associated, and flagellar components. These trends reinforce the metabolic and developmental shifts also observed in the hemin ∩ Hb dataset.

#### Hemoglobin modulates the expression of a subset of genes

To determine the specific transcriptional response to Hb, we excluded those DEGs shared with the hemin treatment or the heme-deprived samples.

The analysis of DEGs in Hb-supplemented parasites revealed 146 downregulated and 121 upregulated genes in the t ${}_{4}$ vs. t ${}_{0}$ dataset, whereas in the t ${}_{24}$ vs*.* t ${}_{0}$ comparison, 20 genes were downregulated and 61 were upregulated (Table 1, Table S4 and Figure S2).

GO analysis of upregulated genes (Figure 4**C** and Table S4) highlighted proteolysis at t ${}_{4}$ vs. t ${}_{0}$ , with 33 cruzipain genes rapidly induced. This response may indicate an immediate need for proteolytic Hb processing to secure heme—a mechanism not triggered by hemin alone—pointing to a specific response to the protein-bound form. In line with this interpretation, cruzipain is largely secreted or associated with the epimastigote surface 25, 26. These observations are consistent with our previously proposed model for Hb-derived heme uptake 9, in which extracellular Hb digestion releases heme for subsequent internalization.

In addition, genes associated with nuclear components and RNA splicing were also upregulated at t ${}_{4}$ . At t ${}_{24}$ vs. t ${}_{0}$ , molecular functions such as phosphatidylinositol dephosphorylation, translation regulation, calcium ion transport, and ion binding were significantly enriched. Also, the nucleic acid binding category was upregulated at both time points.

Regarding downregulated genes, GO analysis identified mitochondrion components and oxidoreductase and phospholipase C activities as the most affected categories in the t ${}_{4}$ vs. t ${}_{0}$ dataset. Additionally, in the t ${}_{24}$ vs. t ${}_{0}$ comparison, the biological process iron ion binding, which includes a putative frataxin gene, was significantly downregulated.

Aside from the genes found in the hemin ∩ Hb dataset, at t ${}_{4}$ we identified additional downregulated genes related to fatty acid metabolism and the mitochondrial electron transport chain in Hb supplemented parasites. These include genes encoding fatty acyl CoA synthetase and delta-4 fatty acid desaturase, as well as cytochrome c, ubiquinone biosynthesis protein COQ7 homolog, and various subunits of Complex I (subunits NB6M, NI8M, and two copies of NADH-ubiquinone oxidoreductase complex I subunit). A more detailed analysis of these DEGs revealed a group of genes involved in cellular signaling (highlighted in yellow in Table S4) that might play a role in the specific cellular response to Hb.

Overall, Hb supplementation elicited a distinct transcriptional signature, most notably characterized by the rapid induction of a large subset of cruzipain genes—highlighting a specific requirement for extracellular Hb processing to obtain heme. Beyond this major response, several additional DEGs related to mitochondrial metabolism paralleled patterns observed in the hemin ∩ Hb dataset, indicating shared metabolic adjustments to heme acquisition.

### Heme deprivation results in differential gene expression

To identify transcriptional changes associated with heme starvation, we focused on genes differentially expressed exclusively in heme-deprived samples, excluding those shared with either hemin and Hb conditions (Figures 3**B**, 4**B** and 4**C**). The analysis of this dataset revealed a small number of starvation-specific DEGs, including 23 downregulated and 13 upregulated in the t ${}_{4}$ vs. t ${}_{0}$ dataset and 24 downregulated and 11 upregulated in the t ${}_{24}$ vs. t ${}_{0}$ analysis (Table 1, Table S4).

GO enrichment analysis (Figure 4**D**, Table S4) of upregulated genes showed enrichment for terms associated with actin depolymerization and oxidoreductase activity at t ${}_{4}$ . At t ${}_{24}$ , the upregulated genes were related to nucleic acid binding, glutamine biosynthesis, and nucleosome components.

On the other hand, analysis of downregulated genes at t ${}_{4}$ indicated a significant decrease in transaminase activity, comprising 15 tyrosine aminotransferase (TAT) genes 27. In *Leishmania donovani*, deletion of a TAT ortholog disrupts redox homeostasis by increasing ROS and reducing tocopherol levels 28. This evidence suggests that in trypanosomatids TATs are implicated in redox metabolism beyond their canonical role in amino acid metabolism. Consistent with redox modulation, two ascorbate peroxidase genes (essential for oxidative stress defense, 29) were downregulated at t ${}_{24}$ . These coordinated transcriptional changes further demonstrate the capacity of *T. cruzi* to dynamically regulate antioxidant defenses in response to heme availability.

In addition, genes encoding proteins associated with mitochondrion and cytoplasm were downregulated at t ${}_{4}$ . By t ${}_{24}$ , the transcriptional response shifted, with downregulated genes including those encoding proteins of the respiratory chain complex, factors involved in oxidative stress response and carbohydrate transport, as well as proteins with cation-binding properties.

In summary, heme starvation triggered a modest but coherent transcriptional response primarily affecting redox-associated pathways. The reduced expression of tyrosine aminotransferases and ascorbate peroxidases points toward a diminished antioxidant capacity under prolonged heme starvation, which aligns with the compensatory upregulation of trypanothione biosynthetic genes observed in the shared hemin ∩ Hb dataset. Together, these findings support the notion that *T. cruzi* dynamically adjusts thiol- and redox-related metabolism in response to fluctuations in heme availability.

### Heme triggers specific expression changes in a small set of genes

**Figure 5:**
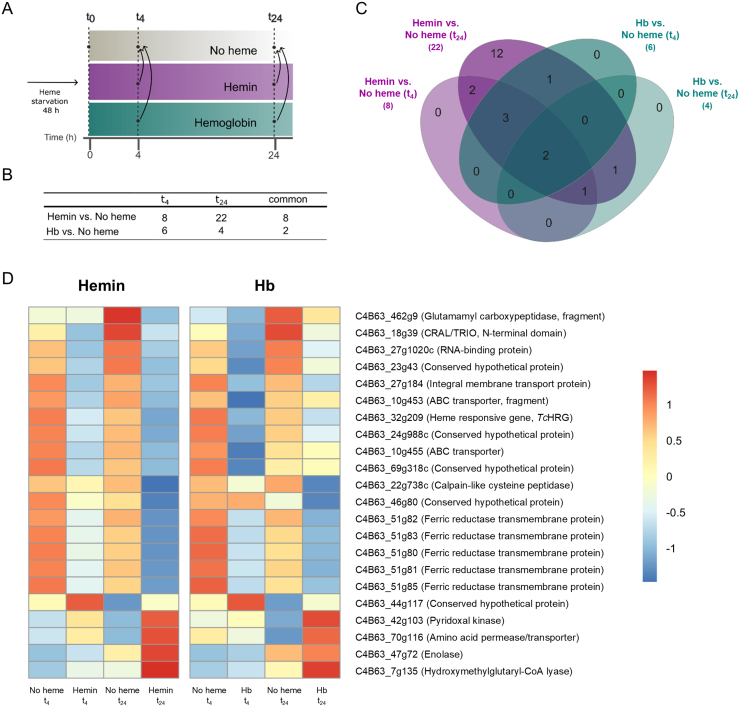
**(A)** Schematic representation of the experimental design, highlighting the comparisons between hemin- and hemoglobin (Hb)-supplemented conditions versus the no heme condition at t ${}_{4}$ and t ${}_{24}$ . **(B)** Table summarizing the number of differentially expressed genes identified (DEGs) in each comparison. **(C)** Venn diagram showing shared DEGs in each condition. **(D)** Heatmap showing the expression profiles of 22 genes differentially expressed in both hemin *vs.* no heme and Hb vs. no heme conditions at t ${}_{4}$ and t ${}_{24}$ . Gene IDs and functional annotations, shown on the right, correspond to the *T. cruzi* Dm28c 2018 genome (*TriTrypDB*).

To further explore the specific effects of heme supplementation, we performed a third analysis comparing gene expression in hemin- or Hb-supplemented cultures against the heme-deprived samples at each corresponding time point (t ${}_{4}$ and t ${}_{24}$ ) (Figure 5**A**). This analysis revealed a small but consistent set of DEGs.

In hemin-supplemented parasites, only eight downregulated genes were detected at t ${}_{4}$ . At t ${}_{24}$ , 22 genes were differentially expressed, including 17 downregulated (eight of them were already identified at t ${}_{4}$ ) and five upregulated genes (Figure 5**B** and Table S6).

For Hb-supplemented epimastigotes, a total of six downregulated genes were detected at t ${}_{4}$ , while at t ${}_{24}$ , three downregulated and one upregulated genes were identified. Only two downregulated genes were shared between t ${}_{4}$ and t ${}_{24}$ (Figure 5**C**, Table S6) and repressed by hemin. In fact, all DEGs found in Hb-supplemented samples were also included within the 22 DEGs identified in hemin-supplemented cells at t ${}_{24}$ (Figure 5**B**). In this analysis, *TcHRG* presented a logFC higher than |0.40| for hemin (t ${}_{24}$ ) and Hb (t ${}_{4}$ ).

The two downregulated genes shared between both heme sources at both time points correspond to a CRAL/TRIO domain-containing protein (C4B63_18g39) and a ferric reductase (C4B63_51g81), one of the five *TcFR* genes identified in this analysis. Additionally, an RNA-binding protein (C4B63_27g1020c) was consistently downregulated (logFC ∼−1 ) across all conditions except Hb at t ${}_{24}$ , while an integral membrane transporter (C4B63_27g184) was downregulated exclusively in hemin-supplemented samples. Moreover, a conserved hypothetical protein (C4B63_44g117) was found upregulated at t ${}_{24}$ in both hemin- and Hb-supplemented parasites (Figure 5**D**).

The five ferric reductase genes (*TcFR*), highly similar (>95% amino acid sequence identity) and bearing conserved heme-binding histidines 30, 31, were downregulated at t ${}_{24}$ in hemin-supplemented parasites. *Tc*FR is membrane-associated, reduces Fe ${}^{3+}$ to Fe ${}^{2+}$ , and is induced under iron limitation in the absence of heme 30. Notably, a ferric reductase related protein 1 (Frp1) from *Candida albicans* is required for heme uptake and relocates to the plasma membrane in response to heme 32. Thus, *Tc*FR repression here aligns with its proposed role and might reduce the heme Fe ${}^{3+}$ prior to internalization.

The RNA-binding protein encoded by C4B63_27g1020c shares 68% amino acid sequence identity with RBP5 from *T. brucei*, reported to be upregulated under iron-starvation conditions in the bloodstream form of this parasite 33. This similarity suggests the protein may have a conserved role in regulating stress-responsive genes under iron or heme deprivation.

Summing up, ten downregulated genes with unclear roles in heme metabolism were detected. Two of them, already mentioned above, were C4B63_27g1020c, that encodes an RNA-binding protein, and C4B63_18g39, that encodes a protein that contains a CRAL/TRIO domain (Interpro: IPR001251), which is commonly found in members of the SEC14 family 34. Additionally, four of these genes may be involved in metabolite transport: two ABC transporters (C4B63_10g454 and C4B63_10g455), an integral membrane transport protein (C4B63_27g184), and a conserved hypothetical DEG (C4B63_23g43) containing an EamA domain (InterPro: IPR000620), often associated to drug/metabolite transporters. The two ABC transporters identified, if related to heme uptake, could be downregulated by the parasite when heme is available, like *TcHRG*. Indeed, intracellular heme levels were shown to decrease upon treatment with ABC transporter–specific inhibitors, and this deficit could not be rescued by heme supplementation 39.

Two peptidases were also downregulated, a calpain-like cysteine peptidase (C4B63_69g318c), and a glutamamyl carboxypeptidase (C4B63_462g9). The latter is predicted to encode an enzyme with acetylornithine deacetylase (AOD) activity, typically associated with arginine biosynthesis. Interestingly, an AOD from *Arabidopsis thaliana* can function as a Cys-Gly dipeptidase implicated in the glutathione degradation pathway 35. Notably, our initial analysis identified seven genes encoding glutamyl carboxypeptidase homologs. Although glutathione and trypanothione catabolism remain poorly characterized in *T. cruzi*, it is tempting to speculate that this enzyme might play an analogous role in thiol metabolism. Their downregulation, together with the upregulation of trypanothione biosynthetic genes observed in our second analysis, could reflect a coordinated metabolic response to oxidative stress or altered redox balance.

On the other hand, the peptides encoded by C4B63_24g988c and C4B63_69g318c—which share 95% amino acid sequence identity—are classified as dimerization-anchoring domains from a cAMP-dependent protein kinase (Supfam: SSF47391) and may be components of a signaling pathway involved in sensing and responding to heme depletion. Finally, the analysis also identified a conserved hypothetical gene (C4B63_46g80); however, no relevant information is available for it.

The upregulated DEGs identified in this analysis include an amino acid permease transporter (C4B63_70g116) likely belonging to the Amino acid-Polyamine-Organocation family, a pyridoxal kinase (C4B63_42g103), the glycolytic enzyme enolase (C4B63_47g72), a mitochondrial hydroxymethylglutaryl-CoA lyase (C4B63_7g135), associated with ketogenesis and leucine catabolism, and a conserved hypothetical gene (C4B63_44g117) containing a putative SPRY domain (InterPro: IPR003877), known to participate in key signaling pathways, such as RNA processing and regulation of histone methylation.

All DEGs identified here were also detected in the second analysis, except for hydroxymethylglutaryl-CoA lyase and the amino acid permease transporter genes. Additionally, genes encoding CRAL/TRIO domain-containing protein, *Tc*HRG, RNA-binding protein, glutamamyl carboxypeptidase, SPRY domain-containing protein, and the hypothetical dimerization-anchoring domains of cAMP-dependent protein kinases were also found in our first global analysis, further highlighting the consistency and robustness of our results.

**Figure 6:**
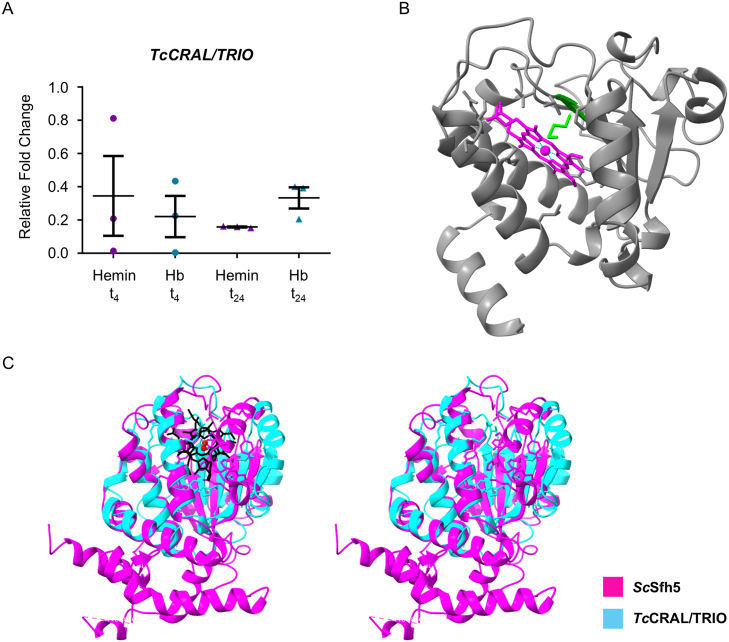
**(A)** Quantification of *TcCRAL/TRIO* mRNA levels in epimastigotes cultured in medium supplemented with 5 μ M hemin or 1.25 μ M hemoglobin (Hb) for 4 h (t ${}_{4}$ ) or 24 h (t ${}_{24}$ ). Quantification was performed by RT-qPCR, using *TcUbiquitin* as the reference gene. Data represent the mean ± SD of relative mRNA levels of three biological replicas. mRNA levels were relativized to those of no heme t ${}_{4}$ sample (for hemin and Hb t ${}_{4}$ samples) and no heme t ${}_{24}$ sample (for hemin and Hb t ${}_{24}$ samples). **(B)** Predicted 3D structure of *Tc*CRAL/TRIO in complex with heme, generated using AlphaFold. In green is highlighted the Met46, a potential axial ligand. **(C)** Structural overlay of the predicted *Tc*CRAL/TRIO (cyan) with the crystal structure of Sfh5 from *S. cerevisiae* (pink), shown in complex with heme (left panel) and without heme (right panel) to facilitate visualization. Heme molecules are represented in black, and the central Fe ions in red. The sequence alignment score obtained was 278.6, indicating moderate sequence similarity. Global RMSD was 6.02 Å across all 174 aligned pairs, and 1.06 Å over 61 well-aligned core residues.

To validate the RNA-seq results, we performed RT-qPCR analysis of five representative genes. Four of them—*TcHRG*, *TcRBP5*, *TcFR*, and *TcCRAL/TRIO*—showed downregulation consistent with the transcriptomic data. We also examined *TcCOX10*, a gene that did not appear as differentially expressed in the RNA-seq dataset, and likewise observed no significant change in its mRNA levels by RT-qPCR. *TcCRAL/TRIO* is presented in detail in the following section (Figure 6**A**), while the remaining validations are shown in Supplementary Figure S3. The concordance between both approaches reinforces the robustness and reliability of our RNA-seq analysis.

### *TcCRAL/TRIO* is a heme responsive gene encoding a hemoprotein

While SEC14 family proteins are generally known for binding small lipids such as phosphatidylinositol, Khan *et al.* recently characterized a Sec14-like phosphatidylinositol transfer protein from *Saccharomyces cerevisiae*, named Sec-Fourteen Homolog 5 (*Sc*Sfh5, UniProt ID: A6ZQI5) that, unexpectedly, binds heme instead 36. It has been proposed that *Sc*Sfh5 functions in redox control and/or the regulation of heme homeostasis under stress conditions induced by organic oxidants. Considering this, we performed a series of assays to characterize the related protein encoded by the C4B63_18g39 (named here: *T. cruzi* CRAL/TRIO domain-containing protein, *Tc*CRAL/TRIO). *TcCRAL/TRIO* encodes a 183 amino acids protein and presents a logFC greater than −1.7 in hemin and −1.4 in Hb conditions compared to no heme at t ${}_{4}$ .

To characterize *Tc*CRAL/TRIO, we analyzed its gene expression by RT-qPCR. As shown in Figure 6**A**, *TcCRAL/TRIO* presented a lower expression for hemin and Hb replenished cultures compared to heme-deprived ones both at t ${}_{4}$ and t ${}_{24}$ , thus validating RNA-seq assay results.

Then, we predicted its 3D structure using AlphaFold3 37, which suggests its potential to coordinate heme. The five AlphaFold-predicted models (Figure S4) exhibited reliable structural features, supported by high-ranking confidence scores—ranking values ranging from 0.80 to 0.86, iPTM scores between 0.78 and 0.85, and pTM scores from 0.88 to 0.89—indicating good overall model quality (Table S7). Figure 6**B** shows the highest-ranked model. Met46, highlighted in green, is positioned as a potential axial heme ligand, with distances to the heme iron (2.0 Å and 1.4 Å) inferred from the Predicted Aligned Error (PAE)–based structural analysis. Despite sharing only 27.6% amino acid sequence identity and showing moderate global structural similarity to the crystal structure of *Sc*Sfh5 (PDB ID: 6W32) 36, the predicted *Tc*CRAL/TRIO model retains the characteristic CRAL/TRIO domain architecture (Figure 6**C**).

**Figure 7:**
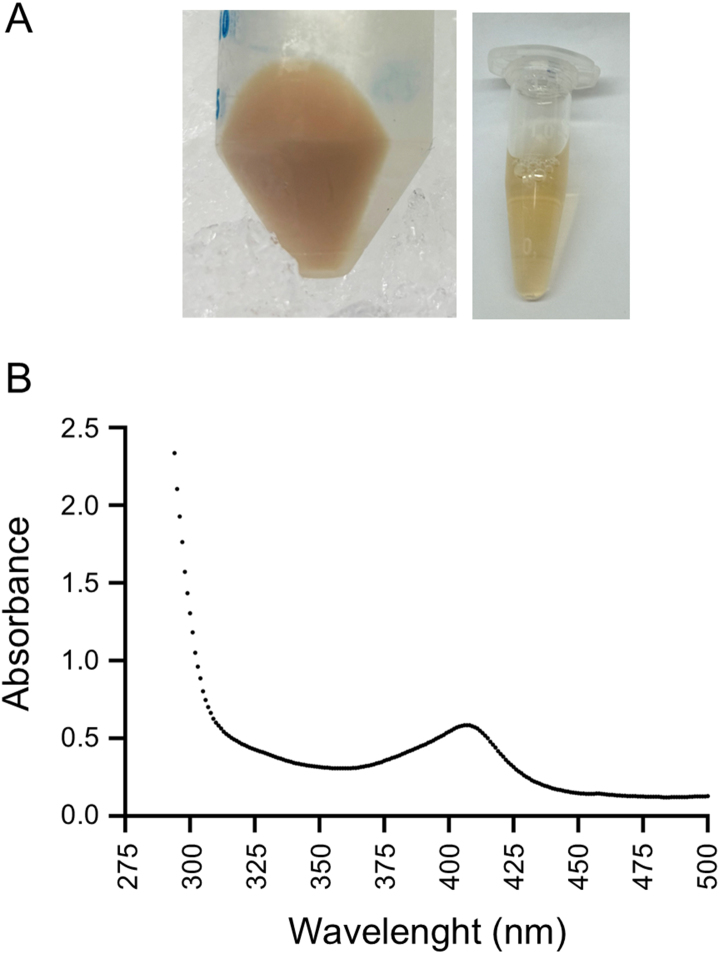
Heterologous expression and purification of *Tc*CRAL/TRIO. ** (A) ***E. coli* cell pellet expressing *Tc*CRAL/TRIO as a fusion to Maltose Binding Protein (MBP) (left panel) and the corresponding purified *Tc*CRAL/TRIO protein (right panel). **(B)** UV–visible absorption spectrum of the purified *Tc*CRAL/TRIO, showing a characteristic Soret peak at 407 nm.

Finally, we expressed *Tc*CRAL/TRIO in *Escherichia coli* as a fusion protein with Maltose Binding Protein (MBP) and a His-tag, followed by purification using Ni-NTA affinity chromatography. Both the bacterial pellet and the purified recombinant protein displayed a characteristic brownish color (Figure 7**A**), and UV-Vis spectrophotometric analysis of the purified recombinant *Tc*CRAL/TRIO revealed a Soret peak at 407 nm (Figure 7**B**) consistent with the presence of bound heme 38.

Altogether, these results constitute the first evidence of a heme responsive gene that encodes a hemoprotein in *T. cruzi* and suggest a potential involvement in heme homeostasis. However, its precise role—whether in transport, buffering, or sensing—remains to be elucidated.

## CONCLUSION

*T. cruzi* requires heme as an essential cofactor but must maintain its strict homeostatic control due to its potential toxicity. The parasite navigates a complex environment, encountering distinct heme sources depending on its lifecycle stage. Epimastigotes, the focus of this study, face free hemin and Hb in the insect midgut. Acquisition of heme occurs during replicative stages 4, mediated by *Tc*HRG 8, 9. Instead, the intracellular amastigote stage, encounters labile or exchangeable heme 10, 11 and host hemoproteins. Managing this essential cofactor/toxin dualism and adapting to fluctuations in its availability are critical for parasite survival.

Our transcriptomic analysis in *T. cruzi* epimastigotes 14 delineates the parasite’s adaptive mechanisms to both hemin and Hb. A core response common to both sources reflects a dual strategy to manage heme toxicity: (1) rapid induction of ROS-scavenging systems for detoxification, and (2) metabolic adjustments to minimize further reactive oxygen species production.

Source-specific responses further highlight distinct acquisition and signaling pathways. The upregulation of cruzipain genes under Hb exposure reinforces the model that proteolytic processing is a prerequisite for Hb-derived heme uptake 9. Conversely, the genes selectively modulated by hemin support its role as a key metabolic signal that regulates metacyclogenesis, agreeing with the model that heme depletion activates the TcK2 kinase 19.

Our study also includes the first spectroscopic characterization of a heme-binding protein encoded by a heme-responsive gene in *T. cruzi*, the CRAL/TRIO domain-containing protein (*Tc*CRAL/TRIO). Its identification as a hemoprotein, similar to Sfh5 from *S. cerevisiae* 36, strongly suggests a potential involvement in heme homeostasis.

In sum, this work provides a comprehensive view of how *T. cruzi* adapts to different heme sources, identifying both conserved regulatory elements and source-specific adaptations. The results provide a foundation for future exploration of heme management in human-infective stages, such as intracellular amastigotes, and offer a promising starting point for identifying key vulnerabilities that could inform new strategies to control parasite proliferation.

## MATERIALS AND METHODS

### Reagents

Hemin (Frontier Scientific, Logan, UT, USA) and Hb (Sigma, Burlington, MA, USA) stock solutions were prepared, quantified and stored as described 9. Fetal Bovine Serum (FBS) (Internegocios SA, Mercedes, BA, Argentina) was subjected to heat inactivation for 30 minutes at 56 ${}^{\circ }$ C prior to use. Luria-Bertani broth (LB), Liver infusion broth and tryptose broth were purchased from BD Difco (Franklin Lakes, NJ, USA). All reagents used were of molecular biology grade.

### Parasite culture and experimental design

*T. cruzi* epimastigotes (Dm28c strain) were cultured at 28 ${}^{\circ }$ C in Liver Infusion Tryptose (LIT) medium supplemented with 10% heat inactivated FBS (LIT-10% FBS) plus 5 μ M hemin and maintained in mid-log phase by successive dilutions in fresh medium 8.

For heme starvation, epimastigotes were collected, washed twice with PBS, resuspended in fresh medium without heme (at 30 × 10 ${}^{6}$ epimastigotes/mL), and maintained for 48 hours. Then, parasites were harvested, washed twice with PBS, and resuspended at 30 × 10 ${}^{6}$ epimastigotes/mL in fresh medium without heme or containing 5 μ M hemin or 1.25 μ M Hb.

Samples were taken at different time points for western blotting, RT-qPCR, and RNA-seq, as specified below. Parasites were counted using the Neubauer chamber and a Counter 19 Auto Hematology Analyzer (Wiener Laboratorios SAIC, Rosario, SF, Argentina) set up for parasite number measurements.

### Western blot

Samples for Western blotting were prepared and processed as previously described 9, including the use of the same primary 8 and secondary antibodies for *Tc*HRG detection and protein loading evaluation, except for the use of monoclonal Mouse anti- α -tubulin, Invitrogen™ #322500 (1/1000).

### Quantitative real-time PCR (RT-qPCR)

Total mRNA isolation and quantification was carried out as described previously 8, 9. RNA was treated with DNAse (RQ1 RNAsa-Free Dnase, Promega, Madison, WS, USA) and retrotranscribed with FireScript KIT Reverse Transcriptase (Solis BioDyne, Tartu, Estonia) to obtain cDNA. qPCRs were performed with HOT FIREPol EvaGreen qPCR Mix Plus (ROX) (Solis BioDyne, Tartu, Estonia), using the primers listed below (Macrogen, Korea), in a StepOne (Applied Biosystem, ThermoFisher Scientific, Foster city, CA, USA) or, alternatively, a CFX Opus 96 Real-Time PCR System (#12011319 - Bio-Rad, Miami, FL, USA) following the protocol: 95 ${}^{\circ }$ C (10 min); 40 cycles of 95 ${}^{\circ }$ C (10 s), 60 ${}^{\circ }$ C (30 s), and 72 ${}^{\circ }$ C (20 s); followed by the denaturation curve to analyze the Tm (melting temperatures) of the products. The efficiency of each pair of primers was determined, and the relative fold changes were calculated by the 2 ${}^{\mathrm{-\Delta \Delta Ct}}$ method  40.

The primers used for RT-qPCR were (accession numbers, between brackets): *TcHRG* (TcCLB.511071.190) Fw: TAATTATTGGGCGGCGGCT, Rv: GAAGTACGAACTCCCCGTCC; *TcCRAL/TRIO* (C4B63_18g39) Fw: GTTGGGTGCTCTTCTTCTCT, Rv: ACCTTCTTTCGGGTACGTTT; *TcGAPDH* (TcCLB.506943.50) Fw: GTGGCAGCACCGGTAACG, Rv: CAGGTCTTTCTTTTGCGAATAGG and *TcUbiquitin* (BCY84_18965) Fw: AGGGCATTCCGGGAAAGATG, Rv: CCACCACCATGTGCAGAGTT, based on nucleotide sequences found on the annotated genomes of *T. cruzi* CL Brenner Esmeraldo-like (*TcHRG*, *TcGAPDH*), Dm28c strain 2017 (*TcUbiquitin*) and Dm28c strain 2018 (*TcCRAL/TRIO*).

For *TcHRG* mRNA quantification, *TcGAPDH* was used as reference (housekeeping) for sample normalization, and the value of starved epimastigote (t ${}_{0}$ ) was assigned as mRNA level = 1 (reference). For *TcCRAL/TRIO* mRNA quantification, *TcUbiquitin* was used as reference for sample normalization, and cultures incubated without heme at the corresponding time points were used as the reference condition (mRNA level = 1).

### RNAseq assay

Three biological replicas were performed, from which triplicate samples were taken at the established time points for the heme-deprived, hemin and Hb conditions. Total RNA was obtained from these samples, and one complete set was subjected to RNAseq assay (Macrogen, Inc., Republic of Korea) while the other two were backed up for latter validation by RT-qPCR assays.

The transcriptome of the samples was sequenced in a Next Generation Sequencing (NGS) platform. Libraries were synthesized with Illumina TruSeq total RNA sample preparation kit with Ribo-Zero Gold. Sequencing was performed with Illumina NovaSeq6000 technology, selecting paired-end reads of 101 pb length.

### Analysis of RNA-seq data

The quality of raw reads obtained from the RNA-seq assay was assessed using *FastQC* 41. Adapter trimming and low-quality base removal were performed using *sickle*. Reads from each library were aligned to the *Trypanosoma cruzi* Dm28c 2018 reference genome (available at *TriTrypDB*: https://tritrypdb.org) using *Bowtie2* 42, with the “end-to-end” alignment mode. The resulting SAM alignment files were converted to BAM format and indexed using *SAMtools* 43 for downstream processing and to obtain mapping statistics.

Gene-level quantification was performed using *featureCounts* 44, restricting counting to coding sequences (CDS). A global count matrix was generated, and differential gene expression analysis (DEG) was conducted in R using the *DESeq2* package 45.

Normalized counts were visualized using heatmaps generated with the *pheatmap* package. Additional data exploration and visualization were carried out using R packages including *dplyr*, *ggplot2*, *RColorBrewer*, *ggrepel*, *stringr*, *hrbrthemes*, *GGally*, and *viridis*.

Gene Ontology (GO) enrichment analysis was conducted using the tools available on the TriTrypDB platform, with statistical significance determined by a Bonferroni-adjusted p-value threshold of < 0.1. Redundant GO terms were removed by manual curation to enhance result clarity. The results were visualized as bar plots generated in R with custom scripts.

### *Tc*CRAL/TRIO expression and purification

A pET32 plasmid construct 46 encoding the 6His–MBP–TEV–*Tc*CRAL/TRIO fusion protein was transformed into *E. coli* BL21 (DE3) pLysS cells. Expression was induced with 100 μ M IPTG and carried out overnight at 20 ${}^{\circ }$ C. Cells were resuspended in Lysis Buffer (100 mM HEPES, 1 M NaCl, 2% (v/v) Triton X-100, 2 mM PMSF, pH 7.4) and lysed using a 750 W ultrasonic processor (Cole Parmer, Vernon Hills, IL, USA). The clarified lysate was incubated with Ni Sepharose™ 6 Fast Flow resin (GE Healthcare, Chicago, IL, USA) in the presence of Binding Buffer (100 mM HEPES, 1 M NaCl, 30 mM imidazole, pH 7.4), followed by a washing step using the same buffer. The His-tagged protein was eluted with buffer containing 250 mM imidazole and analyzed by SDS-PAGE with Coomassie staining. UV–visible spectra of the eluates were recorded using a GeneQuant 1300 spectrophotometer in quartz cuvettes.

### *In silico* analysis

Amino acid sequence alignments were performed using Clustal Omega 48. The predicted 3D structure of *Tc*CRAL/TRIO bound to heme was generated using the AlphaFold algorithm via the AlphaFoldServer web service (https://alphafoldserver.com/), following the method described by Jumper *et al*. 37. Structural visualization, model overlay, and comparison with the crystal structure of *S. cerevisiae* Sfh5 were performed using ChimeraX 49.

### Statistical analysis

Western blot analysis to assess *Tc*HRG protein levels were independently reproduced two times. RT-qPCR analysis was independently reproduced three times. Statistically significant differences between groups in the RT-qPCR data were determined using GraphPad Prism version 6.00 for Windows (GraphPad Software, San Diego, CA).

Transcriptomic analysis was performed using three independent biological replicas. Quantification of *Tc*CRAL/TRIO mRNA was conducted on samples taken from the same cultures used for the transcriptomic assay. *Tc*CRAL/TRIO protein purification experiments were independently reproduced at least three times.

## DATA AVAILABILITY

All relevant data supporting this study are included in the main article and supplementary material. The complete set of raw data has been deposited into the Repositorio de Datos Académicos UNR (RDA UNR) https://dataverse.unr.edu.ar/, the institutional data repository of the Universidad Nacional de Rosario. This open-access platform enables the dissemination and long-term preservation of academic research data, adheres to FAIR principles and is built on Dataverse Project technology. Additionally, the dataset is assigned a persistent and unique Digital Object Identifier (DOI) provided by RDA UNR number (https://doi.org/10.57715/UNR/BPKC2V).

## SUPPLEMENTAL MATERIAL

All supplemental data for this article are available online at http://microbialcell.com/researcharticles/2026a-tevere-microbial-cell/. 

## CONFLICT OF INTEREST

The authors declare no conflict of interest.

## ABBREVIATIONS

ANOVA – analysis of variance

DEG – differentially expressed gene

FBS – fetal bovine serum

GO – gene ontology

Hb – hemoglobin

LIT – liver infusion tryptose

MBP – maltose binding protein

PBS – phosphate buffered saline

RNA-Seq – RNA sequencing

ROS – reactive oxygen species

RT-qPCR – reverse transcription followed by quantitative real-time PCR

*Tc*HRG – *Trypanosoma cruzi* Heme Responsive Gene
