# Supplementary material for: Transcriptomic response to different heme sources in Trypanosoma cruzi epimastigotes
Source: Microb Cell. 2026 Jan 23;13:13–27. doi: 10.15698/mic2026.01.865 (PMC12834515; doi:10.15698/mic2026.01.865)
Supplement: Supplementary file 1 [file mic-13-013-s01.pdf]

Figure S1

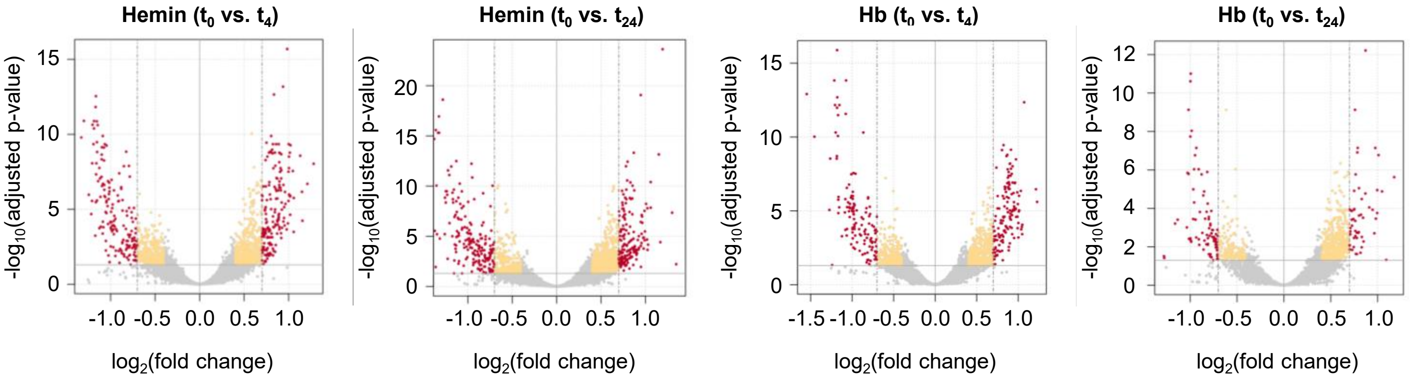

Figure S1. Volcano plots showing differentially expressed genes (DEGs) across various culture conditions and time points. Each plot displays $\log_2$(fold change) vs. $-\log_{10}$(adjusted p-value) for genes identified under hemin and hemoglobin (Hb) conditions. Timepoint comparisons include t_4 vs. t_0 and t_{24} vs. t_0. Genes with $|\log_2(\text{fold change})| < 0.4$ and $-\log_{10}$(adjusted p-value) < 1.3 are shown in gray (not significantly regulated). Genes with $0.4 \leq |\log_2(\text{fold change})| \leq 0.7$ and $-\log_{10}$(adjusted p-value) > 1.3 are shown in yellow. Genes with $|\log_2(\text{fold change})| > 0.7$ and $-\log_{10}$(adjusted p-value) > 1.3 are shown in red.

Figure S2

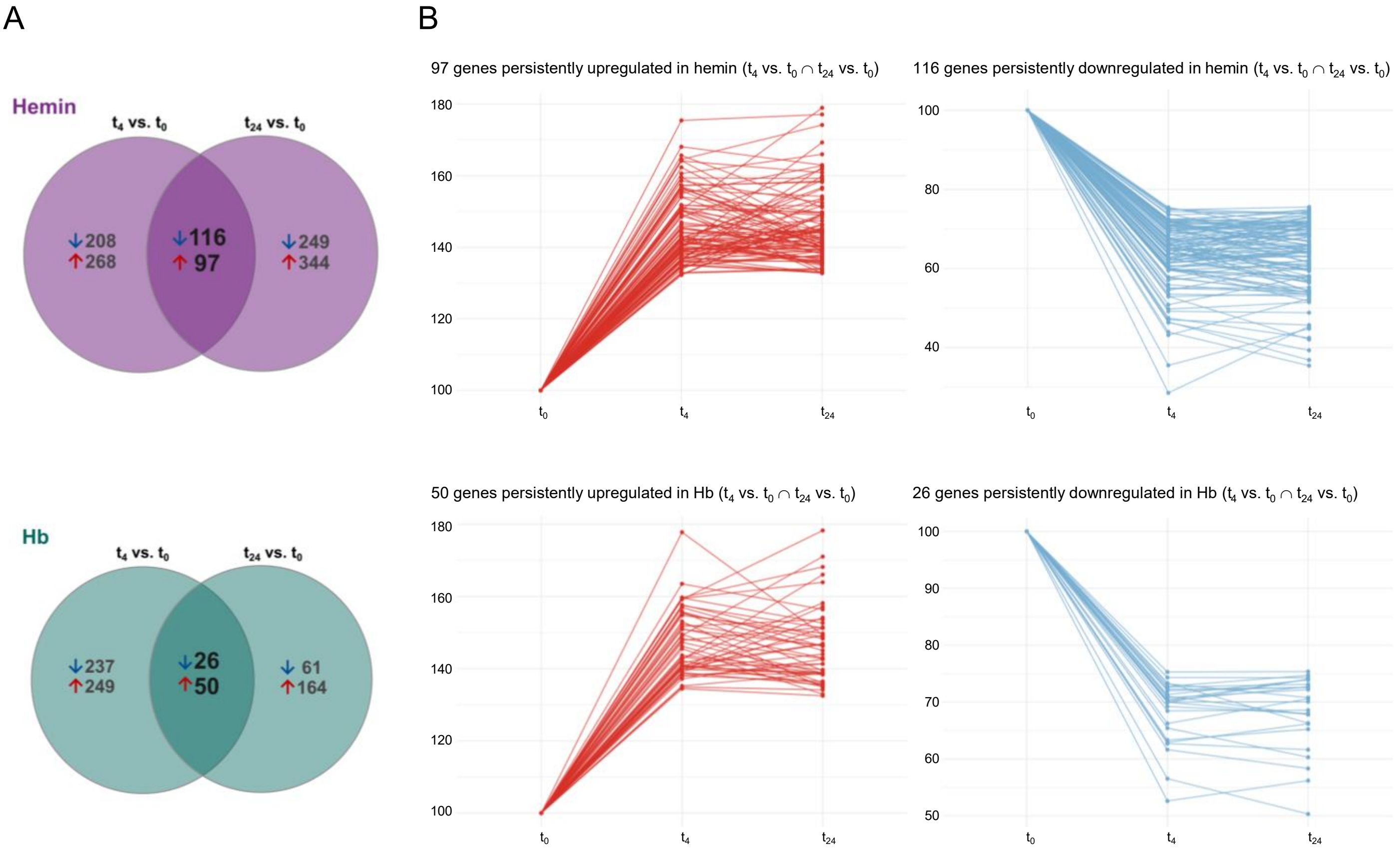

Figure S2. Persistent differential expression of genes in hemin and hemoglobin (Hb) conditions from t_4 to t_{24}. **(A)** Venn diagrams showing shared differentially expressed genes between the t_4 vs. t_0 and t_{24} vs. t_0 comparisons in hemin and Hb-supplemented parasites. **(B)** Expression profiles over time of upregulated (red) and downregulated (blue) genes that exhibit sustained differential expression in hemin- and Hb-supplemented parasites.

Figure S3

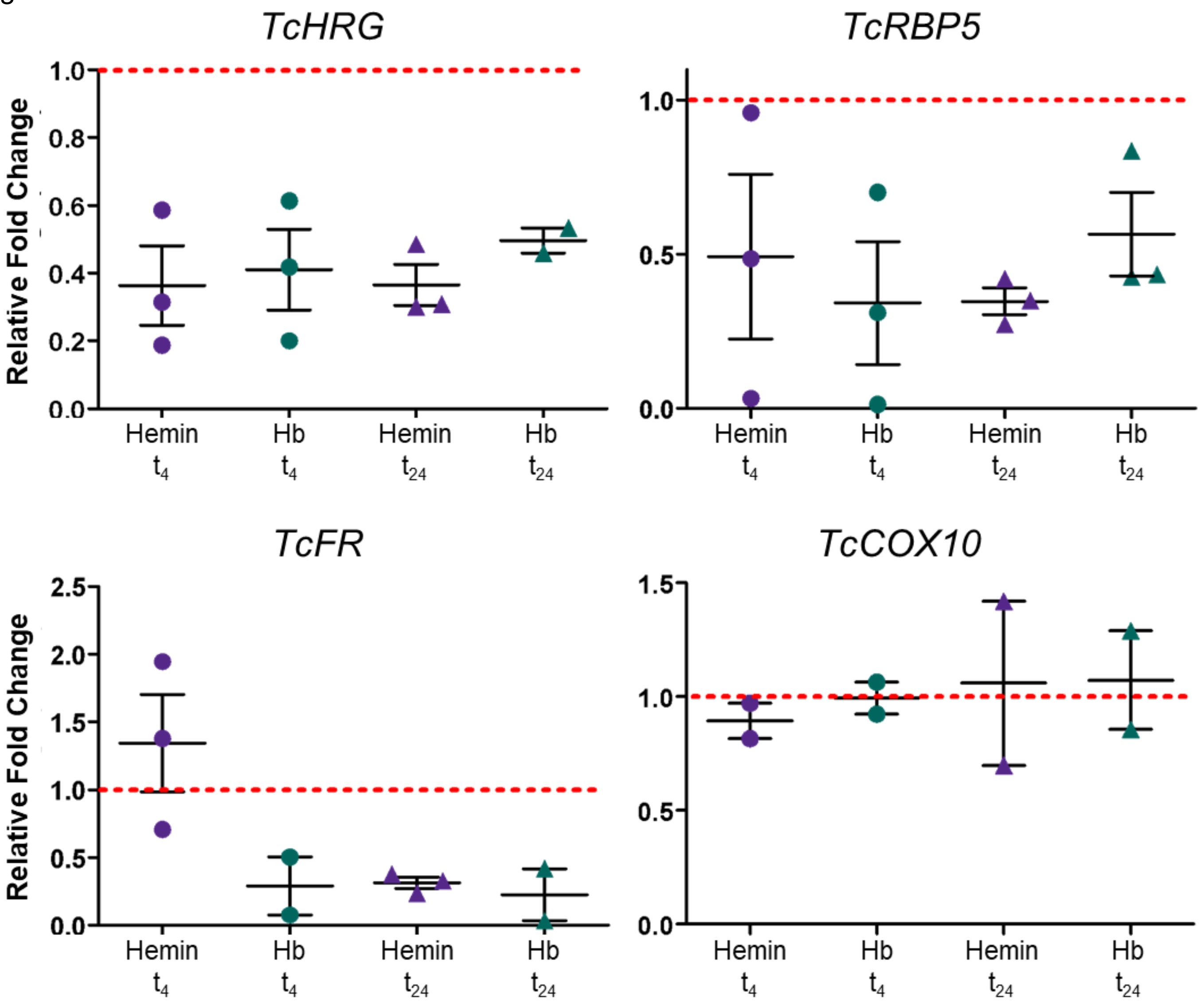

Figure S3. ***TcHRG***, ***TcRBP5***, ***TcFR***, and ***TcCOX10*** mRNA levels in hemin- or Hb-refed epimastigotes relative to the no-heme condition. Total RNA was obtained as previously described (Tevere, 2023 and Pagura, 2020). For each sample, 10^8 parasites were collected, and RNA extraction was performed using 1 mL TRI Reagent® following the manufacturer instructions (TR118 - Molecular Research Center, Inc., Cincinnati, OH, USA). The quality of RNA was verified in agarose gels and quantified by absorbance. The RNA samples were treated with DNAse (RQ1 Rnasa-Free Dnase, Promega, Madison, WS, USA) and retrotranscribed with FireScript KIT Reverse Transcriptase (Solis BioDyne, Tartu, Estonia) to obtain cDNA. The quantitative Real Time PCRs (qPCRs) were performed with HOT FIREPol EvaGreen qPCR Mix Plus (ROX) (Solis BioDyne, Tartu, Estonia), using the primers listed below (Macrogen, Korea), in a StepOne (Applied Biosystem, ThermoFisher Scientific, Foster city, CA, USA) or, alternatively, in a CFX Opus 96 Real-Time PCR System (#12011319 - Bio-Rad, Miami, FL, USA) following the following protocol: 95 °C (10 min); 40 cycles of 95 °C (10 s), 60 °C (30 s), and 72 °C (20 s); followed by the denaturation curve to analyze the Tm (melting temperature) of the products. The efficiency of each pair of primers was determined, and the relative fold changes were calculated by the $2^{-\Delta\Delta Ct}$ method. *TcUbiquitin* gene was used as reference (housekeeping) for sample normalization and the starved epimastigote sample (t_0) was taken as reference (mRNA level = 1). Results are expressed as mRNA levels in Hemin- and Hb-refed samples relative to those in No heme samples at the same time points: Hemin t_4/No heme t_4 (Hemin t_4), Hemin t_{24}/No heme t_{24} (Hemin t_{24}), Hb t_4/No heme t_4 (Hb t_4) and Hemin t_{24}/No heme t_{24} (Hb t_{24}). Data represents three independent biological replicates (each measured in triplicate), except for Hb t_{24} of *TcHRG*, *TcFR*, and *TcCOX10*, and Hemin t_{24} of *TcCOX10*, which were obtained from two biological replicates (each measured in triplicate). Primer design was based on nucleotide sequences of *T. cruzi* CL Brenner Esmeraldo-like (*TcHRG* and *TcCOX10*) and Dm28c strain 2017 (*TcUbiquitin*) and 2018 (*TcRBP5*) genes; with the respective accession numbers given between brackets at the following primer list. The sequences of the primers used were: *TcHRG* (TcCLB.511071.190) Fw: TAATTATTGGGCGGCGGCT, Rv: GAAGTACGAACTCCCCGTCC; *TcRBP5* (C4B63_27g1020c) Fw: GAAGCATCGGCAAAAAGGAT, Rv: CGGTTGAGGGACTAACAAGA; *TcFR* (BCY84_1374, BCY84_05177, BCY84_05181, BCY84_05183, BCY84_05184) Fw: CTGTCGTTGCTCATGTTTCC, Rv: TTTGTGGACCCAGATCGAAG; *TcCOX10* (Tc00.1047053509767.59): Fw: AGATGAAGCGAACCTGTCGT, Rv: AACCACAAGCTCCAAACCAC; and *TcUbiquitin* (BCY84_18965) Fw: AGGGCATTCCGGGAAAGATG, Rv: CCACCACCATGTGCAGAGTT.

Figure S4

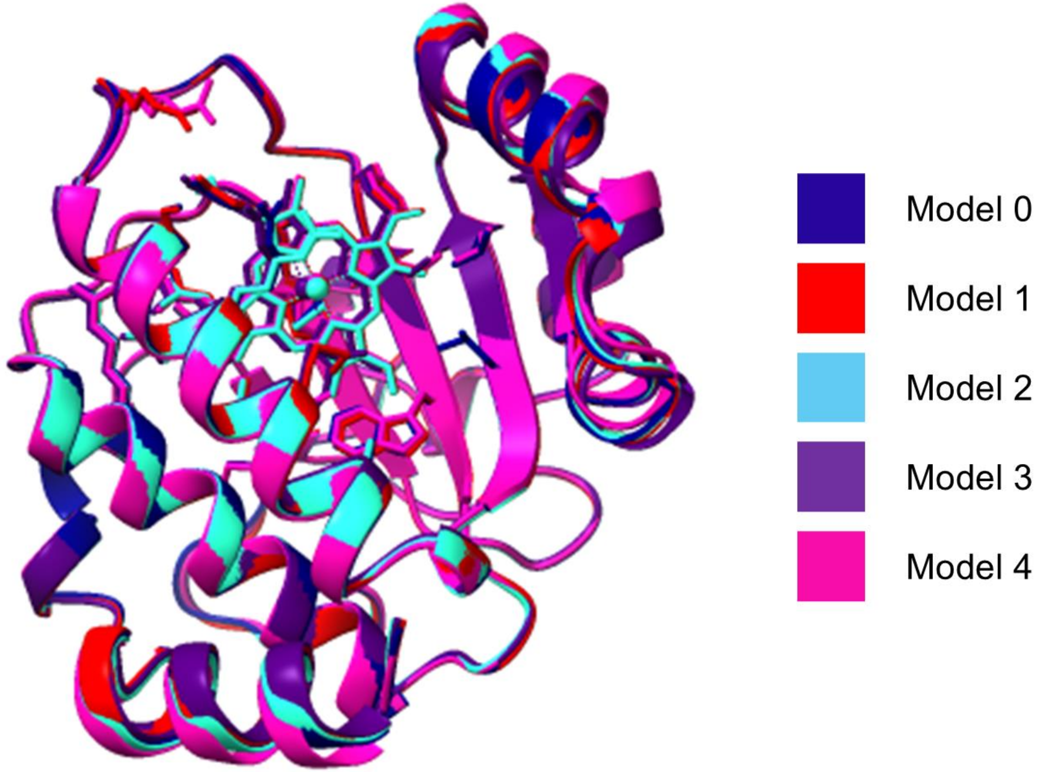

Figure S4. Structural overlay of the five 3D structures of *Tc*CRAL/TRIO predicted by Alphafold. Raw output of confidence scores from AlphaFold for the five predicted 3D models of *Tc*CRAL/TRIO at UNR RDA (https://doi.org/10.57715/UNR/BPKC2V).

Table S1

DEGs (heme-supplemented vs. heme-starved)				
ID	Annotation	log2FC	pvalue	padj
C4B63_3g227	mucin TcMUCII	-0.32993493357209	0.00011690440045204	0.0405268588233739
C4B63_462g11	glutamamyl carboxypeptidase	-0.607368478302711	4.24019511270686e-05	0.0229309751695187
C4B63_322g15	amino acid transporter	0.234546086764434	3.58640665365175e-05	0.0229309751695187
C4B63_27g1020c	RNA-binding protein	-0.895786305604263	4.14698768510786e-14	2.80336367513291e-10
C4B63_70g88	ATG16/SAP18/CVT11/APG16	-0.249169217702849	1.02047991183225e-06	0.00197098405828172
C4B63_462g9	glutamamyl carboxypeptidase(fragment)	-0.662535100092884	5.90494491750712e-05	0.0262631409519877
C4B63_313g13	amino acid transporter	0.203116115381241	0.00010988706767567	0.0405268588233739
C4B63_27g184	integral membrane transport protein	-0.51462531309972	1.20892719481177e-14	1.63446956738551e-10
C4B63_462g10	glutamamyl carboxypeptidase	-0.602732341729182	0.000105610670822974	0.0405268588233739
C4B63_313g15	amino acid transporter	0.239765565807958	1.40756608262442e-05	0.0126868622913881
C4B63_350g5	glutamamyl carboxypeptidase(fragment)	-0.542200227815286	0.000155766739039081	0.0438742981626743
C4B63_124g19	surface protease GP63	0.339239427118475	0.000136428336729589	0.0434440241343718
C4B63_257g5	amino acid transporter	0.192040528159319	5.73521667723818e-05	0.0262631409519877
C4B63_282g15	cation transporter	0.354507535145914	3.33892826515104e-07	0.000902846202896841
C4B63_313g11	amino acid transporter	0.224776052719636	0.000159036340427043	0.0438810473994617
C4B63_4g180	serine/threonine protein kinase	-0.326450422207995	6.02187403484926e-05	0.0262631409519877
C4B63_6g236	cation transporter	0.344007513437361	2.72284475063006e-06	0.0040903178920576
C4B63_51g83	ferric reductase transmembrane protein	-0.387411987873575	4.22180308878258e-05	0.0229309751695187
C4B63_2g799	tRNA wybutosine-synthesizing protein 4	0.202922242722498	6.35602707145551e-05	0.0268542143768995
C4B63_336g4	glutamamyl carboxypeptidase	-0.592696848892484	5.52795498147318e-05	0.0262631409519877
C4B63_98g22	trans-sialidase	-0.529646115656052	0.000127349481582203	0.0419942680729605
C4B63_550g1	metallo-peptidase, clan MH, family M18	-0.619000120633786	0.00014449547263427	0.0434440241343718
C4B63_34g375	Soluble NSF attachment protein, SNAP (paraflagelar rod component)	-0.225841931522567	4.12696463479452e-05	0.0229309751695187
C4B63_20g314	serine/threonine-protein phosphatase PP1	-0.193009470011326	0.000140162109283095	0.0434440241343718
C4B63_42g103	pyridoxal kinase	0.341502061627782	0.000123297045199435	0.0416744012774091
C4B63_19g205	glycosomal transporter (GAT3)	0.28984712801925	3.65675309403849e-06	0.00426790262536025
C4B63_257g6	amino acid transporter	0.216358150969246	1.38970948346949e-05	0.0126868622913881
C4B63_40g93	conserved hypothetical protein	-0.231767513706	0.000155321486507511	0.0438742981626743
C4B63_97g13	conserved hypothetical protein	-0.276476125660578	0.000185029265017814	0.0490508953537422
C4B63_313g14	amino acid transporter	0.272532299551567	2.08137954740411e-06	0.00351753143511294
C4B63_258g25	lipase	-0.415942368533025	8.78493093583167e-05	0.034933019486013
C4B63_25g220	5'-3' exoribonuclease B	-0.315531752492431	0.00011152169768915	0.0405268588233739
C4B63_51g81	ferric reductase transmembrane protein	-0.377068931146649	0.000150804550091205	0.0438742981626743
C4B63_51g82	ferric reductase transmembrane protein	-0.375293796747228	5.42215317315641e-05	0.0262631409519877
C4B63_322g14	amino acid transporter	0.247598942062281	1.71663653409714e-05	0.0145055787131208
C4B63_44g117	conserved hypothetical protein	0.433996675126999	2.86095811455705e-05	0.0203579756362165
C4B63_3g1092	alanine racemase	-0.322947119951655	1.9330081790921e-05	0.015373100341956
C4B63_69g318c	conserved hypothetical protein	-0.48388155806861	8.03778715482471e-06	0.0083592986410177
C4B63_313g10	amino acid transporter	0.230611418184654	2.76686277567821e-05	0.0203579756362165
C4B63_18g39	CRAL/TRIO, N-terminal domain/CRAL/TRIO domain containing protein	-1.3228074513183	6.81009516320881e-08	0.000230181216516458
C4B63_6g397	cation transporter	0.315973108034771	3.38440211331048e-05	0.0228785582859788
C4B63_550g3	metallo-peptidase, clan MH, family M18	-0.601216854207726	0.00011538420281901	0.0405268588233739
C4B63_336g1	glutamamyl carboxypeptidase(fragment)	-0.48642113836089	0.0001685381192624	0.0455727074485531
C4B63_354g1	electron transfer flavoprotein	-0.414503719480781	3.77909817901243e-06	0.00426790262536025
C4B63_51g85	ferric reductase transmembrane protein	-0.379931647551028	3.86816852250706e-05	0.0229309751695187
C4B63_31g178	Zinc phosphodiesterase ELAC protein 2	0.27326856751695	5.95020306729887e-05	0.0262631409519877
C4B63_294g12	glutamamyl carboxypeptidase	-0.580367898754649	0.000144599192754936	0.0434440241343718
C4B63_32g209	Heme Responsive Gene (*Tc* HRG)	-0.487727859236573	1.21478070041169e-08	5.47461168985533e-05
C4B63_19g141	electron transfer flavoprotein	-0.402413527517146	4.29916698829893e-07	0.000968745628030025
C4B63_24g988c	conserved hypothetical protein	-0.497007686289135	3.78807925327833e-06	0.00426790262536025
C4B63_51g80	ferric reductase transmembrane protein	-0.355989571436148	6.67571892839377e-05	0.0273502181551163

Table S1. List of 51 DEGs in first global analysis.

Table S2

Hemin, Hb and No heme common DEGs (t4 vs. t0)

Downregulated

ID	Annotation
C4B63_102g376c	conserved hypothetical protein
C4B63_10g157	Double RNA binding domain protein 6A
C4B63_112g40	surface protease GP63
C4B63_129g44	cytochrome c oxidase VIII (COX VIII)
C4B63_12g242	SKP1-like protein
C4B63_12g405	cytochrome P450
C4B63_137g16	cytochrome c oxidase assembly protein
C4B63_13g200	Enriched in surface-labeled proteome protein 18
C4B63_13g327	conserved hypothetical protein
C4B63_140g32	cytochrome c oxidase VIII (COX VIII)
C4B63_14g1	hexose transporter
C4B63_14g2	hexose transporter(fragment)
C4B63_14g28	hexose transporter
C4B63_14g3	hexose transporter
C4B63_14g30	hexose transporter
C4B63_14g5	hexose transporter
C4B63_155g10	trans-sialidase
C4B63_163g21	deoxyribose-phosphate aldolase
C4B63_16g136	conserved hypothetical protein
C4B63_17g48	ubiquitin-conjugating enzyme E2
C4B63_18g1130c	FUN14 family
C4B63_196g32	Mucin-associated surface protein (MASP)
C4B63_19g86	CYC2-like cyclin 6
C4B63_1g756	surface protease GP63
C4B63_212g5	protein associated with differentiation 4(fragment)
C4B63_21g10	arginine permease
C4B63_21g14	arginine permease
C4B63_21g16	arginine permease
C4B63_21g18	arginine permease
C4B63_21g20	arginine permease
C4B63_21g21	arginine permease
C4B63_21g5	arginine permease
C4B63_21g56	nucleoside phosphorylase
C4B63_21g65	conserved hypothetical protein
C4B63_21g9	arginine permease
C4B63_228g46	glutamamyl carboxypeptidase
C4B63_228g47	glutamamyl carboxypeptidase
C4B63_228g48	glutamamyl carboxypeptidase
C4B63_228g49	glutamamyl carboxypeptidase
C4B63_228g50	glutamamyl carboxypeptidase
C4B63_228g51	glutamamyl carboxypeptidase(fragment)
C4B63_228g52	glutamamyl carboxypeptidase(fragment)
C4B63_22g743c	calpain-like cysteine peptidase
C4B63_23g16	Battenin
C4B63_23g195	3', 5'-cyclic nucleotide phosphodiesterase
C4B63_23g226	conserved hypothetical protein
C4B63_23g270	conserved hypothetical protein
C4B63_259g12	conserved hypothetical protein
C4B63_260g20	trans-sialidase, Group II
C4B63_261g5	conserved hypothetical protein
C4B63_26g240	nucleobase transporter
C4B63_26g278	arginine kinase
C4B63_26g282	arginine kinase
C4B63_26g286	arginine kinase
C4B63_277g1	glutamamyl carboxypeptidase(fragment)
C4B63_277g10	glutamamyl carboxypeptidase
C4B63_277g11	glutamamyl carboxypeptidase
C4B63_277g12	glutamamyl carboxypeptidase
C4B63_277g13	glutamamyl carboxypeptidase
C4B63_277g14	glutamamyl carboxypeptidase
C4B63_277g15	glutamamyl carboxypeptidase
C4B63_277g16	glutamamyl carboxypeptidase
C4B63_277g17	glutamamyl carboxypeptidase
C4B63_277g18	glutamamyl carboxypeptidase(fragment)
C4B63_277g2	glutamamyl carboxypeptidase
C4B63_277g20	glutamamyl carboxypeptidase(fragment)
C4B63_277g21	glutamamyl carboxypeptidase
C4B63_277g3	glutamamyl carboxypeptidase
C4B63_277g4	glutamamyl carboxypeptidase
C4B63_277g5	glutamamyl carboxypeptidase
C4B63_277g6	glutamamyl carboxypeptidase
C4B63_277g7	glutamamyl carboxypeptidase
C4B63_277g8	glutamamyl carboxypeptidase
C4B63_277g9	glutamamyl carboxypeptidase
C4B63_27g1015c	conserved hypothetical protein
C4B63_294g1	glutamamyl carboxypeptidase
C4B63_294g10	glutamamyl carboxypeptidase
C4B63_294g11	glutamamyl carboxypeptidase
C4B63_294g12	glutamamyl carboxypeptidase
C4B63_294g13	glutamamyl carboxypeptidase
C4B63_294g2	glutamamyl carboxypeptidase(fragment)
C4B63_294g5	glutamamyl carboxypeptidase
C4B63_294g6	glutamamyl carboxypeptidase
C4B63_294g7	glutamamyl carboxypeptidase
C4B63_294g8	glutamamyl carboxypeptidase
C4B63_294g9	glutamamyl carboxypeptidase
C4B63_2g435	Flagellum attachment zone protein 12
C4B63_2g549	receptor-type adenylate cyclase
C4B63_2g702	Gim5A protein
C4B63_2g704	Gim5A protein
C4B63_2g706	Gim5A protein
C4B63_30g122	surface protease GP63
C4B63_30g150	cystinosin
C4B63_30g182	Putative adenylate cyclase regulatory protein
C4B63_30g210	serine-threonine dehydratase
C4B63_30g261	deoxyribose-phosphate aldolase
C4B63_31g119	trans-sialidase, Group II
C4B63_31g93	conserved hypothetical protein
C4B63_32g206	fatty acid desaturase
C4B63_336g1	glutamamyl carboxypeptidase(fragment)
C4B63_336g2	glutamamyl carboxypeptidase(fragment)
C4B63_336g3	metallo-peptidase, clan MH, family M18(fragment)
C4B63_336g4	glutamamyl carboxypeptidase
C4B63_336g5	glutamamyl carboxypeptidase
C4B63_336g6	glutamamyl carboxypeptidase
C4B63_350g1	glutamamyl carboxypeptidase
C4B63_350g2	glutamamyl carboxypeptidase(fragment)
C4B63_350g3	glutamamyl carboxypeptidase(fragment)
C4B63_350g4	metallo-peptidase, clan MH, family M18
C4B63_350g5	glutamamyl carboxypeptidase(fragment)
C4B63_351g27c	small myristoylated protein-1
C4B63_351g3	calpain-like cysteine peptidase
C4B63_37g382	unspecified product
C4B63_37g383	unspecified product
C4B63_38g174	conserved hypothetical protein
C4B63_38g83	conserved hypothetical protein
C4B63_38g94	conserved hypothetical protein
C4B63_39g346	nucleic acid binding protein
C4B63_3g644	mucin TcMUCII
C4B63_40g186	Gamma carbonic anhydrase 3, mitochondrial
C4B63_41g1149c	conserved hypothetical protein
C4B63_431g1	glutamamyl carboxypeptidase(fragment)
C4B63_431g10	glutamamyl carboxypeptidase
C4B63_431g3	glutamamyl carboxypeptidase
C4B63_431g4	glutamamyl carboxypeptidase
C4B63_431g5	glutamamyl carboxypeptidase
C4B63_431g6	glutamamyl carboxypeptidase
C4B63_431g7	glutamamyl carboxypeptidase
C4B63_431g8	glutamamyl carboxypeptidase
C4B63_431g9	glutamamyl carboxypeptidase
C4B63_43g166	conserved hypothetical protein
C4B63_44g228	amino acid permease
C4B63_462g10	glutamamyl carboxypeptidase
C4B63_462g11	glutamamyl carboxypeptidase
C4B63_462g12	glutamamyl carboxypeptidase
C4B63_462g13	glutamamyl carboxypeptidase
C4B63_46g90	Ring finger domain containing protein
C4B63_47g75	splicing factor ptsr1 interacting protein
C4B63_485g5	glutamamyl carboxypeptidase(fragment)
C4B63_4g189	alcohol dehydrogenase
C4B63_54g61	conserved hypothetical protein
C4B63_550g1	metallo-peptidase, clan MH, family M18
C4B63_550g2	glutamamyl carboxypeptidase
C4B63_550g3	metallo-peptidase, clan MH, family M18
C4B63_550g6	glutamamyl carboxypeptidase
C4B63_55g177	conserved hypothetical protein
C4B63_58g70	hexokinase
C4B63_5g44	trans-sialidase, Group V
C4B63_63g102	hexose transporter
C4B63_63g104	hexose transporter
C4B63_63g106	hexose transporter
C4B63_63g108	hexose transporter
C4B63_63g110	hexose transporter
C4B63_67g26	trans-sialidase, Group II
C4B63_6g2366c	UPF0382 membrane protein HI 1073
C4B63_7g143	Tubulin polymerization-promoting protein family member 2
C4B63_7g145	conserved hypothetical protein
C4B63_7g361	phosphomannomutase-like protein
C4B63_7g98	Eukaryotic translation initiation factor 2-alpha kinase 2
C4B63_84g160c	paraflagellar rod component
C4B63_87g8	glutamamyl carboxypeptidase
C4B63_93g25	unspecified product
C4B63_98g22	trans-sialidase
C4B63_98g34	trans-sialidase

Upregulated

ID	Annotation
C4B63_101g36	inositol-3-phosphate synthase
C4B63_102g44	hypoxanthine-guanine phosphoribosyltransferase
C4B63_104g78	calreticulin
C4B63_10g439	heat shock protein 90
C4B63_10g449	Triple RNA binding domain protein 3
C4B63_10g517	tryptophanyl-tRNA synthetase
C4B63_113g22	heat shock protein 85(fragment)
C4B63_113g23	heat shock protein 85(fragment)
C4B63_113g24	heat shock protein 85(fragment)
C4B63_113g25	heat shock protein 85
C4B63_113g26	heat shock protein 85
C4B63_113g27	heat shock protein 85(fragment)
C4B63_113g28	heat shock protein 85
C4B63_113g29	heat shock protein 85
C4B63_113g30	heat shock protein 85
C4B63_113g31	heat shock protein 85(fragment)
C4B63_113g32	heat shock protein 85
C4B63_113g33	heat shock protein 85
C4B63_11g29	2,4-dihydroxyhept-2-ene-1,7-dioic acid aldolase
C4B63_11g40	urocanate hydratase
C4B63_11g8	succinyl-CoA synthetase alpha subunit
C4B63_11g9	eukaryotic translation initiation factor 3 subunit j
C4B63_123g35	Uncharacterized protein At3g06530
C4B63_123g36	O-6 methyl-guanine alkyl transferase
C4B63_127g35	peptidase
C4B63_12g153	metallo-peptidase, Clan MF, Family M17
C4B63_12g195	conserved hypothetical protein
C4B63_12g237	eukaryotic release factor 3
C4B63_12g295	GTP-binding nuclear protein rtb2
C4B63_133g56	heat shock protein 70 (hsp70)
C4B63_133g57	heat shock protein 70 (hsp70)
C4B63_133g58	heat shock protein 70 (hsp70)
C4B63_133g59	heat shock protein 70 (hsp70)
C4B63_133g60	heat shock protein 70 (hsp70)
C4B63_133g61	heat shock protein 70 (hsp70)
C4B63_133g62	heat shock protein 70 (hsp70)(fragment)
C4B63_133g63	heat shock protein 70 (hsp70)
C4B63_137g25	conserved hypothetical protein
C4B63_137g32	conserved hypothetical protein
C4B63_137g34	heat shock protein 70 (hsp70)(fragment)
C4B63_137g36	heat shock protein 70 (hsp70)(fragment)
C4B63_137g37	heat shock protein 70 (hsp70)(fragment)
C4B63_137g38	heat shock protein 70 (hsp70)(fragment)
C4B63_137g39	heat shock protein 70 (hsp70)
C4B63_137g40	heat shock protein 70 (hsp70)(fragment)
C4B63_137g41	heat shock protein 70 (hsp70)
C4B63_138g4	elongation factor 1-gamma (EF-1-gamma)
C4B63_13g130	ER membrane protein complex subunit 3
C4B63_13g145	eukaryotic translation initiation factor 3 subunit l
C4B63_143g20	glutamine synthetase
C4B63_143g49	cystathionine beta-synthase
C4B63_148g35	zinc-finger protein 2
C4B63_149g20	cyclophilin
C4B63_14g165	N-lysine methyltransferase SETD6
C4B63_14g204	60S acidic ribosomal protein
C4B63_14g220c	kinetoplast DNA-associated protein
C4B63_157g28	FK506-binding protein (FKBP)-type peptidyl-prolyl isomerase
C4B63_16g326	conserved hypothetical protein
C4B63_171g30	FK506-binding protein (FKBP)-type peptidyl-prolyl isomerase
C4B63_175g9	heat shock protein DNAJ
C4B63_17g101	mitochondrial carrier protein
C4B63_17g179	Importin subunit beta-3
C4B63_17g65	polypyrimidine tract-binding protein
C4B63_180g3	conserved hypothetical protein
C4B63_183g12	mitochondrial carrier protein
C4B63_184g39	heat shock protein DNAJ(fragment)
C4B63_18g164	S-adenosylmethionine synthetase
C4B63_18g204	3-hydroxy-3-methylglutaryl-CoA reductase
C4B63_18g212	valyl-tRNA synthetase
C4B63_18g260	small glutamine-rich tetratricopeptide repeat protein
C4B63_18g287	heat shock 70 kDa protein, mitochondrial precursor
C4B63_18g288	heat shock 70 kDa protein, mitochondrial precursor
C4B63_18g293	heat shock 70 kDa protein, mitochondrial precursor
C4B63_192g3	AAA ATPase
C4B63_193g9	metallo-peptidase, Clan MF, Family M17
C4B63_196g51	CDP-diacylglycerol--inositol 3-phosphatidyltransferase
C4B63_207g7	conserved hypothetical protein
C4B63_207g9	ribosome biogenesis protein
C4B63_20g172	chaperonin alpha subunit
C4B63_20g213	enoyl-CoA hydratase/isomerase family protein
C4B63_210g35	3,2-trans-enoyl-CoA isomerase, mitochondrial precursor
C4B63_212g26	conserved hypothetical protein
C4B63_214g14	UDP-Gal or UDP-GlcNAc-dependent glycosyltransferase
C4B63_220g13	cyclophilin
C4B63_22g25	Major Facilitator Superfamily
C4B63_23g109	UDP-glucose:glycoprotein glucosyltransferase
C4B63_23g110	UDP-glucose:glycoprotein glucosyltransferase
C4B63_23g248	inorganic pyrophosphatase
C4B63_23g275	ATP-dependent DEAD/H RNA helicase
C4B63_24g172	Uncharacterized protein At3g06530
C4B63_24g298	CDP-diacylglycerol--inositol 3-phosphatidyltransferase
C4B63_24g325	conserved hypothetical protein
C4B63_252g22	T-complex protein 1, beta subunit
C4B63_255g21	calreticulin
C4B63_25g192	NAA35
C4B63_25g228	Putative uridine kinase C227.14
C4B63_25g249	Protein SDA1 homolog
C4B63_25g279	J Biosynthesis Protein 2
C4B63_25g294	Thioredoxin-like
C4B63_261g22c	conserved hypothetical protein
C4B63_26g204	phosphomevalonate kinase protein
C4B63_26g205	proliferative cell nuclear antigen (PCNA)
C4B63_26g252	nucleosome assembly protein-like protein
C4B63_26g279	ABC transporter
C4B63_26g283	ABC transporter
C4B63_26g287	ABC transporter
C4B63_276g16	conserved hypothetical protein
C4B63_28g106	malic enzyme
C4B63_297g10	polypyrimidine tract-binding protein
C4B63_297g14	phenylalanyl-tRNA synthetase alpha chain
C4B63_2g174	polyadenylate-binding protein 1
C4B63_2g223	conserved hypothetical protein
C4B63_2g235	Co-chaperone protein P23
C4B63_2g305	Hsp70-Hsp90 organizing protein 3
C4B63_2g311	conserved hypothetical protein
C4B63_2g430	heat shock protein
C4B63_2g485	T-complex protein 1, beta subunit
C4B63_2g509	arginyl-tRNA synthetase
C4B63_2g510	arginyl-tRNA synthetase
C4B63_2g525	ribosome biogenesis protein MAK21
C4B63_2g690	chaperonin alpha subunit
C4B63_2g90	Nop53 (60S ribosomal biogenesis)
C4B63_300g102c	mucin TcSMUGL
C4B63_304g12	conserved hypothetical protein
C4B63_304g13	nucleosome assembly protein
C4B63_30g161	glutamine synthetase
C4B63_30g290	mercaptopyruvate sulfurtransferase
C4B63_31g105	signal peptidase type I
C4B63_32g205	notchless homolog
C4B63_32g217	2-amino-3-ketobutyrate coenzyme A ligase
C4B63_32g22	Uncharacterized WD repeat-containing protein C17D11.16
C4B63_34g107	Nop14-like family
C4B63_34g1460c	mucin TcSMUGL
C4B63_34g287	glycerate kinase
C4B63_34g288	glycerate kinase
C4B63_34g289	glycerate kinase
C4B63_34g290	glycerate kinase
C4B63_35g189	eukaryotic translation initiation factor 1A
C4B63_360g21	unspecified product
C4B63_38g144	mitochondrial carrier protein
C4B63_38g320c	conserved hypothetical protein
C4B63_39g333	eukaryotic translation initiation factor 3 subunit 2
C4B63_3g1120	conserved hypothetical protein
C4B63_41g251	arginine N-methyltransferase
C4B63_42g103	pyridoxal kinase
C4B63_42g178	histidyl-tRNA synthetase
C4B63_42g220	transmembrane/endomembrane-like protein
C4B63_42g251	conserved hypothetical protein
C4B63_42g63	adenosine kinase
C4B63_42g70	p22 protein precursor
C4B63_43g125	centromere/microtubule binding protein cbf5
C4B63_43g161	ATP-dependent RNA helicase SUB2
C4B63_44g215	tRNA:m(4)X modification enzyme TRM13
C4B63_44g246	nucleolar protein
C4B63_45g126	RNA recognition motif (a.k.a. RRM, RBD, or RNP domain)
C4B63_45g239	lysyl-tRNA synthetase
C4B63_46g11	chaperonin TCP20
C4B63_46g91	leucyl-tRNA synthetase
C4B63_47g52	deoxyhypusine synthase
C4B63_47g53	deoxyhypusine synthase
C4B63_48g132	universal minicircle sequence binding protein
C4B63_48g168	AAA ATPase
C4B63_48g25	conserved hypothetical protein
C4B63_48g85	chaperonin HSP60, mitochondrial precursor
C4B63_48g86	chaperonin HSP60, mitochondrial precursor
C4B63_48g87	chaperonin HSP60, mitochondrial precursor
C4B63_48g88	chaperonin HSP60, mitochondrial precursor
C4B63_48g89	chaperonin HSP60, mitochondrial precursor
C4B63_48g90	chaperonin HSP60, mitochondrial precursor
C4B63_48g91	chaperonin HSP60, mitochondrial precursor
C4B63_48g92	chaperonin HSP60, mitochondrial precursor
C4B63_4g131	mitochondrial carrier protein
C4B63_4g200	conserved hypothetical protein
C4B63_4g201	Protein ILITYHIA
C4B63_4g267	mitochondrial carrier protein
C4B63_4g357	Activator of 90 kDa heat shock protein ATPase homolog 1
C4B63_4g453	conserved hypothetical protein
C4B63_4g454	nucleosome assembly protein
C4B63_4g64	Mitochondrial ADP/ATP carrier protein 5
C4B63_4g68	metallo-peptidase, Clan MG, Family M24
C4B63_4g77	conserved hypothetical protein
C4B63_4g79	ribosome biogenesis protein
C4B63_55g62	Ribosomal RNA small subunit methyltransferase NEP1
C4B63_57g88	fibrillarin
C4B63_58g135	heat shock protein DNAJ
C4B63_58g143	Lupus La protein homolog
C4B63_59g115	stress-inducible protein STI1-like(fragment)
C4B63_60g172	conserved hypothetical protein
C4B63_63g12	protein disulfide isomerase 2
C4B63_63g15	ribosomal protein L1-like protein
C4B63_63g16	chaperonin alpha subunit
C4B63_63g35	histidine ammonia-lyase
C4B63_63g58	Pre-rRNA-processing protein esf1
C4B63_69g26	endo-beta-N-acetylglucosaminidase
C4B63_6g118	Mitochondrial import receptor subunit ATOM46
C4B63_6g119	oxidoreductase-like protein
C4B63_6g129	heat shock protein 70 (hsp70)
C4B63_6g130	heat shock protein 70 (hsp70)(fragment)
C4B63_6g132	heat shock protein 70 (hsp70)
C4B63_6g136	heat shock protein 70 (hsp70)
C4B63_6g238	cation transporter
C4B63_6g239	cation transporter
C4B63_6g240	cation transporter
C4B63_6g241	cation transporter
C4B63_6g242	cation transporter
C4B63_6g243	cation transporter
C4B63_6g245	cation transporter
C4B63_6g246	cation transporter
C4B63_6g247	cation transporter
C4B63_6g248	cation transporter
C4B63_6g297	conserved hypothetical protein
C4B63_6g305	heat shock protein 70 (hsp70)
C4B63_6g306	heat shock protein 70 (hsp70)(fragment)
C4B63_6g307	heat shock protein 70 (hsp70)(fragment)
C4B63_6g308	heat shock protein 70 (hsp70)
C4B63_6g309	heat shock protein 70 (hsp70)
C4B63_6g310	heat shock protein 70 (hsp70)(fragment)
C4B63_6g312	heat shock protein 70 (hsp70)
C4B63_6g313	heat shock protein 70 (hsp70)(fragment)
C4B63_6g314	heat shock protein 70 (hsp70)
C4B63_6g315	heat shock protein 70 (hsp70)
C4B63_6g316	heat shock protein 70 (hsp70)
C4B63_6g317	heat shock protein 70 (hsp70)
C4B63_6g403	palmitoyl acyltransferase 4
C4B63_6g404	Ribosome biogenesis protein bms1
C4B63_6g405	Ribosome biogenesis protein BMS1 homolog
C4B63_6g489	Something about silencing protein 10
C4B63_6g495	Ankyrin repeat-containing protein P16F5.05c
C4B63_6g516	WD repeat-containing protein 75
C4B63_6g550	NAD(P)-dependent steroid dehydrogenase protein
C4B63_6g556	NAD(P)-dependent steroid dehydrogenase protein
C4B63_70g129	metallo-peptidase, Clan MF, Family M17
C4B63_70g61	RNA recognition motif. (a.k.a. RRM, RBD, or RNP domain)
C4B63_70g78	PA domain containing protein
C4B63_72g114	conserved hypothetical protein
C4B63_78g20	nucleolar RNA-binding protein
C4B63_78g23	nucleolar RNA-binding protein
C4B63_78g25	nucleolar RNA-binding protein
C4B63_79g54	hypoxanthine-guanine phosphoribosyltransferase
C4B63_7g210	Ribonuclease P protein subunit p25-like protein
C4B63_7g212	ruvB-like DNA helicase
C4B63_7g223	eukaryotic translation initiation factor 3 subunit g
C4B63_7g400	ER membrane protein complex subunit 1
C4B63_81g100	chaperonin HSP60, mitochondrial precursor
C4B63_81g101	chaperonin HSP60, mitochondrial precursor
C4B63_81g102	chaperonin HSP60, mitochondrial precursor
C4B63_81g103	chaperonin HSP60, mitochondrial precursor
C4B63_81g104	chaperonin HSP60, mitochondrial precursor
C4B63_81g105	chaperonin HSP60, mitochondrial precursor
C4B63_81g106	chaperonin HSP60, mitochondrial precursor
C4B63_81g40	ATP-dependent DEAD/H RNA helicase
C4B63_83g36	ppnN
C4B63_83g73	ribonucleoprotein p18, mitochondrial precursor
C4B63_83g80	cytoplasmic l-asparaginase i-like protein
C4B63_84g21	conserved hypothetical protein
C4B63_84g87	heat shock protein 85
C4B63_84g88	heat shock protein 85
C4B63_84g89	heat shock protein 85
C4B63_84g90	heat shock protein 85(fragment)
C4B63_8g2656c	10 kDa heat shock protein
C4B63_8g467	NMD3 family
C4B63_8g559	Adenine phosphoribosyltransferase
C4B63_93g64	amidohydrolase
C4B63_94g51	Mucin-1
C4B63_97g18	heat shock protein 110
C4B63_9g246	FtsJ cell division protein
C4B63_9g263	trypanothione synthetase
C4B63_9g396	sedoheptulose-1,7-bisphosphatase
C4B63_9g453	conserved hypothetical protein
C4B63_9g484	5-methylcytosine rRNA methyltransferase NSUN4

Hemin, Hb and No heme common DEGs (t24 vs. t0)

Downregulated

ID	Annotation
C4B63_101g12	sterol 24-c-methyltransferase
C4B63_109g41	Trypomastigote, Alanine, Serine and Valine rich protein (TASV), subfamily C
C4B63_109g61	trans-sialidase, Group II
C4B63_112g40	surface protease GP63
C4B63_112g66	Gamma carbonic anhydrase 2, mitochondrial
C4B63_113g41	thiol-dependent reductase 1
C4B63_113g42	thiol-dependent reductase 1
C4B63_129g44	cytochrome c oxidase VIII (COX VIII)
C4B63_12g242	SKP1-like protein
C4B63_130g39	trans-sialidase, Group II
C4B63_137g16	cytochrome c oxidase assembly protein
C4B63_140g32	cytochrome c oxidase VIII (COX VIII)
C4B63_14g1	hexose transporter
C4B63_14g2	hexose transporter(fragment)
C4B63_155g10	trans-sialidase
C4B63_156g18	trans-sialidase
C4B63_156g6	trans-sialidase
C4B63_163g33	protein disulfide isomerase
C4B63_16g136	conserved hypothetical protein
C4B63_170g37	conserved hypothetical protein
C4B63_170g5	conserved hypothetical protein
C4B63_17g48	ubiquitin-conjugating enzyme E2
C4B63_18g271	conserved hypothetical protein
C4B63_196g32	Mucin-associated surface protein (MASP)
C4B63_19g86	CYC2-like cyclin 6
C4B63_1g94	trans-sialidase, Group II
C4B63_1g953	trans-sialidase, Group V
C4B63_21g10	arginine permease
C4B63_21g13	arginine permease
C4B63_21g14	arginine permease
C4B63_21g20	arginine permease
C4B63_21g21	arginine permease
C4B63_21g25	arginine permease
C4B63_21g33	small GTP-binding protein Rab7
C4B63_21g7	arginine permease
C4B63_21g9	arginine permease
C4B63_228g46	glutamamyl carboxypeptidase
C4B63_228g47	glutamamyl carboxypeptidase
C4B63_228g48	glutamamyl carboxypeptidase
C4B63_228g49	glutamamyl carboxypeptidase
C4B63_228g50	glutamamyl carboxypeptidase
C4B63_228g51	glutamamyl carboxypeptidase(fragment)
C4B63_228g52	glutamamyl carboxypeptidase(fragment)
C4B63_22g167	calpain-like cysteine peptidase
C4B63_22g743c	calpain-like cysteine peptidase
C4B63_232g21	trans-sialidase, Group I
C4B63_23g17	Battenin
C4B63_244g9	trans-sialidase, Group I
C4B63_248g14	glucose-6-phosphate isomerase, glycosomal
C4B63_24g165	protein kinase
C4B63_24g199	conserved hypothetical protein
C4B63_259g12	conserved hypothetical protein
C4B63_25g29	trans-sialidase, Group I
C4B63_25g331	trans-sialidase, Group I
C4B63_260g20	trans-sialidase, Group II
C4B63_26g311	ABC transporter(fragment)
C4B63_277g1	glutamamyl carboxypeptidase(fragment)
C4B63_277g10	glutamamyl carboxypeptidase
C4B63_277g12	glutamamyl carboxypeptidase
C4B63_277g13	glutamamyl carboxypeptidase
C4B63_277g14	glutamamyl carboxypeptidase
C4B63_277g15	glutamamyl carboxypeptidase
C4B63_277g16	glutamamyl carboxypeptidase
C4B63_277g17	glutamamyl carboxypeptidase
C4B63_277g18	glutamamyl carboxypeptidase(fragment)
C4B63_277g2	glutamamyl carboxypeptidase
C4B63_277g20	glutamamyl carboxypeptidase(fragment)
C4B63_277g21	glutamamyl carboxypeptidase
C4B63_277g4	glutamamyl carboxypeptidase
C4B63_277g5	glutamamyl carboxypeptidase
C4B63_277g6	glutamamyl carboxypeptidase
C4B63_277g8	glutamamyl carboxypeptidase
C4B63_277g9	glutamamyl carboxypeptidase
C4B63_27g185	conserved hypothetical protein
C4B63_294g1	glutamamyl carboxypeptidase
C4B63_294g10	glutamamyl carboxypeptidase
C4B63_294g11	glutamamyl carboxypeptidase
C4B63_294g12	glutamamyl carboxypeptidase
C4B63_294g13	glutamamyl carboxypeptidase
C4B63_294g2	glutamamyl carboxypeptidase(fragment)
C4B63_294g5	glutamamyl carboxypeptidase
C4B63_294g6	glutamamyl carboxypeptidase
C4B63_294g7	glutamamyl carboxypeptidase
C4B63_294g8	glutamamyl carboxypeptidase
C4B63_294g9	glutamamyl carboxypeptidase
C4B63_29g223	trans-sialidase, Group II
C4B63_2g547	receptor-type adenylate cyclase
C4B63_2g548	receptor-type adenylate cyclase
C4B63_2g549	receptor-type adenylate cyclase
C4B63_30g122	surface protease GP63
C4B63_30g150	cystinosin
C4B63_30g275	protein disulfide isomerase
C4B63_312g7	conserved hypothetical protein
C4B63_31g119	trans-sialidase, Group II
C4B63_322g5	glutamamyl carboxypeptidase
C4B63_336g2	glutamamyl carboxypeptidase(fragment)
C4B63_336g3	metallo-peptidase, clan MH, family M18(fragment)
C4B63_336g4	glutamamyl carboxypeptidase
C4B63_336g5	glutamamyl carboxypeptidase
C4B63_336g6	glutamamyl carboxypeptidase
C4B63_33g163	unspecified product
C4B63_33g164	unspecified product
C4B63_33g167	trans-sialidase, Group II
C4B63_34g117	cytochrome b5-dependent oleate desaturase
C4B63_34g140	trans-sialidase, Group IV
C4B63_350g1	glutamamyl carboxypeptidase
C4B63_350g2	glutamamyl carboxypeptidase(fragment)
C4B63_350g4	metallo-peptidase, clan MH, family M18
C4B63_350g5	glutamamyl carboxypeptidase(fragment)
C4B63_37g382	unspecified product
C4B63_37g383	unspecified product
C4B63_38g178	UDP-Gal or UDP-GlcNAc-dependent glycosyltransferase
C4B63_38g180	UDP-Gal or UDP-GlcNAc-dependent glycosyltransferase
C4B63_38g184	UDP-Gal or UDP-GlcNAc-dependent glycosyltransferase
C4B63_39g346	nucleic acid binding protein
C4B63_39g66	trans-sialidase, Group V
C4B63_40g30	conserved hypothetical protein
C4B63_431g1	glutamamyl carboxypeptidase(fragment)
C4B63_431g3	glutamamyl carboxypeptidase
C4B63_431g4	glutamamyl carboxypeptidase
C4B63_431g5	glutamamyl carboxypeptidase
C4B63_431g6	glutamamyl carboxypeptidase
C4B63_431g7	glutamamyl carboxypeptidase
C4B63_431g8	glutamamyl carboxypeptidase
C4B63_431g9	glutamamyl carboxypeptidase
C4B63_43g225	conserved hypothetical protein
C4B63_43g83	trans-sialidase, Group II
C4B63_44g106	cytochrome c oxidase VIII (COX VIII)
C4B63_44g228	amino acid permease
C4B63_459g7	UDP-Gal or UDP-GlcNAc-dependent glycosyltransferase
C4B63_459g9	UDP-Gal or UDP-GlcNAc-dependent glycosyltransferase
C4B63_462g10	glutamamyl carboxypeptidase
C4B63_462g11	glutamamyl carboxypeptidase
C4B63_462g12	glutamamyl carboxypeptidase
C4B63_462g13	glutamamyl carboxypeptidase
C4B63_46g58	conserved hypothetical protein
C4B63_46g62	conserved hypothetical protein
C4B63_47g128	trans-sialidase
C4B63_47g75	splicing factor ptsr1 interacting protein
C4B63_485g5	glutamamyl carboxypeptidase(fragment)
C4B63_4g496	transporter
C4B63_550g1	metallo-peptidase, clan MH, family M18
C4B63_550g3	metallo-peptidase, clan MH, family M18
C4B63_550g6	glutamamyl carboxypeptidase
C4B63_5g44	trans-sialidase, Group V
C4B63_63g102	hexose transporter
C4B63_63g104	hexose transporter
C4B63_63g106	hexose transporter
C4B63_63g108	hexose transporter
C4B63_63g110	hexose transporter
C4B63_68g134	trans-sialidase, Group II
C4B63_7g143	Tubulin polymerization-promoting protein family member 2
C4B63_87g8	glutamamyl carboxypeptidase
C4B63_93g25	unspecified product
C4B63_98g22	trans-sialidase
C4B63_98g34	trans-sialidase
C4B63_9g382	conserved hypothetical protein

Upregulated

ID	Annotation
C4B63_101g36	inositol-3-phosphate synthase
C4B63_10g406	High mobility group protein TDP1
C4B63_10g492	conserved hypothetical protein
C4B63_10g517	tryptophanyl-tRNA synthetase
C4B63_112g84c	Histone H2B variant V
C4B63_113g22	heat shock protein 85(fragment)
C4B63_113g23	heat shock protein 85(fragment)
C4B63_113g24	heat shock protein 85(fragment)
C4B63_113g25	heat shock protein 85
C4B63_113g26	heat shock protein 85
C4B63_113g28	heat shock protein 85
C4B63_113g29	heat shock protein 85
C4B63_113g30	heat shock protein 85
C4B63_113g31	heat shock protein 85(fragment)
C4B63_113g32	heat shock protein 85
C4B63_113g33	heat shock protein 85
C4B63_11g29	2,4-dihydroxyhept-2-ene-1,7-dioic acid aldolase
C4B63_11g36	kinesin
C4B63_11g37	kinesin
C4B63_11g8	succinyl-CoA synthetase alpha subunit
C4B63_127g35	peptidase
C4B63_127g54	squalene synthase
C4B63_128g301c	histone H4
C4B63_128g305c	histone H4
C4B63_128g308c	histone H4
C4B63_128g316c	histone H4
C4B63_12g153	metallo-peptidase, Clan MF, Family M17
C4B63_12g237	eukaryotic release factor 3
C4B63_12g295	GTP-binding nuclear protein rtb2
C4B63_133g56	heat shock protein 70 (hsp70)
C4B63_133g57	heat shock protein 70 (hsp70)
C4B63_133g58	heat shock protein 70 (hsp70)
C4B63_133g59	heat shock protein 70 (hsp70)
C4B63_133g61	heat shock protein 70 (hsp70)
C4B63_133g62	heat shock protein 70 (hsp70)(fragment)
C4B63_133g63	heat shock protein 70 (hsp70)
C4B63_137g34	heat shock protein 70 (hsp70)(fragment)
C4B63_137g37	heat shock protein 70 (hsp70)(fragment)
C4B63_137g41	heat shock protein 70 (hsp70)
C4B63_13g145	eukaryotic translation initiation factor 3 subunit l
C4B63_13g263	GPI alpha-mannosyltransferase III
C4B63_13g34	21 kDa cyclophilin
C4B63_142g5	conserved hypothetical protein
C4B63_147g41	conserved hypothetical protein
C4B63_149g20	cyclophilin
C4B63_14g165	N-lysine methyltransferase SETD6
C4B63_14g220c	kinetoplast DNA-associated protein
C4B63_14g241	trypanothione reductase
C4B63_14g249	structural maintenance of chromosome 3
C4B63_14g62	kinetoplast DNA-associated protein
C4B63_14g96	isoleucil-tRNA synthetase
C4B63_14g97	isoleucyl-tRNA synthetase
C4B63_157g28	FK506-binding protein (FKBP)-type peptidyl-prolyl isomerase
C4B63_15g14	oligosaccharyl transferase subunit
C4B63_160g6	poly(A)-specific ribonuclease PARN
C4B63_161g18	UDP-Gal or UDP-GlcNAc-dependent glycosyltransferase
C4B63_162g33	chaperonin TCP20
C4B63_168g33	kinesin
C4B63_16g315	mitochondrial DNA primase
C4B63_16g326	conserved hypothetical protein
C4B63_171g30	FK506-binding protein (FKBP)-type peptidyl-prolyl isomerase
C4B63_175g9	heat shock protein DNAJ
C4B63_17g102	conserved hypothetical protein
C4B63_17g179	Importin subunit beta-3
C4B63_17g268	PSP1 C-terminal conserved region
C4B63_17g90	T-complex protein 1, beta subunit
C4B63_180g2	conserved hypothetical protein
C4B63_180g3	conserved hypothetical protein
C4B63_180g4	conserved hypothetical protein
C4B63_184g15	conserved hypothetical protein
C4B63_188g129c	histone H4
C4B63_188g130c	histone H4
C4B63_188g132c	histone H4
C4B63_188g135c	histone H4
C4B63_188g137c	histone H4
C4B63_188g138c	histone H4
C4B63_188g140c	histone H4
C4B63_188g141c	histone H4
C4B63_188g144c	histone H4
C4B63_188g145c	histone H4
C4B63_188g146c	histone H4
C4B63_188g147c	histone H4
C4B63_188g148c	histone H4
C4B63_188g149c	histone H4
C4B63_188g152c	histone H4
C4B63_188g153c	histone H4
C4B63_188g154c	histone H4
C4B63_188g157c	histone H4
C4B63_188g159c	histone H4
C4B63_188g160c	histone H4
C4B63_188g161c	histone H4
C4B63_188g162c	histone H4
C4B63_188g163c	histone H4
C4B63_188g164c	histone H4
C4B63_18g164	S-adenosylmethionine synthetase
C4B63_18g19	ESAG8-associated protein
C4B63_18g204	3-hydroxy-3-methylglutaryl-CoA reductase
C4B63_18g212	valyl-tRNA synthetase
C4B63_18g229	conserved hypothetical protein
C4B63_18g260	small glutamine-rich tetratricopeptide repeat protein
C4B63_18g287	heat shock 70 kDa protein, mitochondrial precursor
C4B63_18g292	conserved hypothetical protein
C4B63_18g293	heat shock 70 kDa protein, mitochondrial precursor
C4B63_192g3	AAA ATPase
C4B63_193g9	metallo-peptidase, Clan MF, Family M17
C4B63_194g18	Histone-lysine N-methyltransferase, H3 lysine-76 specific
C4B63_196g51	CDP-diacylglycerol--inositol 3-phosphatidyltransferase
C4B63_19g195	Nuclear pore complex protein 158
C4B63_207g13	prostaglandin F synthase
C4B63_207g15	prostaglandin F synthase
C4B63_208g38	kinetoplastid kinetochore protein 8
C4B63_20g213	enoyl-CoA hydratase/isomerase family protein
C4B63_20g219	kinetoplast-associated protein 3
C4B63_214g13	UDP-Gal or UDP-GlcNAc-dependent glycosyltransferase
C4B63_214g14	UDP-Gal or UDP-GlcNAc-dependent glycosyltransferase
C4B63_21g238	conserved hypothetical protein
C4B63_227g2	glucan 1,3-alpha-glucosidase
C4B63_22g175	pumilio/PUF RNA binding protein 9
C4B63_22g22	ARM-like helical domain-containing protein
C4B63_22g23	ARM-like helical domain-containing protein
C4B63_238g17	conserved hypothetical protein
C4B63_23g200	Flagellum attachment zone protein 19
C4B63_23g209	conserved hypothetical protein
C4B63_23g234	inosine-adenosine-guanosine-nucleosidehydrolase
C4B63_23g275	ATP-dependent DEAD/H RNA helicase
C4B63_23g83	Aminopeptidase M1
C4B63_24g298	CDP-diacylglycerol--inositol 3-phosphatidyltransferase
C4B63_24g325	conserved hypothetical protein
C4B63_252g22	T-complex protein 1, beta subunit
C4B63_25g294	Thioredoxin-like
C4B63_25g317	threonine synthase
C4B63_261g22c	conserved hypothetical protein
C4B63_26g204	phosphomevalonate kinase protein
C4B63_26g271	conserved hypothetical protein
C4B63_26g279	ABC transporter
C4B63_26g287	ABC transporter
C4B63_271g12	thymidine kinase
C4B63_276g16	conserved hypothetical protein
C4B63_28g106	malic enzyme
C4B63_28g107	malic enzyme
C4B63_28g64	aspartate aminotransferase
C4B63_297g10	polypyrimidine tract-binding protein
C4B63_2g235	Co-chaperone protein P23
C4B63_2g293	conserved hypothetical protein
C4B63_2g294	40 kDa cyclophilin
C4B63_2g412	R27-2 protein
C4B63_2g422	cell division cycle protein 45 (CDC45)
C4B63_2g445	Metallocarboxypeptidase 1
C4B63_2g446	Metallocarboxypeptidase 1
C4B63_2g485	T-complex protein 1, beta subunit
C4B63_2g594	Snake venom metalloprotease inhibitor 02D01
C4B63_2g690	chaperonin alpha subunit
C4B63_2g829	conserved hypothetical protein
C4B63_302g42c	histone H3
C4B63_302g52c	histone H3
C4B63_302g56c	histone H3
C4B63_302g62c	histone H3
C4B63_302g64c	histone H3
C4B63_302g72c	histone H3
C4B63_302g78c	histone H3
C4B63_303g16	trypanothione reductase
C4B63_303g6	Histone H2B variant V
C4B63_303g7	Histone H2B variant V
C4B63_30g140	conserved hypothetical protein
C4B63_30g290	mercaptopyruvate sulfurtransferase
C4B63_30g940c	Histone H2B variant V
C4B63_32g214	2-amino-3-ketobutyrate coenzyme A ligase
C4B63_32g217	2-amino-3-ketobutyrate coenzyme A ligase
C4B63_32g225	3-hydroxy-3-methylglutaryl-CoA synthase
C4B63_32g326	cell division cycle protein
C4B63_340g1	cell division cycle protein
C4B63_34g288	glycerate kinase
C4B63_34g290	glycerate kinase
C4B63_355g19	inositol-3-phosphate synthase(fragment)
C4B63_37g366	thimet oligopeptidase
C4B63_38g320c	conserved hypothetical protein
C4B63_38g38	actin 3
C4B63_38g39	actin 3
C4B63_39g333	eukaryotic translation initiation factor 3 subunit 2
C4B63_41g271	conserved hypothetical protein
C4B63_42g178	histidyl-tRNA synthetase
C4B63_42g242	2-hydroxy-3-oxopropionate reductase
C4B63_42g251	conserved hypothetical protein
C4B63_42g63	adenosine kinase
C4B63_42g70	p22 protein precursor
C4B63_43g204	thymidine kinase
C4B63_43g3	kinesin
C4B63_44g192	mitochondrial RNA binding protein
C4B63_45g99	conserved hypothetical protein
C4B63_46g11	chaperonin TCP20
C4B63_46g91	leucyl-tRNA synthetase
C4B63_47g53	deoxyhypusine synthase
C4B63_48g101	kinetoplastid kinetochore protein 1
C4B63_48g85	chaperonin HSP60, mitochondrial precursor
C4B63_48g87	chaperonin HSP60, mitochondrial precursor
C4B63_4g199	ethanolamine phosphotransferase
C4B63_4g200	conserved hypothetical protein
C4B63_4g201	Protein ILITYHIA
C4B63_4g220	conserved hypothetical protein
C4B63_4g357	Activator of 90 kDa heat shock protein ATPase homolog 1
C4B63_4g375	glucan 1,3-alpha-glucosidase
C4B63_4g484	kinesin
C4B63_4g485	kinesin
C4B63_4g527	mitochondrial carrier protein
C4B63_4g558	phosphatidic acid phosphatase protein
C4B63_4g64	Mitochondrial ADP/ATP carrier protein 5
C4B63_4g68	metallo-peptidase, Clan MG, Family M24
C4B63_4g77	conserved hypothetical protein
C4B63_51g117	Uncharacterized protein PB7E8.02
C4B63_52g41	mitochondrial DNA polymerase I protein C
C4B63_55g28	kinesin
C4B63_55g29	kinesin
C4B63_59g115	stress-inducible protein STI1-like(fragment)
C4B63_61g127	histone H2A variant Z
C4B63_62g82	thimet oligopeptidase
C4B63_63g16	chaperonin alpha subunit
C4B63_63g35	histidine ammonia-lyase
C4B63_63g46	2,3-bisphosphoglycerate-independent phosphoglycerate mutase
C4B63_63g91	GTPase activating protein
C4B63_69g25	DNA excision/repair protein SNF2
C4B63_69g26	endo-beta-N-acetylglucosaminidase
C4B63_6g113	ribonucleoside-diphosphate reductase large chain
C4B63_6g2482c	histone H3
C4B63_6g2496c	histone H3
C4B63_6g2498c	histone H3
C4B63_6g2502c	histone H3
C4B63_6g2504c	histone H3
C4B63_6g2508c	histone H3
C4B63_6g2516c	histone H3
C4B63_6g2520c	histone H3
C4B63_6g2522c	histone H3
C4B63_6g2524c	histone H3
C4B63_6g2526c	histone H3
C4B63_6g2530c	histone H3
C4B63_6g2546c	histone H3
C4B63_6g2548c	histone H3
C4B63_6g2556c	histone H3
C4B63_6g2558c	histone H3
C4B63_6g2564c	histone H3
C4B63_6g2566c	histone H3
C4B63_6g307	heat shock protein 70 (hsp70)(fragment)
C4B63_6g308	heat shock protein 70 (hsp70)
C4B63_6g310	heat shock protein 70 (hsp70)(fragment)
C4B63_6g312	heat shock protein 70 (hsp70)
C4B63_6g313	heat shock protein 70 (hsp70)(fragment)
C4B63_6g315	heat shock protein 70 (hsp70)
C4B63_6g509	protein kinase
C4B63_6g532	Putative trans-acting enoyl reductase MT2525
C4B63_6g578	histone H3
C4B63_70g129	metallo-peptidase, Clan MF, Family M17
C4B63_70g94	PIF1 helicase-like protein
C4B63_76g31	conserved hypothetical protein,zinc finger protein family member
C4B63_78g20	nucleolar RNA-binding protein
C4B63_78g25	nucleolar RNA-binding protein
C4B63_79g54	hypoxanthine-guanine phosphoribosyltransferase
C4B63_7g103	conserved hypothetical protein
C4B63_7g210	Ribonuclease P protein subunit p25-like protein
C4B63_7g400	ER membrane protein complex subunit 1
C4B63_81g101	chaperonin HSP60, mitochondrial precursor
C4B63_81g104	chaperonin HSP60, mitochondrial precursor
C4B63_81g105	chaperonin HSP60, mitochondrial precursor
C4B63_81g19	chaperonin HSP60, mitochondrial precursor
C4B63_81g40	ATP-dependent DEAD/H RNA helicase
C4B63_83g73	ribonucleoprotein p18, mitochondrial precursor
C4B63_84g21	conserved hypothetical protein
C4B63_84g22	conserved hypothetical protein
C4B63_84g87	heat shock protein 85
C4B63_84g88	heat shock protein 85
C4B63_84g89	heat shock protein 85
C4B63_84g90	heat shock protein 85(fragment)
C4B63_85g60	tricarboxylate carrier
C4B63_87g42	conserved hypothetical protein
C4B63_8g196	DNA ligase
C4B63_8g2646c	10 kDa heat shock protein
C4B63_8g2656c	10 kDa heat shock protein
C4B63_8g483	asparagine synthetase a
C4B63_8g559	Adenine phosphoribosyltransferase
C4B63_94g51	Mucin-1
C4B63_97g18	heat shock protein 110
C4B63_97g35	P-type H+-ATPase
C4B63_9g209	conserved hypothetical protein
C4B63_9g263	trypanothione synthetase
C4B63_9g516	heat shock protein Hsp20
C4B63_9g524	eukaryotic translation initiation factor 3 subunit k

Gene Ontology analysis of Hemin, Hb and No heme common DEGs (downregulated, t4 vs. t0)										
	**BIOLOGICAL PROCESS**									
ID	Name	Bgd count	Result count	Result gene list	Pct of bgd	Fold enrichm	Odds ratio	P-value	Benjamini	Bonferroni
GO:0006525	arginine metabolic process	38	36	C4B63_228g46,C	94.7	58.39	1472.5	1,88E-64	1,68E-62	3,36E-62
GO:0009064	glutamine family amino acid metabolic	55	36	C4B63_228g46,C	65.5	40.34	154.69	6,14E-53	2,75E-51	1,10E-50
GO:0006599	phosphagen metabolic process	3	3	C4B63_26g278,C	100.0	61.63	inf	4,18E-06	3,25E-05	0.000748399141299
GO:0016559	peroxisome fission	6	3	C4B63_2g702,C4	50.0	30.82	61.95	8,07E-05	0.000577595	0.0144398783747
GO:0046386	deoxyribose phosphate catabolic proc	2	2	C4B63_163g21,C	100.0	61.63	inf	0.000261388	0.001671021	0.0467886032493
	**CELLULAR COMPONENT**									
ID	Name	Bgd count	Result count	Result gene list	Pct of bgd	Fold enrichm	Odds ratio	P-value	Benjamini	Bonferroni
GO:0005777	peroxisome	19	3	C4B63_2g702,C4	15.8	9.73	11.6	0.003348108	0.016182523	0.0970951413332
	**MOLECULAR FUNCTION**									
ID	Name	Bgd count	Result count	Result gene list	Pct of bgd	Fold enrichm	Odds ratio	P-value	Benjamini	Bonferroni
GO:0004180	carboxypeptidase activity	94	64	C4B63_228g46,C	68.1	41.96	238.88	2,43E-98	1,99E-96	1,99E-96
GO:0016787	hydrolase activity	1860	78	C4B63_112g40,C	4.2	2.58	4.77	5,74E-19	7,85E-18	4,71E-17
GO:0016775	phosphotransferase activity, nitrogenc	3	3	C4B63_26g278,C	100.0	61.63	inf	4,18E-06	3,81E-05	0.000342842064729

Gene Ontology analysis of Hemin, Hb and No heme common DEGs (upregulated, t4 vs. t0)										
	**BIOLOGICAL PROCESS**									
ID	Name	Bgd count	Result count	Result gene list	Pct of bgd	Fold enrichm	Odds ratio	P-value	Benjamini	Bonferroni
GO:0006457	protein folding	91	49	C4B63_104g78,C	53.8	20.14	53.77	2,47E-54	9,39E-52	9,39E-52
GO:0030001	metal ion transport	36	10	C4B63_6g238,C4	27.8	10.39	14.6	2,12E-08	2,01E-06	8,04E-06
GO:0042254	ribosome biogenesis	76	12	C4B63_123g35,C	15.8	5.91	7.15	6,83E-07	3,71E-05	0.000259405458758
	**CELLULAR COMPONENT**									
ID	Name	Bgd count	Result count	Result gene list	Pct of bgd	Fold enrichm	Odds ratio	P-value	Benjamini	Bonferroni
GO:0005737	cytoplasm	813	50	C4B63_104g78,C	6.2	2.3	2.77	1,16E-08	1,85E-07	9,26E-07
GO:0005788	endoplasmic reticulum lumen	4	3	C4B63_104g78,C	75.0	28.06	110.67	7,39E-05	0.000591466	0.00591466477824
GO:0031981	nuclear lumen	94	10	C4B63_123g35,C	10.6	3.98	4.49	0.000193793	0.001354101	0.0155034768978
GO:0101031	chaperone complex	2	2	C4B63_252g22,C	100.0	37.41	inf	0.000711481	0.003954588	0.0569185043213
GO:0005739	mitochondrion	131	11	C4B63_11g8,C4	8.4	3.14	3.46	0.000741485	0.003954588	0.0593188206697
	**MOLECULAR FUNCTION**									
ID	Name	Bgd count	Result count	Result gene list	Pct of bgd	Fold enrichm	Odds ratio	P-value	Benjamini	Bonferroni
GO:0005524	ATP binding	1110	96	C4B63_10g439,C	8.6	3.24	5.21	1,65E-28	1,90E-26	3,96E-26
GO:0043167	ion binding	2086	113	C4B63_104g78,C	5.4	2.03	3.14	9,35E-17	1,40E-15	2,24E-14
GO:0008887	glycerate kinase activity	4	4	C4B63_34g287,C	100.0	37.41	inf	4,98E-07	6,29E-06	0.000119427682399

Gene Ontology analysis of Hemin, Hb and No heme common DEGs (downregulated, t24 vs. t0)										
	**BIOLOGICAL PROCESS**									
ID	Name	Bgd count	Result count	Result gene list	Pct of bgd	Fold enrichm	Odds ratio	P-value	Benjamini	Bonferroni
GO:0006525	arginine metabolic process	38	33	C4B63_228g46,C	86.8	54.7	539.92	3,06E-56	2,36E-54	4,72E-54
GO:0009064	glutamine family amino acid metabolic	55	33	C4B63_228g46,C	60.0	37.8	122.46	6,49E-47	2,50E-45	1,00E-44
GO:1901564	organonitrogen compound metabolic p	1615	49	C4B63_112g40,C	3.0	1.91	2.47	1,52E-06	1,17E-05	0.000234481029763
	**CELLULAR COMPONENT**									
ID	Name	Bgd count	Result count	Result gene list	Pct of bgd	Fold enrichm	Odds ratio	P-value	Benjamini	Bonferroni
GO:0016021	integral component of membrane	2487	51	C4B63_112g40,C	2.1	1.29	1.48	0.019632002	0.186504025	0.373008051728
	**MOLECULAR FUNCTION**									
ID	Name	Bgd count	Result count	Result gene list	Pct of bgd	Fold enrichm	Odds ratio	P-value	Benjamini	Bonferroni
GO:0004180	carboxypeptidase activity	94	58	C4B63_228g46,C	61.7	38.87	173.4	2,77E-85	1,85E-83	1,85E-83
GO:0016997	alpha-sialidase activity	711	23	C4B63_109g61,C	3.2	2.04	2.29	0.000748563	0.005572642	0.0501537786633

Gene Ontology analysis of Hemin, Hb and No heme common DEGs (upregulated, t24 vs. t0)										
	**BIOLOGICAL PROCESS**									
ID	Name	Bgd count	Result count	Result gene list	Pct of bgd	Fold enrichm	Odds ratio	P-value	Benjamini	Bonferroni
GO:0006457	protein folding	91	36	C4B63_113g22,C	39.6	14.86	28.24	9,88E-34	3,65E-31	3,65E-31
	**CELLULAR COMPONENT**									
ID	Name	Bgd count	Result count	Result gene list	Pct of bgd	Fold enrichm	Odds ratio	P-value	Benjamini	Bonferroni
GO:0005694	chromosome	168	60	C4B63_112g84c,	35.7	13.42	27.22	2,09E-53	2,80E-52	1,40E-51
GO:0005634	nucleus	424	40	C4B63_10g406,C	9.4	3.54	4.41	1,33E-12	8,11E-12	8,92E-11
GO:0005737	cytoplasm	813	38	C4B63_113g25,C	4.7	1.76	1.95	0.000388189	0.001529922	0.0260086900955
GO:0101031	chaperone complex	2	2	C4B63_252g22,C	100.0	37.57	inf	0.000705267	0.002486995	0.0472529220645
	**MOLECULAR FUNCTION**									
ID	Name	Bgd count	Result count	Result gene list	Pct of bgd	Fold enrichm	Odds ratio	P-value	Benjamini	Bonferroni
GO:0005515	protein binding	1216	104	C4B63_112g84c,	8.6	3.21	5.45	3,47E-31	1,34E-29	8,05E-29
GO:0005524	ATP binding	1110	73	C4B63_10g517,C	6.6	2.47	3.32	2,71E-14	5,71E-13	6,28E-12
GO:0043167	ion binding	2086	91	C4B63_10g517,C	4.4	1.64	2.11	1,01E-07	1,02E-06	2,34E-05
GO:0008235	metalloexopeptidase activity	16	5	C4B63_12g153,C	31.3	11.74	16.98	4,39E-05	0.000424503	0.0101880839521

Table S2. List and Gene Ontology analysis of DEGs common to hemoglobin, and no heme conditions in the t_4 vs. t_0 and t_{24} vs. t_0 analysis.

Table S3

Hemin and Hb common DEGs (t4 vs t0)

Downregulated genes

ID	Annotation	Hemin log2FC	Hemin padj	Hb log2FC	Hb padj
C4B63_18g39	CRAL/TRIO, N-terminal domain/CRAL/TRIO domain	-1,808774747	1,21E-18	-1,5460513	1,24E-13
C4B63_49g168	conserved hypothetical protein	-1,171980321	0,00057483	-0,80297	0,020838019
C4B63_27g1020c	RNA-binding protein	-0,994646682	0,00021044	-1,0598988	0,0001686
C4B63_43g225	conserved hypothetical protein	-0,815397296	0,01641791	-1,0107327	0,001827284
C4B63_24g199	conserved hypothetical protein	-0,808096617	0,00261519	-0,9946761	0,000288074
C4B63_22g780c	calpain-like cysteine peptidase	-0,792715519	0,00349498	-0,7770126	0,024259522
C4B63_14g158	serine/threonine kinase	-0,789239268	0,00070193	-0,8141541	0,006764196
C4B63_13g223	zinc-finger protein family member	-0,783410929	0,01104509	-0,8159978	0,021248307
C4B63_2g547	receptor-type adenylate cyclase	-1,021736703	0,00000206	-0,6383037	0,009906963
C4B63_2g548	receptor-type adenylate cyclase	-1,01278119	6,44E-07	-0,6685913	0,019117321
C4B63_24g313	protein kinase	-0,892294129	0,00438955	-0,6949108	0,048874265
C4B63_156g18	trans-sialidase	-0,820056863	0,00128107	-0,5831029	0,023175727
C4B63_2g309	NIMA-related kinase	-0,73843064	0,00321431	-0,6542999	0,018612444
C4B63_14g101	conserved hypothetical protein	-0,73001501	0,00711696	-0,5522618	0,044157687
C4B63_47g128	trans-sialidase	-0,722077618	0,00449876	-0,6172614	0,030082599
C4B63_37g368	Proline dehydrogenase, mitochondrial	-0,719624519	0,00038991	-0,6562838	0,004168137
C4B63_17g61	conserved hypothetical protein	-0,712900888	0,0030949	-0,663958	0,006473053
C4B63_55g86	peptidyl-prolyl cis-trans isomerase	-0,692982205	0,00101689	-0,5493152	0,030677799
C4B63_130g39	trans-sialidase, Group II	-0,692761653	0,00380615	-0,6601727	0,004984789
C4B63_156g6	trans-sialidase	-0,692091407	0,00961706	-0,634388	0,021248307
C4B63_19g141	electron transfer flavoprotein	-0,681873252	0,00017366	-0,6525703	0,000156788
C4B63_30g222	conserved hypothetical protein	-0,675207601	9,35E-07	-0,4475514	0,003576183
C4B63_52g153	conserved hypothetical protein	-0,67396387	0,01384785	-0,6660266	0,030701785
C4B63_8g495	Regulator of chromosome condensation (RCC1) repe	-0,672881236	0,00028299	-0,5601292	0,025263938
C4B63_82g448c	conserved hypothetical protein	-0,648960027	0,00123418	-0,6228363	0,005429819
C4B63_4g511	zinc-finger protein	-0,643850614	0,00185179	-0,5390746	0,033548808
C4B63_134g54	trans-sialidase, Group V	-0,642876138	0,00183035	-0,4698703	0,032182865
C4B63_134g49	trans-sialidase, Group V	-0,623373149	0,00673586	-0,4752388	0,020329456
C4B63_157g36	trans-sialidase, Group II	-0,620882028	0,03034162	-0,5483791	0,03207292
C4B63_9g387	conserved hypothetical protein	-0,618701592	0,00076891	-0,4105115	0,03207292
C4B63_10g494	thymine-7-hydroxylase	-0,614017026	0,00382912	-0,5652601	0,013757536
C4B63_9g448	superoxide dismutase	-0,607178566	0,00028299	-0,4963374	0,013034904
C4B63_354g1	electron transfer flavoprotein	-0,605761908	0,00586106	-0,6533645	0,005697135
C4B63_34g117	cytochrome b5-dependent oleate desaturase	-0,605479999	0,00460831	-0,5302052	0,04066761
C4B63_22g96	protein kinase	-0,60258246	0,01238382	-0,6543142	0,006359919
C4B63_9g445	conserved hypothetical protein	-0,599061375	0,02285797	-0,5586467	0,03518388
C4B63_42g100	calcium-binding protein	-0,598267766	0,00479273	-0,5089338	0,021645331
C4B63_192g2	amino acid permease 24	-0,597546613	0,00472846	-0,4979387	0,035045199
C4B63_23g17	Battenin	-0,59721217	0,00069049	-0,4584686	0,014625786
C4B63_193g27	protein associated with differentiation 4	-0,593366171	0,0328493	-0,6147894	0,021842808
C4B63_113g47	Generative cell specific 1 protein	-0,589224099	0,02400521	-0,6057219	0,021162993
C4B63_40g146	Gamma carbonic anhydrase 3, mitochondrial	-0,582182177	0,00639445	-0,636335	0,001944739
C4B63_21g33	small GTP-binding protein Rab7	-0,57016298	0,00054685	-0,6077617	0,0007303
C4B63_1g480	trans-sialidase, Group V	-0,568699562	0,00625476	-0,49473	0,019741116
C4B63_77g33	conserved hypothetical protein	-0,56505283	0,00422767	-0,5444212	0,005515167
C4B63_14g92	conserved hypothetical protein	-0,563674405	0,01765838	-0,6464864	0,007395037
C4B63_16g1800c	conserved hypothetical protein	-0,557125719	0,01412477	-0,6092664	0,004428084
C4B63_278g3	conserved hypothetical protein	-0,55609634	0,01218422	-0,5302308	0,023791923
C4B63_21g64	conserved hypothetical protein	-0,55449461	0,00453296	-0,5905586	0,023791923
C4B63_13g317	vesicle-associated membrane protein	-0,553362947	0,0017064	-0,506283	0,0071948
C4B63_63g52	conserved hypothetical protein	-0,543715991	0,01657612	-0,6062091	0,003597327
C4B63_31g61	dual-specificity protein phosphatase	-0,541297729	0,01511715	-0,5065708	0,044078245
C4B63_18g308	sphingosine 1-phosphate lyase	-0,521463368	0,00228593	-0,5420295	0,001740963
C4B63_21g27	arginine permease(fragment)	-0,519748902	0,04767094	-0,5205113	0,043951003
C4B63_21g112	conserved hypothetical protein	-0,516757842	0,01389474	-0,5847305	0,010158898
C4B63_45g82	conserved hypothetical protein	-0,514932996	0,00864866	-0,4458068	0,015174583
C4B63_459g4	conserved hypothetical protein	-0,513981065	0,01236402	-0,5382728	0,031620556
C4B63_4g505	ADP-ribosylation factor-like protein	-0,509282325	0,00381269	-0,4550279	0,021969701
C4B63_70g572c	cytochrome b5	-0,507647754	0,04832744	-0,5460632	0,025181875
C4B63_25g207	hydrolase-like protein	-0,506710476	0,02157845	-0,5497422	0,033160367
C4B63_4g180	serine/threonine protein kinase	-0,500578733	0,00026607	-0,5055941	0,000427798
C4B63_43g74	retrotransposon hot spot (RHS) protein	-0,498587976	0,01585039	-0,4907133	0,008901829
C4B63_4g143	conserved hypothetical protein	-0,498571243	0,03195312	-0,4970781	0,025865722
C4B63_43g134	cytochrome c oxidase subunit VI	-0,49686699	0,00464391	-0,5255918	0,016838085
C4B63_4g283	conserved hypothetical protein	-0,495554516	0,00335186	-0,6127837	0,000407948
C4B63_25g236	conserved hypothetical protein	-0,49363397	0,0303703	-0,4934296	0,041572205
C4B63_4g496	transporter	-0,492842334	0,00270345	-0,6362727	0,0000584
C4B63_173g6	Soluble NSF attachment protein, SNAP (paraflagelar	-0,488869117	0,00357026	-0,4648574	0,006068708
C4B63_21g25	arginine permease	-0,486813727	0,01297888	-0,5232119	0,007929772
C4B63_22g167	calpain-like cysteine peptidase	-0,485824991	0,00614085	-0,456573	0,01273885
C4B63_13g146	conserved hypothetical protein	-0,485593523	0,02543275	-0,4840782	0,040147495
C4B63_309g8	retrotransposon hot spot (RHS) protein	-0,478151858	0,03991761	-0,4812183	0,006338857
C4B63_47g59	conserved hypothetical protein	-0,476746018	0,02871472	-0,517646	0,018593091
C4B63_44g231	amino acid transporter	-0,47649774	0,03792391	-0,5205787	0,006338857
C4B63_60g198	retrotransposon hot spot (RHS) protein	-0,474491569	0,01729889	-0,482989	0,03222943
C4B63_43g174	Cytochrome b5 domain-containing protein 1	-0,473822678	0,00690065	-0,4484889	0,015893671
C4B63_2g156	protein kinase A catalytic subunit 3	-0,470032629	0,00365284	-0,4478492	0,033160367
C4B63_14g53	Basal body-orientation factor 1	-0,464910439	0,00818288	-0,5072042	0,003814788
C4B63_20g39	conserved hypothetical protein	-0,461761816	0,04195449	-0,5302818	0,004162844
C4B63_48g693c	conserved hypothetical protein	-0,46127976	0,01487736	-0,4874072	0,027698415
C4B63_16g104	conserved hypothetical protein	-0,460622112	0,04748063	-0,5088029	0,033289423
C4B63_175g11	Homologous-pairing protein 2 homolog	-0,44889857	0,03601283	-0,5557569	0,02478701
C4B63_44g106	cytochrome c oxidase VIII (COX VIII)	-0,441974089	0,0000861	-0,5109784	0,000986354
C4B63_3g1092	alanine racemase	-0,440397024	0,01480092	-0,4608152	0,006117138
C4B63_35g90	flagellar calcium-binding protein	-0,438905177	0,00941958	-0,4157322	0,013805357
C4B63_61g147	conserved hypothetical protein	-0,437378777	0,04490242	-0,5221927	0,016117594
C4B63_180g10	retrotransposon hot spot (RHS) protein	-0,436787792	0,02605464	-0,4231829	0,040794699
C4B63_30g247	glucosamine-fructose-6-phosphate aminotransferase	-0,429795757	0,01243976	-0,4355021	0,008059083
C4B63_27g185	conserved hypothetical protein	-0,421891782	0,03069738	-0,4147184	0,034520192
C4B63_32g242	Phosphorylated CTD interacting factor 1 WW domai	-0,421384575	0,00485976	-0,4469063	0,003637157
C4B63_99g112	alanine racemase	-0,415768718	0,0321732	-0,4197702	0,041403185
C4B63_2g712	galactokinase	-0,414605743	0,02205543	-0,5168173	0,004123273
C4B63_20g35	conserved hypothetical protein	-0,410970776	0,0209229	-0,4284884	0,022938251
C4B63_70g88	ATG16/SAP18/CVT11/APG16	-0,410957882	0,0093311	-0,4054939	0,016190048
C4B63_6g511	Hsp70-Hsp90 organizing protein 3	-0,409799709	0,02699253	-0,4855274	0,007159305
C4B63_18g322	conserved hypothetical protein	-0,409452141	0,01839807	-0,5189421	0,003282909
C4B63_13g310	NAD(P)-dependent oxidoreductase	-0,405850812	0,04192059	-0,4078745	0,038304207
C4B63_14g43	centrin-3	-0,404864269	0,04810396	-0,4338507	0,006932193
C4B63_17g91	Tyrosine-protein phosphatase DSP2	-0,403673477	0,04233963	-0,5008877	0,019518808
C4B63_14g60	conserved hypothetical protein	-0,402056758	0,008501	-0,4081772	0,013386637
C4B63_20g26	conserved hypothetical protein	-0,40204042	0,04483122	-0,4274802	0,016586616
C4B63_47g60	conserved hypothetical protein	-0,400716189	0,00499329	-0,4592238	0,001345146
C4B63_4g242	protein kinase A catalytic subunit 3	-0,688121206	0,00673586	-0,7733653	0,001176318
C4B63_19g16	Ring finger domain containing protein	-0,675426331	0,03130577	-0,7262018	0,019518808
C4B63_21g63	conserved hypothetical protein	-0,667304955	0,00156322	-0,7542612	0,002876946
C4B63_13g256	conserved hypothetical protein	-0,662083269	0,00888957	-0,7556758	0,002972805
C4B63_112g66	Gamma carbonic anhydrase 2, mitochondrial	-0,6026594	0,00225237	-0,7087577	0,000886895
C4B63_351g33c	calpain-like cysteine peptidase	-0,59539363	0,03837682	-0,703454	0,020072185

Upregulated genes

ID	Annotation	Hemin log2FC	Hemin padj	Hb log2FC	Hb padj
C4B63_9g516	heat shock protein Hsp20	1,00565677	0,00000103	0,8758497	0,0000124
C4B63_61g94	conserved hypothetical protein	0,85504432	0,00240857	0,81983049	0,00055612
C4B63_35g168	3-Beta-hydroxysteroid-delta(8), delta(7)-isomerase	0,81617516	0,00072982	0,57398802	0,04283621
C4B63_62g90	ribosome biogenesis protein	0,79171957	0,00025571	0,67343914	0,00102674
C4B63_77g26	conserved hypothetical protein	0,78304588	0,01218422	0,60049583	0,03079491
C4B63_34g340	mucin TcSMUGL	0,74058842	0,02682232	0,81464619	0,02189048
C4B63_300g99c	mucin TcSMUGL	0,73458697	0,01122026	0,80646254	0,00502762
C4B63_97g35	P-type H+-ATPase	0,72736986	0,00049114	0,76098308	0,00015099
C4B63_303g16	trypanothione reductase	0,72570056	0,00183416	0,59731379	0,01894475
C4B63_6g562	NAD(P)-dependent steroid dehydrogenase protein	0,72159197	0,00034885	0,61910085	0,00338041
C4B63_44g117	conserved hypothetical protein	0,7175753	2,97E-07	0,73774399	1,18E-08
C4B63_8g483	asparagine synthetase a	0,71569028	0,003819	0,60375666	0,02124831
C4B63_34g336	mucin TcSMUGL	0,71310984	0,00323706	0,66131359	0,0154586
C4B63_80g17	ATP binding protein-like protein	0,70780564	0,00467336	0,47778155	0,04239551
C4B63_180g2	conserved hypothetical protein	0,7021827	0,02523343	0,68791672	0,04833888
C4B63_210g28	Shk1 kinase-binding protein 15	0,69180991	0,01995118	0,52386448	0,03866332
C4B63_61g93	conserved hypothetical protein	0,68931569	0,00110568	0,70136851	0,00011968
C4B63_2g209	Present in the outer mitochondrial membrane proteom	0,685954	0,00068582	0,58746996	0,00506595
C4B63_34g1506c	mucin TcSMUGL	0,68194644	0,0109567	0,69220719	0,00702638
C4B63_42g242	2-hydroxy-3-oxopropionate reductase	0,68156526	0,00025571	0,50133803	0,01808554
C4B63_79g25	conserved hypothetical protein	0,6780471	0,0034413	0,65142486	0,00549148
C4B63_81g18	chaperonin HSP60, mitochondrial precursor(fragment	0,67365943	0,00872053	0,66277033	0,00986659
C4B63_34g329	mucin TcSMUGL	0,67192231	0,01185577	0,68360139	0,03207292
C4B63_6g19	NAD(P)-dependent steroid dehydrogenase protein	0,66648721	0,00076277	0,65986918	0,00110491
C4B63_207g2	fibrillarin	0,66541027	0,01209556	0,57307728	0,03316037
C4B63_34g1490c	mucin TcSMUGL	0,66481496	0,02814741	0,74646015	0,01048459
C4B63_111g29	conserved hypothetical protein	0,66444132	0,00134636	0,58522015	0,00464395
C4B63_61g149	eukaryotic translation initiation factor 3 subunit a	0,66197769	0,00089882	0,59241558	0,00384603
C4B63_34g1494c	mucin TcSMUGL	0,66177485	0,01908327	0,63183823	0,03871905
C4B63_11g83	Eukaryotic translation initiation factor 3 subunit F	0,65658775	0,0050611	0,54332214	0,03676837
C4B63_6g244	cation transporter	0,65310657	0,02111985	0,66958391	0,02305951
C4B63_300g96c	mucin TcSMUGL	0,65214362	0,01598036	0,6595291	0,02402572
C4B63_28g84	conserved hypothetical protein	0,65050749	0,00266558	0,68058183	0,00131705
C4B63_25g113	GMP reductase	0,64791169	0,03056201	0,60953006	0,03435989
C4B63_55g67	DNA-directed rna polymerase I largest subunit	0,64739513	0,00031023	0,6400291	2,81E-07
C4B63_85g60	tricarboxylate carrier	0,64717185	0,0009647	0,58648819	0,0023785
C4B63_396g14	Present in the outer mitochondrial membrane proteom	0,64524201	0,00030982	0,67458806	0,00036816
C4B63_20g310	Nucleolar complex protein 4 homolog	0,64391367	0,00980129	0,60174773	0,02683434
C4B63_227g9	mevalonate-diphosphate decarboxylase	0,64304655	0,00216126	0,5321928	0,02083802
C4B63_40g25	Biotin--acetyl-CoA-carboxylase ligase	0,64167702	0,00178396	0,62154638	0,0034175
C4B63_162g33	chaperonin TCP20	0,63891244	0,00025571	0,49928785	0,00935275
C4B63_34g1453c	mucin TcSMUGL	0,63364335	0,0045584	0,6646673	0,0496854
C4B63_300g106c	mucin TcSMUGL	0,63219342	0,0480428	0,72255894	0,04018165
C4B63_6g532	Putative trans-acting enoyl reductase MT2525	0,62986732	0,01344982	0,5209624	0,04977837
C4B63_8g510	conserved hypothetical protein	0,62952073	0,00823687	0,45477568	0,04446288
C4B63_17g90	T-complex protein 1, beta subunit	0,62880306	0,00096261	0,60303708	0,00133323
C4B63_6g227	Eukaryotic translation initiation factor 3 subunit E	0,62590605	0,00690065	0,60722401	0,00747062
C4B63_30g162	cystathionine beta-synthase	0,62568419	0,00820089	0,67161852	0,00279626
C4B63_44g104	diphthine synthase	0,62088082	0,00375278	0,67000289	0,00345095
C4B63_19g82	RNA polymerase I second largest subunit	0,61978041	0,00188651	0,60301829	0,00394033
C4B63_23g116	Importin beta-like SAD2	0,61898145	0,00085953	0,48885278	0,01492251
C4B63_34g1510c	mucin TcSMUGL	0,61576722	0,01553482	0,60991118	0,0302182
C4B63_37g373	ribosome biogenesis protein	0,61265563	0,01031193	0,54708999	0,01671189
C4B63_143g50	cystathionine beta-synthase	0,6104597	0,00342453	0,66297337	0,00018468
C4B63_25g240	Macro domain containing protein	0,5999245	0,02479219	0,47213445	0,00906554
C4B63_13g156	elongation factor 2	0,59852154	0,01250699	0,63839638	0,00781696
C4B63_13g154	elongation factor 2	0,59849943	0,01657612	0,65539508	0,0061175
C4B63_6g236	cation transporter	0,59842951	0,00044033	0,55585436	0,00120102
C4B63_13g155	elongation factor 2	0,59436395	0,02673852	0,62872533	0,01794337
C4B63_90g55	TPR-repeat protein(fragment)	0,58881753	0,00889475	0,5524831	0,01492493
C4B63_13g153	elongation factor 2	0,58657142	0,01906923	0,64886722	0,00764529
C4B63_34g1464c	mucin TcSMUGL	0,58587889	0,04721713	0,66169769	0,01655506
C4B63_143g47	cystathionine beta-synthase	0,58558689	0,01262083	0,56432212	0,00477393
C4B63_134g37	orotidine-5-phosphate decarboxylase/orotate phospho	0,58540324	0,0003152	0,53215102	0,00147212
C4B63_19g205	glycosomal transporter (GAT3)	0,58511246	8,99E-11	0,45356899	0,0000149
C4B63_30g163	cystathionine beta-synthase	0,58485866	0,01050773	0,60372353	0,00097809
C4B63_128g49	nucleolar RNA helicase II	0,58360426	0,01774239	0,53862384	0,03142691
C4B63_38g147	spermidine synthase	0,58240998	0,00170398	0,4547762	0,0234845
C4B63_34g1477c	mucin TcSMUGL	0,57991892	0,02038178	0,66748214	0,00967332
C4B63_112g72	cystathionine beta-synthase	0,57959669	0,00438955	0,53654692	0,00364659
C4B63_13g152	elongation factor 2	0,57683919	0,02230005	0,60632438	0,01725144
C4B63_48g103	Nucleolar complex-associated protein 3	0,5756966	0,03760552	0,61475548	0,01750424
C4B63_57g65	membrane-bound acid phosphatase 2	0,57517147	0,00250675	0,50875166	0,00441221
C4B63_35g130	orotidine-5-phosphate decarboxylase/orotate phospho	0,57296011	0,0000147	0,53407402	0,0002612
C4B63_112g68	cystathionine beta-synthase	0,57160383	0,01242776	0,6156237	0,00341898
C4B63_112g69	cystathionine beta-synthase	0,5687613	0,02676297	0,64621355	0,00249158
C4B63_8g191	JmjC domain-containing protein E	0,56635473	0,0480428	0,60590944	0,04367625
C4B63_70g55	cystathionine beta-synthase	0,5660676	0,01024739	0,64949846	0,0001682
C4B63_143g51	cystathionine beta-synthase	0,56340737	0,00506352	0,6446021	0,00018851
C4B63_214g13	UDP-Gal or UDP-GlcNAc-dependent glycosyltransfera	0,55886857	0,01878425	0,61468403	0,00863432
C4B63_70g51	cystathionine beta-synthase	0,55761122	0,03686757	0,69145061	0,00093842
C4B63_34g1519c	mucin TcSMUGL	0,55555292	0,03896009	0,67908185	0,02643896
C4B63_28g107	malic enzyme	0,55542601	0,00012244	0,4421036	0,01487071
C4B63_4g220	conserved hypothetical protein	0,55072293	0,00271303	0,40129803	0,0423981
C4B63_70g50	cystathionine beta-synthase	0,54867342	0,02363591	0,62189795	0,00155221
C4B63_83g68	protein phosphatase 2C	0,54776652	0,04617105	0,70481942	0,00427412
C4B63_19g195	Nuclear pore complex protein 158	0,54690978	0,00018509	0,46788112	0,00173695
C4B63_1g57	AAA ATPase	0,54671301	0,00404143	0,50514117	0,0097805
C4B63_9g446	Probable methyltransferase TARBP1	0,545777	0,02941617	0,58045248	0,01581239
C4B63_70g53	cystathionine beta-synthase	0,54437063	0,01002424	0,67283396	0,0000763
C4B63_81g19	chaperonin HSP60, mitochondrial precursor	0,54294315	0,03799918	0,57413201	0,02175757
C4B63_157g32	inosine-5'-monophosphate dehydrogenase	0,54151834	0,0033574	0,4964959	0,00745929
C4B63_70g49	cystathionine beta-synthase	0,5400358	0,03435513	0,55437834	0,01795306
C4B63_70g52	cystathionine beta-synthase	0,53866209	0,02313629	0,64086544	0,00037867
C4B63_13g157	elongation factor 2	0,53811482	0,0224764	0,56718163	0,0136712
C4B63_192g26	conserved hypothetical protein	0,53517449	0,01789637	0,48863487	0,02829124
C4B63_22g292	deoxyhypusine synthase	0,53298327	0,03977727	0,50937501	0,03207292
C4B63_112g70	cystathionine beta-synthase	0,53294212	0,01657612	0,59619668	0,00168277
C4B63_73g1	arginine N-methyltransferase	0,53232929	0,01252195	0,42023489	0,04501288
C4B63_70g54	cystathionine beta-synthase	0,52921401	0,04268918	0,64174066	0,00221407
C4B63_20g219	kinetoplast-associated protein 3	0,52533366	0,01598036	0,45222937	0,04140318
C4B63_48g44	conserved hypothetical protein	0,52474805	0,0361839	0,4827289	0,04053149
C4B63_112g71	cystathionine beta-synthase	0,52356398	0,01214417	0,55057426	0,00180985
C4B63_4g558	phosphatidic acid phosphatase protein	0,52250194	0,00025571	0,40679963	0,03861318
C4B63_28g80	DnaJ chaperone protein	0,52099635	0,00254618	0,41987528	0,03782627
C4B63_1g121c	cytochrome b5-like protein	0,52071391	0,00535749	0,43253747	0,04018165
C4B63_73g16	conserved hypothetical protein	0,52033553	0,00818288	0,45193215	0,02478701
C4B63_22g23	ARM-like helical domain-containing protein	0,51792457	0,00590987	0,42782919	0,03537106
C4B63_15g14	oligosaccharyl transferase subunit	0,51744498	0,0224764	0,65355214	0,0022754
C4B63_38g159	conserved hypothetical protein	0,51680753	0,00292613	0,50827272	0,00657051
C4B63_87g17	pitrilysin-like metalloprotease	0,51418074	0,00039421	0,46093435	0,00068217
C4B63_21g255	Nucleolar protein 56	0,51408525	0,0002601	0,44173325	0,00575627
C4B63_143g48	cystathionine beta-synthase	0,50699345	0,04827331	0,58638098	0,00536874
C4B63_79g37	methionyl-tRNA synthetase	0,50697877	0,02907851	0,45640591	0,04680232
C4B63_41g271	conserved hypothetical protein	0,50679149	0,00238113	0,4584076	0,00256085
C4B63_3g1116	Uncharacterized membrane protein C365.16	0,50583211	0,02116135	0,43176812	0,04415769
C4B63_10g436	SPFH domain / Band 7 family	0,50561827	0,02223769	0,60518696	0,00054743
C4B63_52g54	conserved hypothetical protein	0,50458155	0,01192978	0,50658563	0,01636689
C4B63_6g397	cation transporter	0,50424596	0,00673586	0,46859541	0,01995044
C4B63_44g114	conserved hypothetical protein	0,50376441	0,00205669	0,4395097	0,01491705
C4B63_4g527	mitochondrial carrier protein	0,50341476	0,02919287	0,49071057	0,02203029
C4B63_16g315	mitochondrial DNA primase	0,5033871	0,00377228	0,45885987	0,01434078
C4B63_31g229	orotidine-5-phosphate decarboxylase/orotate phospho	0,5013394	0,00214838	0,52403937	0,00168277
C4B63_81g44	tRNA (cytosine(34)-C(5))-methyltransferase	0,50008642	0,01261366	0,50369468	0,01183407
C4B63_143g21	cystathionine beta-synthase	0,49995494	0,02146504	0,58492292	0,00089212
C4B63_23g260	conserved hypothetical protein	0,49916823	0,01520746	0,51817964	0,00882209
C4B63_57g98	succinyl-CoA ligase	0,49365512	0,03332186	0,52324247	0,01991792
C4B63_61g92	conserved hypothetical protein	0,49358519	0,0229219	0,5786633	0,00000419
C4B63_87g21	conserved hypothetical protein	0,49132026	0,01262083	0,44376032	0,02132118
C4B63_6g113	ribonucleoside-diphosphate reductase large chain	0,49029777	0,00000209	0,44247962	0,0000721
C4B63_200g32	3,2-trans-enoyl-CoA isomerase, mitochondrial precurs	0,48762559	0,00356342	0,42484329	0,03940485
C4B63_28g64	aspartate aminotransferase	0,486138	0,01213982	0,46943569	0,01476379
C4B63_15g16	oligosaccharyl transferase subunit	0,48532529	0,04257318	0,62755077	0,00579132
C4B63_12g235	protein transport protein Sec31	0,4849963	0,02085645	0,49194733	0,02113329
C4B63_183g15	spermidine synthase	0,48395941	0,00290123	0,432845	0,0086069
C4B63_25g232	Tetratricopeptide repeat protein 27 homolog	0,48372352	0,00600191	0,43847064	0,02995353
C4B63_282g15	cation transporter	0,48340099	0,00477139	0,49285965	0,00391881
C4B63_15g12	conserved hypothetical protein	0,48333567	0,02777324	0,49535917	0,01970951
C4B63_97g36	P-type H+-ATPase	0,4759758	0,00159451	0,48078805	0,0020481
C4B63_104g103	zinc transporter	0,47272608	0,02515954	0,81702449	0,0000458
C4B63_97g39	P-type H+-ATPase	0,47023749	0,00127129	0,45453318	0,00085777
C4B63_8g62	Nucleolar protein 10	0,46837475	0,02491629	0,49206505	0,0237951
C4B63_61g95	conserved hypothetical protein	0,46821428	0,03545349	0,54246357	0,0001405
C4B63_4g478	DNA-directed RNA polymerases I and III subunit RPA	0,46638678	0,00351702	0,42665773	0,02880343
C4B63_78g37	cation-transporting ATPase	0,45976909	0,01806238	0,51145767	0,00716037
C4B63_41g139	conserved hypothetical protein	0,45825912	0,03537463	0,46076169	0,03451891
C4B63_138g7	unspecified product	0,45810501	0,02623562	0,52131362	0,0102638
C4B63_25g317	threonine synthase	0,45451885	0,00525016	0,48558048	0,00904096
C4B63_98g4	glucose-6-phosphate 1-dehydrogenase	0,45384891	0,01592939	0,42438913	0,02708413
C4B63_97g34	P-type H+-ATPase	0,44733032	0,00418055	0,46140309	0,00192629
C4B63_25g234	RNA cytidine acetyltransferase	0,4445443	0,01241995	0,46739182	0,01298396
C4B63_63g91	GTPase activating protein	0,44281184	0,01338903	0,42515017	0,02995353
C4B63_42g179	ribosome biogenesis regulatory protein (RRS1)	0,44234566	0,01592939	0,41775503	0,02842148
C4B63_30g129	rrp44p homologue	0,43959459	0,00418055	0,41797321	0,00708454
C4B63_25g117	conserved hypothetical protein	0,43743262	0,02546399	0,45470176	0,03920482
C4B63_30g208	C2 domain/Ankyrin repeats (3 copies)	0,43642244	0,00418596	0,41773989	0,01494838
C4B63_13g173	beta galactofuranosyl glycosyltransferase	0,4359951	0,00601812	0,42762103	0,0068588
C4B63_142g6	GMP synthase	0,4345848	0,00794111	0,42432082	0,02195102
C4B63_39g342	phosphoenolpyruvate mutase	0,42955274	0,0222241	0,45636507	0,01463087
C4B63_31g178	Zinc phosphodiesterase ELAC protein 2	0,42751047	0,03315248	0,48629507	0,02137856
C4B63_34g118	conserved hypothetical protein	0,42513133	0,03633405	0,53965518	0,00463506
C4B63_16g293	inositol 1,4,5-trisphosphate receptor	0,42481488	0,02795553	0,52058878	0,00541714
C4B63_87g34	conserved hypothetical protein	0,42023305	0,03545349	0,44484174	0,04237166
C4B63_194g21	DNA-directed RNA polymerase III	0,41644481	0,01206038	0,43221535	0,01322718
C4B63_18g229	conserved hypothetical protein	0,41008184	0,00268317	0,40045274	0,0101589
C4B63_322g14	amino acid transporter	0,40495851	0,01868402	0,43292788	0,01508389
C4B63_95g32	conserved hypothetical protein	0,40407833	0,01153498	0,44499248	0,00579132
C4B63_11g63	D-alanine--D-alanine ligase	0,40245483	0,023319	0,45124729	0,01387081
C4B63_12g234	protein transport protein Sec31	0,40195016	0,03773879	0,51405996	0,01201982
C4B63_112g84c	Histone H2B variant V	0,4005283	0,03435513	0,41623633	0,02810283

Hemin and Hb common DEGs (t24 vs t0)

Downregulated genes

ID	Annotation	Hemin log2FC	Hemin padj	Hb log2FC	Hb padj
C4B63_49g168	conserved hypothetical protein	-1,4820465	4,9246E-06	-0,9923625	0,004767
C4B63_462g9	glutamamyl carboxypeptidase(fragment)	-1,4450477	3,808E-12	-0,8097054	0,00709218
C4B63_431g10	glutamamyl carboxypeptidase	-0,9316064	3,8466E-05	-0,8216085	0,00242018
C4B63_24g313	protein kinase	-0,9065267	0,00310202	-0,7862406	0,03118586
C4B63_113g47	Generative cell specific 1 protein	-0,8760475	0,000173	-0,7254681	0,00603908
C4B63_244g7	trans-sialidase, Group I	-0,8742289	0,00256955	-0,8005797	0,02415316
C4B63_38g182	UDP-Gal or UDP-GlcNAc-dependent glycosy	-0,8708623	0,00012786	-0,7484477	0,00335613
C4B63_46g90	Ring finger domain containing protein	-0,8505314	0,00713798	-0,7717359	0,03505683
C4B63_37g189	trans-sialidase, Group VI	-0,8424785	0,00013523	-0,942598	0,00038415
C4B63_1g941	trans-sialidase, Group V	-0,832428	0,00024837	-0,7110851	0,02539752
C4B63_336g1	glutamamyl carboxypeptidase(fragment)	-0,9848533	1,6036E-05	-0,692245	0,00814607
C4B63_277g11	glutamamyl carboxypeptidase	-0,9194587	0,00025793	-0,5715871	0,03097346
C4B63_18g330	zinc-finger protein ZFP1	-0,8838695	0,00070028	-0,6614627	0,04688151
C4B63_67g26	trans-sialidase, Group II	-0,8623343	6,2322E-06	-0,5158722	0,03586942
C4B63_38g186	UDP-Gal or UDP-GlcNAc-dependent glycosy	-0,8553927	0,00029553	-0,637564	0,02465589
C4B63_68g79	surface protease GP63	-0,841546	1,609E-05	-0,5748173	0,01934367
C4B63_30g222	conserved hypothetical protein	-0,82799	5,1856E-10	-0,5997656	8,9473E-05
C4B63_170g1	conserved hypothetical protein	-0,8080193	0,00071953	-0,597052	0,03776192
C4B63_2g309	NIMA-related kinase	-0,7745527	0,00148491	-0,6132738	0,04086727
C4B63_194g17	phosphate-repressible phosphate permease	-0,7626285	0,0014678	-0,5696705	0,01770816
C4B63_21g18	arginine permease	-0,7457621	0,00020112	-0,6864553	0,00311501
C4B63_73g11	trans-sialidase, Group IV	-0,7361813	2,7288E-06	-0,5749244	0,00091143
C4B63_22g322	phosphate-repressible phosphate permease	-0,722235	0,0026325	-0,5542708	0,02234822
C4B63_26g286	arginine kinase	-0,7173933	6,9338E-05	-0,5553001	0,00784888
C4B63_351g3	calpain-like cysteine peptidase	-0,6667155	3,4856E-06	-0,5673555	0,00267818
C4B63_26g282	arginine kinase	-0,6474766	1,481E-05	-0,4742744	0,03471292
C4B63_5g393	trans-sialidase, Group V	-0,6413421	0,00099043	-0,4921271	0,02257767
C4B63_23g195	3', 5'-cyclic nucleotide phosphodiesterase	-0,6274947	0,00485219	-0,6173805	0,01298472
C4B63_5g381	mucin TcMUCII	-0,6268937	0,01460375	-0,5796701	0,01147814
C4B63_194g16	phosphate-repressible phosphate permease	-0,6199089	0,00275003	-0,4997368	0,02415316
C4B63_11g60	CHAP domain containing protein	-0,6183338	1,71E-05	-0,4118007	0,04309845
C4B63_21g11	arginine permease	-0,6162036	0,00076292	-0,5559931	0,00699831
C4B63_22g321	phosphate-repressible phosphate permease	-0,6001904	0,00278047	-0,4752943	0,01776837
C4B63_194g15	phosphate-repressible phosphate permease	-0,5975142	0,00482966	-0,4719681	0,02792077
C4B63_21g6	arginine permease	-0,597169	0,00356621	-0,5571367	0,01476544
C4B63_21g5	arginine permease	-0,5917919	0,00230408	-0,5978739	0,00450003
C4B63_3g1092	alanine racemase	-0,5909575	0,00035992	-0,4353996	0,01608889
C4B63_13g146	conserved hypothetical protein	-0,5899994	0,00356034	-0,5558323	0,0217109
C4B63_14g28	hexose transporter	-0,5820395	0,00295405	-0,4670699	0,04606434
C4B63_14g30	hexose transporter	-0,5588023	0,00393427	-0,4545523	0,04795767
C4B63_23g226	conserved hypothetical protein	-0,5481731	0,01108199	-0,4739495	0,03657006
C4B63_1g480	trans-sialidase, Group V	-0,5412182	0,0094045	-0,4664758	0,04118501
C4B63_9g387	conserved hypothetical protein	-0,541077	0,00387348	-0,4137201	0,04077069
C4B63_14g5	hexose transporter	-0,5385089	0,00208192	-0,4467362	0,03423827
C4B63_10g157	Double RNA binding domain protein 6A	-0,5343551	0,00025745	-0,4387388	0,02172544
C4B63_44g230	amino acid transporter	-0,5337096	1,494E-05	-0,4214039	0,00807509
C4B63_21g23	arginine permease	-0,5317464	0,0376242	-0,6165957	0,01934367
C4B63_21g27	arginine permease(fragment)	-0,5288013	0,04083064	-0,5486328	0,0449145
C4B63_1g473	trans-sialidase	-0,5252563	0,02813247	-0,5418481	0,0302265
C4B63_2g73	syntaxin12 (t-snare)	-0,5192405	0,03430865	-0,4861094	0,03377429
C4B63_155g17	trans-sialidase, Group II	-0,5089716	0,03567896	-0,5920842	0,01848201
C4B63_3g1089	Alpha-ketoglutarate-dependent dioxygenase	-0,5059747	0,04522976	-0,5792047	0,03355794
C4B63_134g49	trans-sialidase, Group V	-0,4842843	0,04960968	-0,465359	0,0315107
C4B63_27g1015c	conserved hypothetical protein	-0,4352029	0,04065166	-0,5676481	0,00273583
C4B63_70g65	conserved hypothetical protein	-0,4307023	0,0316066	-0,4449862	0,03763091
C4B63_13g317	vesicle-associated membrane protein	-0,4148283	0,02755419	-0,4306619	0,04102151
C4B63_19g130	Dual specificity phosphatase, catalytic doma	-0,4107456	0,03180994	-0,4871743	0,02762162
C4B63_16g183	surface protease GP63	-0,6983272	0,00181378	-0,6148869	0,03082793
C4B63_4g180	serine/threonine protein kinase	-0,6958293	5,3112E-08	-0,4340573	0,00606314
C4B63_109g42	conserved hypothetical protein	-0,6932399	0,00021605	-0,447963	0,02651241
C4B63_21g16	arginine permease	-0,6892532	0,00051401	-0,5702117	0,00915015
C4B63_26g278	arginine kinase	-0,6762975	0,00018654	-0,4819603	0,0124197
C4B63_59g207	mucin TcMUCII	-0,6683352	0,00560751	-0,6326276	0,04795767
C4B63_34g137	trans-sialidase, Group IV	-0,6674387	0,00457838	-0,5151104	0,02247693

Upregulated genes

ID	Annotation	Hemin log2FC	Hemin padj	Hb log2FC	Hb padj
C4B63_86g57	conserved hypothetical protein	0,84682464	0,00112086	0,67742002	0,01928453
C4B63_128g306c	histone H4	0,84021584	0,00104004	0,57959669	0,0315107
C4B63_47g72	enolase	0,83629988	1,9314E-09	0,43897637	0,01564897
C4B63_128g303c	histone H4	0,83380038	2,8641E-05	0,61211345	0,01164414
C4B63_35g168	3-Beta-hydroxysteroid-delta(8), delta(7)-isome	0,83194139	0,00045782	0,6294171	0,03291456
C4B63_188g139c	histone H4	0,8075546	0,00025053	0,53118865	0,01518836
C4B63_188g158c	histone H4	0,79790263	0,00194645	0,6085607	0,01383811
C4B63_42g103	pyridoxal kinase	0,79473904	2,7434E-11	0,54221352	0,00333084
C4B63_15g12	conserved hypothetical protein	0,75667068	7,3023E-05	0,55439804	0,01116528
C4B63_188g156c	histone H4	0,74017191	0,00020369	0,58455985	0,00715299
C4B63_34g287	glycerate kinase	0,73841352	0,00104996	0,61826589	0,01098709
C4B63_6g309	heat shock protein 70 (hsp70)	0,73135285	0,00108363	0,6126736	0,03256411
C4B63_128g304c	histone H4	0,72492085	0,00633283	0,57295188	0,02098887
C4B63_81g18	chaperonin HSP60, mitochondrial precursor(f	0,72158957	0,00354295	0,70216073	0,00814607
C4B63_48g92	chaperonin HSP60, mitochondrial precursor	0,72124751	0,00789907	0,69847339	0,01656392
C4B63_78g23	nucleolar RNA-binding protein	0,71814994	0,00497453	0,62941545	0,0201758
C4B63_113g27	heat shock protein 85(fragment)	0,71402098	2,1268E-05	0,55311182	0,0119577
C4B63_24g243	conserved hypothetical protein	0,69934733	0,00154859	0,59761924	0,01838273
C4B63_116g15	membrane transporter protein	0,69852597	1,8805E-05	0,41231402	0,02575516
C4B63_81g100	chaperonin HSP60, mitochondrial precursor	0,69135537	0,01642763	0,72875163	0,01442013
C4B63_303g8	unspecified product	0,68696833	0,02441798	0,68142274	0,01319114
C4B63_34g289	glycerate kinase	0,68504229	0,00547084	0,59465911	0,02903767
C4B63_4g383	mevalonate-diphosphate decarboxylase	0,68396111	0,00054761	0,53200734	0,02568702
C4B63_133g60	heat shock protein 70 (hsp70)	0,67958987	0,00043907	0,58015773	0,00949918
C4B63_80g17	ATP binding protein-like protein	0,67908623	0,00642933	0,52706602	0,03047809
C4B63_48g86	chaperonin HSP60, mitochondrial precursor	0,67813	0,00731075	0,66772817	0,01250366
C4B63_302g50c	histone H3	0,67486621	0,00767506	0,61154645	0,01814599
C4B63_227g7	transporter	0,67465931	1,298E-06	0,4524177	0,01070649
C4B63_137g40	heat shock protein 70 (hsp70)(fragment)	0,66519229	0,00145635	0,6455262	0,00412809
C4B63_111g29	conserved hypothetical protein	0,66319046	0,00116542	0,54210318	0,01449531
C4B63_102g44	hypoxanthine-guanine phosphoribosyltransfera	0,66193172	0,00103229	0,65406208	0,00860162
C4B63_48g88	chaperonin HSP60, mitochondrial precursor	0,65091831	0,0103198	0,63499458	0,02037429
C4B63_200g32	3,2-trans-enoyl-CoA isomerase, mitochondrial	0,65087413	2,0771E-05	0,47456201	0,02642601
C4B63_81g102	chaperonin HSP60, mitochondrial precursor	0,64048212	0,00625541	0,64221835	0,01025684
C4B63_43g125	centromere/microtubule binding protein cbf5	0,63922269	0,00138183	0,48585402	0,02969068
C4B63_137g38	heat shock protein 70 (hsp70)(fragment)	0,63233175	0,0008588	0,58577639	0,00707817
C4B63_55g66	Fatty-acid amide hydrolase 2	0,62737586	1,8473E-06	0,42269866	0,01389839
C4B63_44g246	nucleolar protein	0,62671318	0,0101438	0,56627159	0,02762162
C4B63_48g90	chaperonin HSP60, mitochondrial precursor	0,62478258	0,01826435	0,6124909	0,02969068
C4B63_184g39	heat shock protein DNAJ(fragment)	0,62451893	0,00049085	0,58217227	0,01105635
C4B63_7g313	amidinotransferase	0,62214283	0,00024549	0,48466031	0,0221592
C4B63_302g22	histone H3	0,61772611	0,01155795	0,63339896	0,00311501
C4B63_137g39	heat shock protein 70 (hsp70)	0,61488824	0,0014939	0,55438106	0,01107133
C4B63_137g32	conserved hypothetical protein	0,61470577	0,00335476	0,54866181	0,01959927
C4B63_35g189	eukaryotic translation initiation factor 1A	0,61364643	0,0003331	0,4430004	0,03134249
C4B63_81g103	chaperonin HSP60, mitochondrial precursor	0,61341298	0,01035614	0,61879589	0,01683944
C4B63_10g436	SPFH domain / Band 7 family	0,61285198	0,00282871	0,42409363	0,04043512
C4B63_70g93	DNA repair and recombination helicase protei	0,61171389	0,00118684	0,6386604	0,00236946
C4B63_207g9	ribosome biogenesis protein	0,60513568	0,00265919	0,50986388	0,03532622
C4B63_48g91	chaperonin HSP60, mitochondrial precursor	0,60505214	0,00993604	0,62691435	0,01382321
C4B63_61g149	eukaryotic translation initiation factor 3 subuni	0,60482749	0,00260084	0,51263869	0,02179193
C4B63_45g239	lysyl-tRNA synthetase	0,60175777	0,03080979	0,60290945	0,03924539
C4B63_79g21	ABC transporter	0,60157753	0,00177924	0,46027655	0,0295281
C4B63_15g15	oligosaccharyl transferase subunit	0,60090255	0,00713798	0,59013954	0,01678208
C4B63_57g88	fibrillarin	0,59906387	0,00022858	0,4694768	0,01515891
C4B63_28g56	TFIIF-stimulated CTD phosphatase	0,59831342	0,00112428	0,60737358	0,00040229
C4B63_48g89	chaperonin HSP60, mitochondrial precursor	0,59760154	0,01248609	0,66808236	0,00603908
C4B63_2g430	heat shock protein	0,59731084	0,01241885	0,55770794	0,02308202
C4B63_81g106	chaperonin HSP60, mitochondrial precursor	0,5928271	0,01192435	0,62726577	0,01333415
C4B63_6g314	heat shock protein 70 (hsp70)	0,59145697	0,00734902	0,60089417	0,00475061
C4B63_47g73	importin beta-1 subunit	0,5911002	0,00031888	0,49081484	0,00764991
C4B63_4g79	ribosome biogenesis protein	0,58684392	0,00319295	0,5831513	0,00357173
C4B63_45g126	RNA recognition motif (a.k.a. RRM, RBD, or R	0,58634314	0,03361262	0,55689867	0,03637643
C4B63_6g316	heat shock protein 70 (hsp70)	0,5842611	0,00282871	0,52708884	0,01403013
C4B63_42g52	mitochondrial oligo U binding protein TBRGG1	0,58382582	0,00656178	0,54900829	0,02096594
C4B63_137g36	heat shock protein 70 (hsp70)(fragment)	0,58382505	0,0018685	0,54379795	0,01574229
C4B63_47g52	deoxyhypusine synthase	0,5814406	0,00767506	0,57918062	0,01043056
C4B63_6g550	NAD(P)-dependent steroid dehydrogenase pr	0,58124862	0,00032466	0,41349721	0,03811443
C4B63_4g454	nucleosome assembly protein	0,57972879	0,00159965	0,46819451	0,03343498
C4B63_23g116	Importin beta-like SAD2	0,57843616	0,00187284	0,47054967	0,02831737
C4B63_6g236	cation transporter	0,5782527	0,00066914	0,48306605	0,01095188
C4B63_263g7	ruvB-like DNA helicase	0,57576938	5,8478E-05	0,44953968	0,01127586
C4B63_80g21	Nucleoside diphosphate kinase	0,57321125	0,00133996	0,47089207	0,03337729
C4B63_6g306	heat shock protein 70 (hsp70)(fragment)	0,56737041	0,00284087	0,55186797	0,0051483
C4B63_255g21	calreticulin	0,56621925	0,02058724	0,98140098	0,00034384
C4B63_40g87	WD40 repeat-containing protein	0,56609794	0,0213207	0,56532353	0,01807531
C4B63_98g4	glucose-6-phosphate 1-dehydrogenase	0,56183659	0,00130596	0,41479165	0,04309845
C4B63_6g530	conserved hypothetical protein	0,55908969	0,0040822	0,42137761	0,03134249
C4B63_114g17	conserved hypothetical protein	0,55832666	0,003484	0,49596946	0,02689726
C4B63_11g9	eukaryotic translation initiation factor 3 subuni	0,5533089	1,6929E-05	0,42048176	0,02533924
C4B63_104g78	calreticulin	0,55175309	0,00734902	0,83196756	0,00307421
C4B63_31g181	mitochondrial DNA polymerase beta	0,54485447	0,04689631	0,63802442	0,01867475
C4B63_6g317	heat shock protein 70 (hsp70)	0,54326611	0,00184098	0,50085813	0,01070649
C4B63_27g204	kinetoplastid kinetochore protein 10	0,54090836	0,00016826	0,52543146	0,00292964
C4B63_7g212	ruvB-like DNA helicase	0,54067799	0,000167	0,43472643	0,01002591
C4B63_18g288	heat shock 70 kDa protein, mitochondrial prec	0,53863403	0,00845981	0,49342213	0,02941505
C4B63_26g252	nucleosome assembly protein-like protein	0,53845026	0,00967836	0,59548368	0,01599704
C4B63_304g13	nucleosome assembly protein	0,53480676	0,0065431	0,48422879	0,03118304
C4B63_20g137	leucine-rich repeat protein (LRRP)	0,53409499	0,00440613	0,51440489	0,01147814
C4B63_2g786	ubiquitin-activating enzyme E1	0,53312506	0,00591041	0,49802033	0,01160387
C4B63_15g16	oligosaccharyl transferase subunit	0,53215426	0,02058034	0,56243101	0,0225059
C4B63_81g17	DNA mismatch repair protein MSH2(fragment)	0,5310749	0,00239454	0,43888977	0,02603642
C4B63_25g313	Putrescine-cadaverine transporter	0,52971223	0,02869056	0,7791029	0,02392037
C4B63_97g36	P-type H+-ATPase	0,52842661	0,00026321	0,47083401	0,00423688
C4B63_73g16	conserved hypothetical protein	0,52838614	0,00610066	0,5074506	0,01317969
C4B63_25g234	RNA cytidine acetyltransferase	0,52402265	0,00167781	0,43301553	0,03274585
C4B63_1g57	AAA ATPase	0,52226581	0,00579738	0,43032324	0,04606434
C4B63_297g9	conserved hypothetical protein	0,52139197	0,00272641	0,46829146	0,00851324
C4B63_207g7	conserved hypothetical protein	0,51678979	0,0003331	0,41562229	0,01382321
C4B63_8g61	heat shock protein 70 (hsp70)	0,51634374	0,01104523	0,64273822	0,00835562
C4B63_9g201	GTP-binding protein	0,51518441	0,02510703	0,47296577	0,02987447
C4B63_97g34	P-type H+-ATPase	0,51298784	0,00054761	0,44537571	0,00490735
C4B63_7g209	Ribonuclease P protein subunit p25-like protei	0,51284848	0,00068153	0,7243265	0,00013651
C4B63_25g192	NAA35	0,50587109	0,00048238	0,52218014	0,00102429
C4B63_396g14	Present in the outer mitochondrial membrane	0,50387812	0,00807801	0,6263755	0,00268131
C4B63_9g396	sedoheptulose-1,7-bisphosphatase	0,49918438	0,01016985	0,48049656	0,03374928
C4B63_19g200	conserved hypothetical protein	0,49825817	0,0006872	0,45855259	0,01608889
C4B63_23g110	UDP-glucose:glycoprotein glucosyltransferase	0,49607403	0,00691897	0,63163925	0,00098442
C4B63_113g39	MRB1-associated protein	0,49344124	0,04595972	0,65378096	0,03586942
C4B63_2g394	kinesin	0,49324471	0,04382442	0,5927005	0,01683944
C4B63_2g209	Present in the outer mitochondrial membrane	0,49252957	0,02586587	0,62064137	0,00465422
C4B63_102g79	conserved hypothetical protein	0,48977455	0,00286164	0,44523118	0,01476544
C4B63_20g19	Nucleoporin	0,48938366	0,00447898	0,51095361	0,00539407
C4B63_7g347	V-type ATPase, A subunit	0,48758259	0,0168315	0,49913714	0,02371205
C4B63_97g19	pyruvate dehydrogenase E1 component alpha	0,48428019	0,01842826	0,5224913	0,01382321
C4B63_17g64	conserved hypothetical protein	0,48134186	0,00164001	0,4409603	0,04795767
C4B63_63g7	eukaryotic translation initiation factor 3 subuni	0,47951804	0,00893282	0,45437061	0,00980138
C4B63_148g30	conserved hypothetical protein	0,47066775	0,04757371	0,52811726	0,04368362
C4B63_43g1	kinesin	0,46654911	0,03002361	0,54668294	0,04032818
C4B63_22g18	conserved hypothetical protein	0,46618146	0,00826681	0,41109959	0,03811443
C4B63_7g410	conserved hypothetical protein	0,46590124	0,00180754	0,42621902	0,00530589
C4B63_23g109	UDP-glucose:glycoprotein glucosyltransferase	0,46289862	0,00087483	0,58826518	0,00131096
C4B63_9g246	FtsJ cell division protein	0,46135062	0,00556788	0,45589766	0,01500857
C4B63_11g74	pyruvate dehydrogenase E1 beta subunit	0,46106904	0,00770536	0,43609408	0,0302265
C4B63_220g12	peroxidoxin	0,46080869	0,00150125	0,46963438	0,00131096
C4B63_16g98	conserved hypothetical protein	0,45908295	0,0001834	0,42678657	0,00210526
C4B63_21g80	conserved hypothetical protein	0,45719755	0,00039076	0,40287295	0,00344409
C4B63_43g161	ATP-dependent RNA helicase SUB2	0,45623742	0,00211271	0,53157775	0,00412809
C4B63_78g28	RNA recognition motif (a.k.a. RRM, RBD, or R	0,45603759	0,00012786	0,4820143	0,00359377
C4B63_142g6	GMP synthase	0,45527172	0,00412689	0,40518391	0,04233645
C4B63_70g112	pumilio-repeat, RNA-binding protein(fragment)	0,45475765	0,01651673	0,48200361	0,02906063
C4B63_26g283	ABC transporter	0,45366683	0,00207594	0,40904223	0,0217109
C4B63_80g38	conserved hypothetical protein	0,45341322	0,02362519	0,40956287	0,04105754
C4B63_10g439	heat shock protein 90	0,44843282	0,00770536	0,64168709	0,00015844
C4B63_183g14	conserved hypothetical protein	0,4463395	0,00551154	0,40126005	0,04246147
C4B63_30g177	condensin complex subunit 2	0,44389126	0,02337148	0,62681857	0,0049972
C4B63_80g37	conserved hypothetical protein	0,44281318	0,03629951	0,49304175	0,03256411
C4B63_76g58	Tripartite attachment complex protein 102	0,44020909	0,01599337	0,40354579	0,02263471
C4B63_63g145	retrotransposon hot spot (RHS) protein	0,4389956	0,03002361	0,47926528	0,01787311
C4B63_149g19	peroxidoxin	0,4369691	0,00494596	0,40661044	0,00814871
C4B63_23g212	serine/threonine-protein kinase	0,43565473	0,00286164	0,48098168	0,00780624
C4B63_76g32	guide RNA associated protein, GAP2	0,43474393	0,00130124	0,4096014	0,00771335
C4B63_61g131	protein kinase	0,43046415	0,00408025	0,48861544	0,02182967
C4B63_2g174	polyadenylate-binding protein 1	0,42687384	0,03877962	0,61903636	0,01328588
C4B63_48g168	AAA ATPase	0,42526537	0,03466712	0,50894465	0,01176158
C4B63_24g172	Uncharacterized protein At3g06530	0,41656066	0,01864706	0,4421163	0,0066879
C4B63_48g134	clathrin heavy chain	0,4158769	0,02692887	0,50782765	0,0205812
C4B63_76g57	Tripartite attachment complex protein 102	0,41485808	0,01554026	0,42927216	0,00599651
C4B63_137g10	Coatomer subunit gamma	0,4012839	0,03991797	0,41620521	0,04417031

Gene Ontology analysis of Hemin and Hb common DEGs (Downregulated, t4 vs t0)

	**BIOLOGICAL PROCESS**									
ID	Name	Bgd count	Result count	Result gene list	Pct of bgd	Fold enrichment	Odds ratio	P-value	Benjamini	Bonferroni
GO:0006522	alanine metabolic process	2	2	C4B63_3g1092,C4	100.0	115.77	inf	7,36E-05	0.00537366517879	0.0107473303576
GO:0009078	pyruvate family amino acid metabolic process	2	2	C4B63_3g1092,C4	100.0	115.77	inf	7,36E-05	0.00537366517879	0.0107473303576
	**MOLECULAR FUNCTION**									
ID	Name	Bgd count	Result count	Result gene list	Pct of bgd	Fold enrichment	Odds ratio	P-value	Benjamini	Bonferroni
GO:0008784	alanine racemase activity	2	2	C4B63_3g1092,C4	100.0	115.77	inf	7,36E-05	0.00191390814587	0.00765563258349
GO:0009055	electron transfer activity	22	3	C4B63_19g141,C4	13.6	15.79	18.85	0.000846756	0.0176125297502	0.088062648751

Gene Ontology analysis of Hemin and Hb common DEGs (Upregulated, t4 vs t0)

	**BIOLOGICAL PROCESS**									
ID	Name	Bgd count	Result count	Result gene list	Pct of bgd	Fold enrichment	Odds ratio	P-value	Benjamini	Bonferroni
GO:0006563	L-serine metabolic process	23	18	C4B63_112g68,C4	78.3	55.87	297.95	3,64E-30	2,20E-28	1,10E-27
GO:0006534	cysteine metabolic process	23	18	C4B63_112g68,C4	78.3	55.87	297.95	3,64E-30	2,20E-28	1,10E-27
GO:0000096	sulfur amino acid metabolic process	26	18	C4B63_112g68,C4	69.2	49.42	186.15	1,64E-28	7,05E-27	4,94E-26
GO:0042254	ribosome biogenesis	76	8	C4B63_207g2,C4E	10.5	7.51	8.8	9,98E-06	7,54E-05	0.00301466282566
GO:0006177	GMP biosynthetic process	6	3	C4B63_142g6,C4E	50.0	35.7	72.17	5,20E-05	0.000356820362851	0.0157000959654
GO:0008202	steroid metabolic process	18	4	C4B63_227g9,C4E	22.2	15.86	20.77	9,63E-05	0.000645962075026	0.0290682933762
GO:0034654	nucleobase-containing compound biosynthetic proce	173	10	C4B63_134g37,C4	5.8	4.13	4.62	0.000148645	0.000919140810392	0.0448909849601
GO:0022618	ribonucleoprotein complex assembly	20	4	C4B63_34g118,C4	20.0	14.28	18.17	0.000149132	0.000919140810392	0.0450378997092
GO:0055085	transmembrane transport	256	12	C4B63_104g103,C	4.7	3.35	3.74	0.000235169	0.0013152092505	0.0710212995268
GO:0019856	pyrimidine nucleobase biosynthetic process	10	3	C4B63_134g37,C4	30.0	21.42	30.92	0.000299366	0.0015587718904	0.090408769643
	**CELLULAR COMPONENT**									
ID	Name	Bgd count	Result count	Result gene list	Pct of bgd	Fold enrichment	Odds ratio	P-value	Benjamini	Bonferroni
GO:0005730	nucleolus	66	6	C4B63_21g255,C4	9.1	6.49	7.36	0.000305012	0.00899786413862	0.0179957282772
	**MOLECULAR FUNCTION**									
ID	Name	Bgd count	Result count	Result gene list	Pct of bgd	Fold enrichment	Odds ratio	P-value	Benjamini	Bonferroni
GO:0016616	oxidoreductase activity, acting on the CH-OH group c	36	6	C4B63_157g32,C4	16.7	11.9	14.77	9,21E-06	0.00066865739191	0.00173978052279
GO:0045182	translation regulator activity	128	9	C4B63_11g83,C4E	7.0	5.02	5.67	7,24E-05	0.00136834043967	0.0136834043967
GO:0003723	RNA binding	425	17	C4B63_11g83,C4E	4.0	2.86	3.25	8,19E-05	0.00140708439298	0.0154779283228
GO:0016879	ligase activity, forming carbon-nitrogen bonds	21	4	C4B63_11g63,C4E	19.0	13.6	17.1	0.000182237	0.00264945063734	0.0344428582854
GO:0003746	translation elongation factor activity	65	6	C4B63_13g152,C4	9.2	6.59	7.48	0.000280455	0.00333281700779	0.0530060486611
GO:0016831	carboxy-lyase activity	24	4	C4B63_134g37,C4	16.7	11.9	14.53	0.000313214	0.00348220440444	0.0591974748755

Gene Ontology analysis of Hemin and Hb common DEGs (Downregulated, t24 vs t0)

	**BIOLOGICAL PROCESS**									
ID	Name	Bgd count	Result count	Result gene list	Pct of bgd	Fold enrichment	Odds ratio	P-value	Benjamini	Bonferroni
GO:0006817	phosphate ion transport	7	5	C4B63_194g15,C4	71.4	113.32	434.23	1,71E-10	2,00E-08	2,00E-08
GO:0006575	cellular modified amino acid metabolic process	25	3	C4B63_26g278,C4	12.0	19.04	22.7	0.000493618	0.00641703521128	0.0577533169015
	**CELLULAR COMPONENT**									
ID	Name	Bgd count	Result count	Result gene list	Pct of bgd	Fold enrichment	Odds ratio	P-value	Benjamini	Bonferroni
GO:0016020	membrane	2776	34	C4B63_109g42,C4	1.2	1.94	3.58	3,71E-06	0.000100137408347	0.000100137408347
	**MOLECULAR FUNCTION**									
ID	Name	Bgd count	Result count	Result gene list	Pct of bgd	Fold enrichment	Odds ratio	P-value	Benjamini	Bonferroni
GO:0016775	phosphotransferase activity, nitrogenous group as ac	3	3	C4B63_26g278,C4	100.0	158.65	inf	2,37E-07	4,08E-06	1,63E-05
GO:0005215	transporter activity	295	8	C4B63_14g28,C4E	2.7	4.3	4.98	0.000470774	0.00406042666327	0.0324834133061

Gene Ontology analysis of Hemin and Hb common DEGs (Upregulated, t24 vs t0)

	**BIOLOGICAL PROCESS**									
ID	Name	Bgd count	Result count	Result gene list	Pct of bgd	Fold enrichment	Odds ratio	P-value	Benjamini	Bonferroni
GO:0006457	protein folding	91	16	C4B63_104g78,C4	17.6	12.55	17.17	8,57E-14	2,70E-11	2,70E-11
GO:0006084	acetyl-CoA metabolic process	6	3	C4B63_11g74,C4E	50.0	35.7	72.17	5,20E-05	0.00456985737375	0.016375927911
GO:0006323	DNA packaging	17	4	C4B63_26g252,C4	23.5	16.8	22.37	7,57E-05	0.00476780231525	0.0238390115762
	**CELLULAR COMPONENT**									
ID	Name	Bgd count	Result count	Result gene list	Pct of bgd	Fold enrichment	Odds ratio	P-value	Benjamini	Bonferroni
GO:0043231	intracellular membrane-bounded organelle	841	28	C4B63_104g78,C4	3.3	2.38	2.86	9,94E-06	0.000216789325111	0.00108394662556
GO:0005694	chromosome	168	11	C4B63_128g303c,	6.5	4.67	5.33	2,18E-05	0.000395237711443	0.00237142626866
GO:0005759	mitochondrial matrix	27	5	C4B63_48g86,C4E	18.5	13.22	16.65	3,13E-05	0.00048718760019	0.00341031320133
GO:0005634	nucleus	424	17	C4B63_128g303c,	4.0	2.86	3.26	7,95E-05	0.000867058068221	0.00867058068221
GO:0032991	protein-containing complex	630	21	C4B63_11g9,C4B(	3.3	2.38	2.73	0.000155310	0.00153898491766	0.0169288340942
GO:1902493	acetyltransferase complex	10	3	C4B63_25g192,C4	30.0	21.42	30.92	0.000299366	0.00191946939834	0.0326309797718
GO:0005852	eukaryotic translation initiation factor 3 complex	10	3	C4B63_11g9,C4B(	30.0	21.42	30.92	0.000299366	0.00191946939834	0.0326309797718
	**MOLECULAR FUNCTION**									
ID	Name	Bgd count	Result count	Result gene list	Pct of bgd	Fold enrichment	Odds ratio	P-value	Benjamini	Bonferroni
GO:1901363	heterocyclic compound binding	2258	74	C4B63_10g439,C4	3.3	2.34	4.61	2,95E-16	2,60E-14	5,19E-14
GO:0000166	nucleotide binding	1411	49	C4B63_10g439,C4	3.5	2.48	3.59	1,46E-10	2,14E-09	2,56E-08
GO:0043167	ion binding	2086	55	C4B63_104g78,C4	2.6	1.88	2.67	1,96E-07	2,15E-06	3,44E-05
GO:0051082	unfolded protein binding	56	7	C4B63_104g78,C4	12.5	8.92	10.62	1,17E-05	0.000120729191527	0.00205239625596
GO:0003676	nucleic acid binding	987	28	C4B63_113g39,C4	2.8	2.03	2.38	0.000178162	0.00142699613415	0.0313566751858
GO:0004738	pyruvate dehydrogenase activity	2	2	C4B63_11g74,C4E	100.0	71.39	inf	0.000194590	0.00142699613415	0.0342479072195
GO:0046527	glucosyltransferase activity	2	2	C4B63_23g109,C4	100.0	71.39	inf	0.000194590	0.00142699613415	0.0342479072195

Table S3. List and Gene Ontology analysis of DEGs common to hemin and hemoglobin conditions in the t_4 vs. t_0 and t_{24} vs. t_0 analysis.

Table S4

Hemin exclusive DEGs (t4 vs. t0)

Downregulated genes				Upregulated genes			
ID	Annotation	log2FC	padj	ID	Annotation	log2FC	padj
C4B63_113g62	kinetoplast DNA-associated protein	-1,491272998	0,006597676	C4B63_1g991	mucin TcMUCII	1,056685652	0,000319915
C4B63_277g19	metallo-peptidase, clan MH, family M18	-1,125696703	0,0001823	C4B63_34g1444c	mucin TcSMUGL	0,848644158	0,006850887
C4B63_550g5	glutamamyl carboxypeptidase(fragment)	-1,080526501	0,000157584	C4B63_340g11	conserved hypothetical protein	0,798725144	0,009805255
C4B63_26g34	mucin TcMUCII	-1,052872168	0,005590545	C4B63_34g1468c	mucin TcSMUGL	0,760880185	0,019128352
C4B63_196g33	unspecified product	-1,024338014	0,011929776	C4B63_84g22	conserved hypothetical protein	0,754999243	0,0000682
C4B63_16g295	RNA-binding protein	-1,011468762	0,033283029	C4B63_32g335	conserved hypothetical protein	0,752660374	0,005420783
C4B63_39g66	trans-sialidase, Group V	-0,998673656	0,002978564	C4B63_300g110c	mucin TcSMUGL	0,750633404	0,011856407
C4B63_251g10	lipase	-0,941622889	0,00569034	C4B63_66g155	mucin TcMUCII	0,750196933	0,010834936
C4B63_294g3	metallo-peptidase, clan MH, family M18	-0,928607714	0,000281783	C4B63_37g169	mucin TcMUCII	0,742790592	0,030493062
C4B63_59g50	mucin TcMUCII	-0,909064577	0,006001915	C4B63_104g104	Shk1 kinase-binding protein 15	0,730721588	0,030558868
C4B63_24g236	microtubule-associated protein Gb4	-0,899693504	0,03686757	C4B63_2g717	conserved hypothetical protein	0,718599066	0,045833354
C4B63_22g60	homocysteine S-methyltransferase	-0,875274723	0,008576744	C4B63_143g34	condensin complex subunit 2	0,703768919	0,005680765
C4B63_323g3	unspecified product	-0,866529977	0,001530751	C4B63_157g25	conserved hypothetical protein	0,694494366	0,012936307
C4B63_93g11	unspecified product	-0,856930551	0,001078579	C4B63_2g293	conserved hypothetical protein	0,688502397	0,018803208
C4B63_37g189	trans-sialidase, Group VI	-0,853893005	0,000123727	C4B63_235g11c	conserved hypothetical protein	0,684189874	0,00411543
C4B63_76g66	conserved hypothetical protein	-0,850441886	0,009913188	C4B63_14g241	trypanothione reductase	0,68365927	0,005063519
C4B63_5g262	retrotransposon hot spot protein (RHS)	-0,846985965	0,013833633	C4B63_71g130	mucin TcMUCII	0,68129353	0,030341622
C4B63_62g85	Proline dehydrogenase, mitochondrial	-0,825991432	0,004973215	C4B63_34g331	unspecified product	0,67494903	0,009858092
C4B63_18g330	zinc-finger protein ZFP1	-0,818825829	0,002318746	C4B63_360g13	unspecified product	0,669991258	0,00586106
C4B63_42g559c	conserved hypothetical protein	-0,817766071	0,00393794	C4B63_230g118c	DNA-dependent RNA polymerases	0,666718759	0,024710912
C4B63_52g133	kinesin-like protein	-0,798216479	0,023321142	C4B63_2g486	Serine-threonine kinase receptor-associated protein	0,648091033	0,002062963
C4B63_156g3	unspecified product	-0,791084441	0,01219801	C4B63_252g23	Serine-threonine kinase receptor-associated protein	0,64690548	0,002875611
C4B63_244g8	trans-sialidase, Group I	-0,774940085	0,049028737	C4B63_109g37	Gar1/Naf1 RNA binding region containing protein	0,641919407	0,002878955
C4B63_9g376	Present in the outer mitochondrial membrane proteome 22	-0,740594834	0,024916294	C4B63_84g49	RNA-binding protein	0,63847623	0,001059232
C4B63_322g4	glutamamyl carboxypeptidase(fragment)	-0,736899039	0,010674259	C4B63_34g1482c	mucin TcSMUGL	0,634373452	0,007326816
C4B63_10g453	ABC transporter	-0,732037826	0,001145357	C4B63_59g110	mucin TcMUCII	0,627143817	0,026762975
C4B63_58g126	ubiquitin-conjugating enzyme variant Kua homologue	-0,729538818	0,002168935	C4B63_300g114c	mucin TcSMUGL	0,609905037	0,011929776
C4B63_59g45	mucin TcMUCII	-0,721508881	0,023635906	C4B63_143g4	conserved hypothetical protein	0,606587012	0,045308219
C4B63_156g4	unspecified product	-0,720004159	0,01392578	C4B63_34g1450c	mucin TcSMUGL	0,605194506	0,015262481
C4B63_24g194	microtubule-associated protein	-0,714920541	0,027428349	C4B63_210g33	p-nitrophenylphosphatase	0,603373903	0,03231057
C4B63_96g38	trans-sialidase, Group V	-0,71124491	0,027024937	C4B63_86g57	conserved hypothetical protein	0,596096694	0,041920586
C4B63_127g46	conserved hypothetical protein	-0,700795782	0,002496736	C4B63_34g1434c	mucin TcSMUGL	0,593241939	0,022370828
C4B63_70g135	protein associated with differentiation 4(fragment)	-0,699226625	0,009967466	C4B63_220g11	Beta propeller protein 1	0,592239267	0,02141336
C4B63_30g275	protein disulfide isomerase	-0,694352114	0,0000251	C4B63_149g18	Beta propeller protein 1	0,591214018	0,043560334
C4B63_6g1868c	DNA-binding protein STK 13740	-0,692719099	0,001683369	C4B63_42g121	L-threonine 3-dehydrogenase	0,590242402	0,012393814
C4B63_47g122	trans-sialidase	-0,692636437	0,010520518	C4B63_184g8	GTP-binding protein	0,588993473	0,006773874
C4B63_63g111	hexose transporter(fragment)	-0,69239724	0,015896957	C4B63_84g67	conserved hypothetical protein	0,585718638	0,030562008
C4B63_65g5	unspecified product	-0,689101	0,017502581	C4B63_34g1486c	mucin TcSMUGL	0,58439393	0,012184223
C4B63_281g9	Ring finger domain containing protein	-0,688110415	0,01590677	C4B63_42g127	L-threonine 3-dehydrogenase	0,580873932	0,009244919
C4B63_133g46	cytosolic malate dehydrogenase	-0,683750214	0,01216936	C4B63_38g39	actin 3	0,576593174	0,001852102
C4B63_24g274	conserved hypothetical protein	-0,683612142	0,002695177	C4B63_34g1437c	mucin TcSMUGL	0,568505126	0,045866402
C4B63_66g68	trans-sialidase, Group V	-0,675641984	0,020840385	C4B63_2g564	mRNA export factor MEX67	0,568430043	0,000000866
C4B63_1g473	trans-sialidase	-0,673702844	0,002926133	C4B63_124g19	surface protease GP63	0,567983869	0,027230336
C4B63_459g7	UDP-Gal or UDP-GlcNAc-dependent glycosyltransferase	-0,669215088	0,010391738	C4B63_42g114	L-threonine 3-dehydrogenase	0,566495214	0,016196089
C4B63_4g132	NLI interacting factor-like phosphatase	-0,66685925	0,006126201	C4B63_34g1498c	mucin TcSMUGL	0,566259148	0,034198536
C4B63_45g145	WD and tetratricopeptide repeats protein 1	-0,665187644	0,026822319	C4B63_18g2	L-threonine 3-dehydrogenase	0,563220635	0,013602762
C4B63_590g2	unspecified product	-0,661990274	0,015921928	C4B63_369g1	conserved hypothetical protein	0,562189688	0,049198405
C4B63_33g147	NADH-ubiquinone oxidoreductase, mitochondrial	-0,659932987	0,010892998	C4B63_42g129	L-threonine 3-dehydrogenase	0,561918177	0,029615322
C4B63_66g170	Mucin-associated surface protein (MASP)	-0,657922994	0,021861596	C4B63_18g130	glutamyl-tRNA synthetase	0,561626078	0,000375376
C4B63_2g317	unspecified product	-0,651132466	0,023635906	C4B63_4g383	mevalonate-diphosphate decarboxylase	0,559633154	0,008313589
C4B63_59g207	mucin TcMUCII	-0,650992214	0,008355943	C4B63_38g38	actin 3	0,559620101	0,008889572
C4B63_25g209	conserved hypothetical protein	-0,647865422	0,023635906	C4B63_42g111	L-threonine 3-dehydrogenase	0,558934223	0,023321142
C4B63_13g328	conserved hypothetical protein	-0,645262507	0,020448846	C4B63_9g201	GTP-binding protein	0,558135972	0,013760194
C4B63_19g266	conserved hypothetical protein	-0,644365748	0,02875939	C4B63_151g36	notchless homolog	0,558066567	0,004031696
C4B63_258g25	lipase	-0,637348544	0,015207458	C4B63_47g35	lysosomal alpha-mannosidase precursor	0,543716829	0,027276024
C4B63_45g123	chaperone protein DNAj	-0,631265699	0,004283349	C4B63_44g192	mitochondrial RNA binding protein	0,540547316	0,00118148
C4B63_10g472	conserved hypothetical protein	-0,626480822	0,019492486	C4B63_9g220	Thioredoxin domain-containing protein PLP3A	0,540198007	0,042836092
C4B63_9g377	Present in the outer mitochondrial membrane proteome 22	-0,626025055	0,007304114	C4B63_129g42	diphthine synthase	0,538907073	0,021465037
C4B63_13g208	conserved hypothetical protein,zinc finger protein family member	-0,62364446	0,006175825	C4B63_28g50	ATP-dependent DEAD/H RNA helicase(fragment)	0,537753105	0,022117697
C4B63_419g2	trans-sialidase	-0,620712433	0,019253706	C4B63_4g144	protein transport protein SEC13	0,536622565	0,005424956
C4B63_45g132	conserved hypothetical protein	-0,619956649	0,014533057	C4B63_20g747c	cyclin-dependent kinases regulatory subunit	0,536556229	0,002377663
C4B63_212g4	protein associated with differentiation 4	-0,619174128	0,043790207	C4B63_171g4	phosphatidic acid phosphatase protein	0,531081048	0,015840078
C4B63_173g8	small GTP-binding protein RAB6	-0,618785099	0,031792661	C4B63_22g17	Leucine Rich Repeat	0,527557744	0,048273311
C4B63_4g545	RNA-binding protein 4	-0,615196022	0,027253468	C4B63_42g118	L-threonine 3-dehydrogenase	0,52674916	0,013171673
C4B63_9g249	conserved hypothetical protein	-0,612428614	0,007350121	C4B63_63g95	poly(A)-specific ribonuclease PARN	0,525293232	0,004163347
C4B63_68g103	trans-sialidase, Group II	-0,610409768	0,001697954	C4B63_161g18	UDP-Gal or UDP-GlcNAc-dependent glycosyltransferase	0,523741555	0,015980364
C4B63_47g125	trans-sialidase	-0,609634333	0,007005061	C4B63_18g8	L-threonine 3-dehydrogenase	0,519095114	0,027215696
C4B63_12g151	conserved hypothetical protein	-0,608122017	0,002190287	C4B63_42g124	L-threonine 3-dehydrogenase	0,518611069	0,015241811
C4B63_89g19	retrotransposon hot spot (RHS) protein	-0,604264899	0,003987376	C4B63_38g146	conserved hypothetical protein	0,517304357	0,002483695
C4B63_1g702	trans-sialidase, Group V	-0,60055567	0,026762975	C4B63_40g159	translation initiation factor 2 subunit	0,516665266	0,020618613
C4B63_15g33c	SELENOT	-0,599380248	0,021735987	C4B63_7g423	proteasome beta 7 subunit	0,51082011	0,040109891
C4B63_301g7	conserved hypothetical protein	-0,597693821	0,005707409	C4B63_56g31	conserved hypothetical protein	0,5087836	0,001932478
C4B63_69g318c	conserved hypothetical protein	-0,595896853	0,009748183	C4B63_2g43	short-chain dehydrogenase	0,507804916	0,01774239
C4B63_97g15	Zinc finger CCCH domain-containing protein 35	-0,593215481	0,001504756	C4B63_7g266	conserved hypothetical protein	0,507570945	0,000416235
C4B63_163g33	protein disulfide isomerase	-0,589596931	0,000459489	C4B63_17g46	U3 small nucleolar ribonucleoprotein protein IMP3	0,504119692	0,033152461
C4B63_53g165	conserved hypothetical protein	-0,58608106	0,003725051	C4B63_34g1514c	mucin TcSMUGL	0,504055747	0,028387565
C4B63_27g184	integral membrane transport protein	-0,584932706	0,000832912	C4B63_286g18	eukaryotic translation initiation factor 3 subunit c	0,503554465	0,007245376
C4B63_455g9	trans-sialidase, Group VII	-0,583034363	0,026955096	C4B63_160g6	poly(A)-specific ribonuclease PARN	0,500982479	0,000862697
C4B63_132g20	trans-sialidase, Group V	-0,582654756	0,024710912	C4B63_18g152	conserved hypothetical protein	0,500017375	0,01392578
C4B63_10g454	ABC transporter(fragment)	-0,58243981	0,033295977	C4B63_52g161	tyrosyl-tRNA synthetase	0,499786807	0,006603861
C4B63_208g27	conserved hypothetical protein	-0,575002805	0,02661247	C4B63_44g207	C-5 sterol desaturase	0,499730532	0,002377609
C4B63_9g234	dolichyl-P-Man:GDP-Man7GlcNAc2-PP-dolichyl alpha-1,6-mannosyl	-0,574410051	0,000233382	C4B63_2g294	40 kDa cyclophilin	0,495373267	0,0000133
C4B63_234g7	WD and tetratricopeptide repeats protein 1	-0,570087186	0,015718876	C4B63_18g11	L-threonine 3-dehydrogenase	0,49158537	0,030312503
C4B63_10g115	conserved hypothetical protein	-0,569718493	0,014140775	C4B63_37g366	thimet oligopeptidase	0,491397312	0,001333412
C4B63_24g275	conserved hypothetical protein	-0,564707684	0,012144168	C4B63_22g22	ARM-like helical domain-containing protein	0,490895744	0,002657255
C4B63_44g243	conserved hypothetical protein	-0,560557058	0,010457873	C4B63_227g2	glucan 1,3-alpha-glucosidase	0,488735006	0,002445833
C4B63_10g455	ABC transporter	-0,55692899	0,022228807	C4B63_34g1502c	mucin TcSMUGL	0,488011657	0,02391789
C4B63_92g56	conserved hypothetical protein	-0,550577734	0,030312503	C4B63_28g56	TFIIF-stimulated CTD phosphatase	0,485537395	0,01392578
C4B63_55g34	Paraflagellar rod protein 2	-0,545408859	0,0000165	C4B63_12g362	UDP-Gal or UDP-GlcNAc-dependent glycosyltransferase	0,484058728	0,033680118
C4B63_48g7	NLI interacting factor-like phosphatase	-0,544613437	0,002533081	C4B63_9g524	eukaryotic translation initiation factor 3 subunit k	0,482928577	0,007199187
C4B63_93g32	unspecified product	-0,543081853	0,044290358	C4B63_4g375	glucan 1,3-alpha-glucosidase	0,482693629	0,008198134
C4B63_51g112	conserved hypothetical protein	-0,54244053	0,036545688	C4B63_4g72	fibrillarin	0,480818342	0,030558868
C4B63_16g93	Uncharacterized protein in mobD 3'region	-0,539062038	0,007546807	C4B63_200g53	CorA-like Mg2+ transporter protein	0,48053743	0,018191323
C4B63_44g125	conserved hypothetical protein	-0,530216991	0,001455235	C4B63_1g70	mitochondrial RNA-binding protein 2	0,479500526	0,016172345
C4B63_20g203	Zinc finger protein, C3H1 type-like	-0,526769702	0,025089488	C4B63_4g118	conserved hypothetical protein	0,4762187	0,017014025
C4B63_61g145	conserved hypothetical protein	-0,52426101	0,035453493	C4B63_62g82	thimet oligopeptidase	0,475666229	0,002650891
C4B63_6g587	N-glycosidase YbiA	-0,523617678	0,048042797	C4B63_114g25	conserved hypothetical protein	0,474925834	0,025463989
C4B63_12g334	cytochrome oxidase subunit VII	-0,522476252	0,002081925	C4B63_39g315	CLP1	0,473055613	0,046197417
C4B63_55g40	Paraflagellar rod protein 2	-0,52016133	0,000843714	C4B63_257g3	amino acid transporter	0,47223583	0,046277832
C4B63_18g326	zinc-finger protein ZFP1	-0,519429976	0,00432494	C4B63_48g110	ATP-dependent DEAD/H RNA helicase	0,471871354	0,001997805
C4B63_55g55	Paraflagellar rod protein 2	-0,519386479	0,000138598	C4B63_127g54	squalene synthase	0,469365124	0,000103291
C4B63_102g335c	conserved hypothetical protein	-0,517492983	0,035557232	C4B63_7g229	Probable 18S rRNA (guanine-N(7))-methyltransferase	0,469109268	0,048103956
C4B63_44g235	Kinesin-13 4	-0,516394857	0,003874503	C4B63_70g98	Optic atrophy 3 protein (OPA3)	0,468903107	0,010466564
C4B63_40g103	conserved hypothetical protein	-0,516293444	0,023635906	C4B63_6g2548c	histone H3	0,46850994	0,046882791
C4B63_2g816	conserved hypothetical protein	-0,516247792	0,002475525	C4B63_7g318	peroxisome biogenesis factor 1	0,467281774	0,017658376
C4B63_9g382	conserved hypothetical protein	-0,515215123	0,001583736	C4B63_5g413	mitochondrial RNA-binding protein 2	0,466389742	0,022476404
C4B63_55g38	Paraflagellar rod protein 2	-0,515158696	0,001234613	C4B63_183g14	conserved hypothetical protein	0,465933536	0,003949475
C4B63_40g30	conserved hypothetical protein	-0,51448305	0,030562008	C4B63_4g219	nucleolar RNA-associated proteins (Nrap)	0,464448514	0,004163347
C4B63_40g102	conserved hypothetical protein	-0,514381695	0,014192706	C4B63_26g212	Transmembrane protein 164 homolog	0,464173265	0,009551305
C4B63_44g183	conserved hypothetical protein	-0,512968853	0,003237062	C4B63_260g15	inosine-5'-monophosphate dehydrogenase	0,463562844	0,02919287
C4B63_16g183	surface protease GP63	-0,512342258	0,037779166	C4B63_7g347	V-type ATPase, A subunit	0,460504505	0,027903449
C4B63_221g9	unspecified product	-0,512170223	0,048751822	C4B63_91g34	conserved hypothetical protein	0,460125847	0,001645365
C4B63_35g53	flagellar calcium-binding protein	-0,512027792	0,001147703	C4B63_22g788c	calpain-like cysteine peptidase	0,455104804	0,032381712
C4B63_98g10	unspecified product	-0,51006962	0,038958985	C4B63_49g65	farnesyl pyrophosphate synthase	0,45496421	0,002957664
C4B63_55g46	Paraflagellar rod protein 2	-0,508065577	0,00072982	C4B63_23g83	Aminopeptidase M1	0,4545205	0,010247388
C4B63_109g41	Trypomastigote, Alanine, Serine and Valine rich protein (TASV), sub	-0,507041981	0,011755777	C4B63_28g180	WASH complex subunit 4	0,454491894	0,020480743
C4B63_154g8	retrotransposon hot spot protein	-0,505952344	0,030856374	C4B63_63g145	retrotransposon hot spot (RHS) protein	0,450542072	0,025731754
C4B63_16g317	oxidoreductase	-0,505669698	0,0408403	C4B63_262g21	retrotransposon hot spot (RHS) protein	0,450188847	0,047217126
C4B63_58g132	conserved hypothetical protein	-0,503698405	0,011445117	C4B63_116g15	membrane transporter protein	0,449914443	0,015241811
C4B63_2g530	conserved hypothetical protein	-0,502452006	0,014124773	C4B63_2g331	polyadenylate-binding protein 1	0,447024029	0,0000767
C4B63_45g115	Protein kinase domain	-0,501420741	0,002062963	C4B63_17g69	phenylalanyl-tRNA synthetase alpha chain	0,445004993	0,017283846
C4B63_37g395	retrotransposon hot spot protein (RHS)	-0,50098391	0,031604693	C4B63_227g7	transporter	0,444896347	0,00440306
C4B63_55g51	Paraflagellar rod protein 2	-0,499817778	0,001016888	C4B63_51g842c	Uncharacterized protein At2g34160	0,444774557	0,000690495
C4B63_24g301	phosphatidylinositol-4-phosphate 5-kinase	-0,499524547	0,002851551	C4B63_73g19	conserved hypothetical protein	0,443708369	0,036939816
C4B63_127g44	conserved hypothetical protein	-0,497132765	0,014724406	C4B63_10g406	High mobility group protein TDP1	0,441978859	0,002978564
C4B63_75g67	Probable ubiquitin carboxyl-terminal hydrolase MINDY-4	-0,4969427	0,014124773	C4B63_45g97	eukaryotic translation initiation factor 3 subunit h	0,439818384	0,015850394
C4B63_24g276	conserved hypothetical protein	-0,49659444	0,028145633	C4B63_52g55	Repetitive proline-rich cell wall protein 2	0,438953418	0,012824198
C4B63_98g42	retrotransposon hot spot (RHS) protein	-0,496409519	0,03775155	C4B63_260g14	inosine-5'-monophosphate dehydrogenase	0,438549213	0,01216936
C4B63_2g828	ankyrin repeat protein	-0,495719634	0,047154589	C4B63_2g744	ALA-interacting subunit 3	0,438083821	0,006845812
C4B63_45g139	serine/threonine protein kinase	-0,495099321	0,024846646	C4B63_38g167	spermidine synthase	0,435744064	0,005892579
C4B63_40g40	conserved hypothetical protein	-0,494353284	0,003819003	C4B63_19g110	Transducin beta-like protein 3	0,435639332	0,007485755
C4B63_12g249	Vacuolar protein 8	-0,494236388	0,027201556	C4B63_253g8	NADPH--cytochrome P450 reductase	0,435024323	0,011262752
C4B63_26g310	ABC transporter	-0,489227653	0,022316819	C4B63_20g1417c	conserved hypothetical protein	0,434039022	0,037111123
C4B63_76g22	cytoskeleton associated protein	-0,487692351	0,012184223	C4B63_63g46	2,3-bisphosphoglycerate-independent phosphoglycerate mutase	0,434029702	0,004668484
C4B63_25g220	5'-3' exoribonuclease B	-0,485715323	0,005892579	C4B63_7g74	centrin	0,43264393	0,009178261
C4B63_14g20	hexose transporter	-0,478689438	0,005273356	C4B63_87g15	Golgi/lysosome glycoprotein 1	0,432459333	0,004859761
C4B63_55g50	Paraflagellar rod protein 2	-0,47771273	0,001916615	C4B63_18g223	eukaryotic translation initiation factor 3 subunit d	0,43199132	0,001798226
C4B63_11g51	Oocyte-specific histone RNA stem-loop-binding protein 2	-0,476909839	0,00225237	C4B63_150g36	membrane-bound acid phosphatase 2	0,431064631	0,040440389
C4B63_97g13	conserved hypothetical protein	-0,475334409	0,000264294	C4B63_11g260	conserved hypothetical protein	0,430863355	0,048042797
C4B63_4g99	conserved hypothetical protein	-0,47505247	0,006735859	C4B63_114g17	conserved hypothetical protein	0,430436159	0,041005498
C4B63_20g162	conserved hypothetical protein	-0,474346224	0,031056965	C4B63_13g174	beta galactofuranosyl glycosyltransferase(fragment)	0,430169443	0,037051781
C4B63_44g214	conserved hypothetical protein	-0,474176522	0,007939695	C4B63_4g223	mannosyltransferase-II	0,430164748	0,014047756
C4B63_21g6	arginine permease	-0,473980627	0,033205876	C4B63_2g289	Hypoxia up-regulated protein 1 (Fragment)	0,429379479	0,014046149
C4B63_19g230	calpain-like cysteine peptidase	-0,473616676	0,012691725	C4B63_93g70	conserved hypothetical protein	0,428108949	0,030804814
C4B63_5g393	trans-sialidase, Group V	-0,473529466	0,027096203	C4B63_6g586	proteasome beta 6 subunit	0,427350565	0,028289309
C4B63_272g23	retrotransposon hot spot (RHS) protein	-0,473475756	0,033205876	C4B63_2g219	conserved hypothetical protein	0,426745342	0,02655658
C4B63_44g240	Coiled-coil domain-containing protein 39	-0,472903071	0,018302268	C4B63_14g39	conserved hypothetical protein	0,426113572	0,025092956
C4B63_9g383	conserved hypothetical protein	-0,471299593	0,019253706	C4B63_23g262	choline/ethanolamine phosphotransferase	0,425146955	0,022224099
C4B63_76g17	conserved hypothetical protein	-0,467463982	0,004608309	C4B63_137g10	Coatomer subunit gamma	0,423310506	0,028394012
C4B63_1g953	trans-sialidase, Group V	-0,465817944	0,042450294	C4B63_32g1134c	BolA-like protein 3	0,421650813	0,005411319
C4B63_35g69	flagellar calcium-binding protein	-0,465514979	0,001199326	C4B63_89g45	Mitochondrial SSU ribosomal protein 29	0,421646181	0,034186507
C4B63_20g24	conserved hypothetical protein	-0,464850899	0,023867085	C4B63_52g41	mitochondrial DNA polymerase I protein C	0,419007164	0,000587101
C4B63_6g284	cytochrome c oxidase assembly protein	-0,463833031	0,020746865	C4B63_81g17	DNA mismatch repair protein MSH2(fragment)	0,418564433	0,028096771
C4B63_55g36	Paraflagellar rod protein 2	-0,460306228	0,001919913	C4B63_21g53	RNA-binding protein	0,418007738	0,005892261
C4B63_9g267	paraflagellar rod protein 5	-0,457254072	0,02655658	C4B63_58g87	elongation factor 1-alpha	0,415349381	0,003804811
C4B63_52g170	cytoskeleton associated protein	-0,455708181	0,036694693	C4B63_4g508	peptidyl-prolyl cis-trans isomerase	0,41430609	0,012348363
C4B63_42g257	cyclophilin	-0,455293239	0,004163347	C4B63_60g133	protein tyrosine phosphatase-like protein	0,413456657	0,004877143
C4B63_70g84	serine/threonine protein kinase	-0,454792186	0,022237695	C4B63_4g381	transporter	0,412251902	0,00491455
C4B63_12g230	palmitoyl acyltransferase 7	-0,454712068	0,022921903	C4B63_8g47	telomerase component(fragment)	0,411992342	0,030558868
C4B63_70g86	conserved hypothetical protein	-0,448162239	0,048882077	C4B63_19g111	WD domain, G-beta repeat/Utp13 specific WD40 associated domain	0,411577578	0,031898699
C4B63_17g119	Phosphatidylinositol/phosphatidylcholine transfer protein SFH14	-0,447353191	0,024502938	C4B63_58g89	elongation factor 1-alpha	0,410267764	0,005141512
C4B63_55g42	Paraflagellar rod protein 2	-0,446516876	0,003339381	C4B63_78g38	lipoate-protein ligase	0,408966569	0,037923906
C4B63_35g62	flagellar calcium-binding protein	-0,445394321	0,010466564	C4B63_2g728	pre-rRNA-processing protein PNO1	0,407126318	0,01392578
C4B63_32g259	Cyclin-U4-1	-0,445012962	0,024455455	C4B63_42g1177c	conserved hypothetical protein	0,40647616	0,029926484
C4B63_267g18	conserved hypothetical protein	-0,444492612	0,041376971	C4B63_13g189	ubiquitin-like protein	0,406106416	0,000413066
C4B63_75g60	NADH-ubiquinone oxidoreductase complex I subunit,FT	-0,443910329	0,01112487	C4B63_2g367	nucleolar GTP-binding protein 1	0,405621897	0,003761897
C4B63_55g53	paraxonemal rod protein PAR2	-0,443186479	0,014121606	C4B63_117g55	inosine-5'-monophosphate dehydrogenase	0,404653432	0,023635906
C4B63_35g51	flagellar calcium-binding protein	-0,439723678	0,019116099	C4B63_9g209	conserved hypothetical protein	0,404319561	0,018155469
C4B63_35g89	flagellar calcium-binding protein	-0,439315465	0,011454237	C4B63_38g299	inosine-5'-monophosphate dehydrogenase	0,403438784	0,014364544
C4B63_35g70	flagellar calcium-binding protein	-0,438596628	0,025703392	C4B63_42g192	N-alpha-acetyltransferase 25, NatB auxiliary subunit	0,402890727	0,007456436
C4B63_35g68	flagellar calcium-binding protein	-0,434666318	0,01769851	C4B63_7g316	ruvB-like DNA helicase	0,402142239	0,009462316
C4B63_69g76	conserved hypothetical protein	-0,433470848	0,048103956	C4B63_52g42	glutathione synthetase	0,401116231	0,004715258
C4B63_30g214	conserved hypothetical protein	-0,432572876	0,000718111	C4B63_57g75	dihydrolipoamide acetyltransferase precursor	0,400774516	0,00569034
C4B63_134g59	trans-sialidase, Group V	-0,432544015	0,033233247	C4B63_45g230	ER membrane protein complex subunit 8	0,400135981	0,010520518
C4B63_150g38	glyceraldehyde 3-phosphate dehydrogenase, cytosolic	-0,431419891	0,043066751				
C4B63_35g82	flagellar calcium-binding protein	-0,429035936	0,021960253				
C4B63_231g31	flagellar calcium-binding protein	-0,428371294	0,012000612				
C4B63_51g183	Cilia- and flagella-associated protein 73	-0,427139204	0,017867172				
C4B63_26g201	cAMP specific phosphodiesterase	-0,426616536	0,006001915				
C4B63_35g97	flagellar calcium-binding protein	-0,424666891	0,004549684				
C4B63_35g71	flagellar calcium-binding protein	-0,423366036	0,005517684				
C4B63_17g196	calpain-like cysteine peptidase	-0,423293131	0,01906923				
C4B63_55g48	Paraflagellar rod protein 2	-0,422094472	0,008236867				
C4B63_35g81	flagellar calcium-binding protein	-0,421951986	0,019285399				
C4B63_35g84	flagellar calcium-binding protein	-0,420876196	0,022141478				
C4B63_35g75	flagellar calcium-binding protein	-0,419683251	0,005357488				
C4B63_167g40	trans-sialidase, Group I	-0,419425503	0,045560957				
C4B63_80g15	cAMP response protein 1	-0,418010319	0,023146503				
C4B63_54g35	protein kinase	-0,417547864	0,010362352				
C4B63_4g98	conserved hypothetical protein	-0,416407471	0,004389548				
C4B63_18g16	GTPase activating protein	-0,415425802	0,007637706				
C4B63_231g29	flagellar calcium-binding protein	-0,41410061	0,044556175				
C4B63_35g85	flagellar calcium-binding protein	-0,412549173	0,017283846				
C4B63_35g72	flagellar calcium-binding protein	-0,410888687	0,038311039				
C4B63_95g22	actin-like protein	-0,410831469	0,025871188				
C4B63_51g192	protein kinase A regulatory subunit	-0,410552367	0,02437719				
C4B63_35g80	flagellar calcium-binding protein	-0,409432312	0,014157161				
C4B63_26g198	cAMP specific phosphodiesterase	-0,4078985	0,025485141				
C4B63_259g15	Paladin	-0,405634516	0,032421161				
C4B63_26g243	nucleobase transporter	-0,405460202	0,00894204				
C4B63_145g23	trans-sialidase, Group III	-0,405368026	0,036224661				
C4B63_24g277	conserved hypothetical protein	-0,404361663	0,011474439				
C4B63_9g378	conserved hypothetical protein	-0,400017952	0,039421601				

Hemin exclusive DEGs (t24 vs. t0)

Downregulated genes				Upregulated genes			
ID	Annotation	log2FC	padj	ID	Annotation	log2FC	padj
C4B63_476nc10	unspecified product	-7,524046267	0,00037481	C4B63_10g404	guanine deaminase	0,980741141	1,32797E-05
C4B63_522nc5	unspecified product	-2,757626096	0,039063192	C4B63_348g24	guanine deaminase	0,949438924	0,011176378
C4B63_79g110	conserved hypothetical protein	-1,558581495	0,026583072	C4B63_11g259	membrane transporter protein	0,890016899	0,000414773
C4B63_277g19	metallo-peptidase, clan MH, family M18	-1,372377488	2,87907E-06	C4B63_188g155c	histone H4	0,801843951	0,004253111
C4B63_550g5	glutamamyl carboxypeptidase(fragment)	-1,260698885	6,084E-06	C4B63_183g12	mitochondrial carrier protein	0,793495723	0,000332054
C4B63_24g236	microtubule-associated protein Gb4	-1,227852864	0,001743648	C4B63_128g313c	histone H4	0,791409959	0,002562355
C4B63_113g62	kinetoplast DNA-associated protein	-1,156067027	0,049378282	C4B63_63g4	PSP1 C-terminal conserved region	0,782456752	0,007325919
C4B63_431g2	glutamamyl carboxypeptidase(fragment)	-1,152316274	0,002562355	C4B63_133g1	NAD-specific glutamate dehydrogenase	0,773958435	0,008792691
C4B63_18g39	CRAL/TRIO, N-terminal domain/CRAL/TRIO domain containing protein	-1,140302488	7,52354E-08	C4B63_50g198	Cold-inducible RNA-binding protein	0,76377039	0,00414049
C4B63_76g64	conserved hypothetical protein	-1,106142128	0,005336865	C4B63_6g297	conserved hypothetical protein	0,762987463	0,000224026
C4B63_26g34	mucin TcMUCII	-1,099813999	0,002944967	C4B63_72g114	conserved hypothetical protein	0,735058565	0,004423973
C4B63_60g128	Right handed beta helix region containing protein	-1,065240017	0,049474565	C4B63_485g4	conserved hypothetical protein	0,731161205	0,007080075
C4B63_196g33	unspecified product	-1,047449848	0,008641097	C4B63_38g144	mitochondrial carrier protein	0,727571658	0,000353295
C4B63_46g95	conserved hypothetical protein	-1,025398505	2,54541E-05	C4B63_188g150c	histone H4	0,718128427	0,005779114
C4B63_44g132	multidrug resistance protein E	-1,015255565	0,000106681	C4B63_188g142c	histone H4	0,71551909	0,001836795
C4B63_277g7	glutamamyl carboxypeptidase	-0,988644358	6,32048E-05	C4B63_8g510	conserved hypothetical protein	0,700486786	0,00206211
C4B63_114g5	conserved hypothetical protein	-0,955079632	0,032651482	C4B63_13g305	conserved hypothetical protein	0,695786822	0,000913131
C4B63_46g80	conserved hypothetical protein	-0,951085299	1,39727E-11	C4B63_12g220	pyruvate phosphate dikinase	0,695443693	0,026898793
C4B63_58g126	ubiquitin-conjugating enzyme variant Kua homologue	-0,945836349	2,54541E-05	C4B63_315g8	amino acid transporter	0,688710121	0,008414612
C4B63_27g1020c	RNA-binding protein	-0,941771858	0,000447645	C4B63_25g228	Putative uridine kinase C227.14	0,686161059	0,019679226
C4B63_341g21	lipase	-0,939401502	0,007277508	C4B63_12g221	pyruvate phosphate dikinase	0,663578964	0,032565759
C4B63_294g3	metallo-peptidase, clan MH, family M18	-0,922130514	0,000274856	C4B63_286g15	PSP1 C-terminal conserved region	0,663198107	0,010414819
C4B63_56g41	protein kinase	-0,921589155	0,004011107	C4B63_143g34	condensin complex subunit 2	0,660624507	0,009933383
C4B63_46g96	conserved hypothetical protein	-0,913237196	0,000785887	C4B63_25g113	GMP reductase	0,659598859	0,025756666
C4B63_550g4	glutamamyl carboxypeptidase(fragment)	-0,904984543	6,36279E-05	C4B63_49g106	histone H2A	0,656189228	0,005368603
C4B63_10g453	ABC transporter	-0,891333282	2,71074E-05	C4B63_227g9	mevalonate-diphosphate decarboxylase	0,651319742	0,001517938
C4B63_5g426	trans-sialidase, Group II	-0,886445942	0,016336897	C4B63_39g336	S-adenosylhomocysteine hydrolase	0,648810588	0,020445396
C4B63_9g377	Present in the outer mitochondrial membrane proteome 22	-0,88266273	3,15289E-05	C4B63_6g397	cation transporter	0,642594427	0,000199291
C4B63_9g376	Present in the outer mitochondrial membrane proteome 22	-0,876850919	0,004570017	C4B63_48g25	conserved hypothetical protein	0,642347015	2,17218E-05
C4B63_104g98	lipase	-0,873614871	0,003115083	C4B63_282g15	cation transporter	0,641061325	4,63169E-05
C4B63_341g17	lipase	-0,86681571	0,019587962	C4B63_214g4	amino acid transporter	0,63960715	0,01970888
C4B63_76g66	conserved hypothetical protein	-0,864033364	0,007490529	C4B63_17g257	chromosomal passenger protein	0,639411386	0,008141978
C4B63_71g133	trans-sialidase, Group VI	-0,861793045	0,025898256	C4B63_210g35	3,2-trans-enoyl-CoA isomerase, mitochondrial precursor	0,623425809	4,63169E-05
C4B63_258g25	lipase	-0,8458152	0,000547544	C4B63_13g190	cdc2-related kinase 3	0,623420169	0,009913801
C4B63_244g6	trans-sialidase, Group I	-0,843414883	0,018745063	C4B63_2g223	conserved hypothetical protein	0,620508131	0,015705403
C4B63_322g4	glutamamyl carboxypeptidase(fragment)	-0,831332072	0,00260717	C4B63_18g233	glyceraldehyde 3-phosphate dehydrogenase	0,619366059	0,026898793
C4B63_170g26	conserved hypothetical protein	-0,825300924	4,14849E-07	C4B63_10g449	Triple RNA binding domain protein 3	0,618327253	0,003267656
C4B63_331g5	UDP-Gal or UDP-GlcNAc-dependent glycosyltransferase	-0,823396301	0,036001703	C4B63_302g60c	histone H3	0,61629756	0,027109777
C4B63_139g11	Mucin-associated surface protein (MASP)	-0,817057809	0,010669581	C4B63_25g287	conserved hypothetical protein	0,616162041	0,031292014
C4B63_65g5	unspecified product	-0,810183196	0,003286696	C4B63_147g266c	conserved hypothetical protein	0,615857775	0,043445984
C4B63_44g243	conserved hypothetical protein	-0,805491511	6,19392E-05	C4B63_197g16	Nucleolar protein 10	0,614933133	0,03894059
C4B63_5g689	Mucin-associated surface protein (MASP)	-0,800731848	0,045768344	C4B63_4g381	transporter	0,610914143	3,30738E-06
C4B63_27g201	conserved hypothetical protein	-0,798649471	0,041938564	C4B63_263g4	amidinotransferase	0,601219422	0,000510127
C4B63_46g66	conserved hypothetical protein	-0,786083326	0,001534936	C4B63_60g172	conserved hypothetical protein	0,598632177	0,031179217
C4B63_10g454	ABC transporter(fragment)	-0,769080501	0,002112707	C4B63_18g232	glyceraldehyde 3-phosphate dehydrogenase	0,59858398	0,031230087
C4B63_96g38	trans-sialidase, Group V	-0,761739029	0,015242092	C4B63_58g135	heat shock protein DNAJ	0,590218303	0,011081989
C4B63_46g177	conserved hypothetical protein	-0,756410473	1,7413E-05	C4B63_102g80	kinesin	0,589966662	0,040075561
C4B63_39g146	Mucin-associated surface protein (MASP)	-0,756208729	0,026176204	C4B63_6g562	NAD(P)-dependent steroid dehydrogenase protein	0,589567117	0,00497123
C4B63_21g22	arginine permease(fragment)	-0,754385443	0,001585546	C4B63_210g33	p-nitrophenylphosphatase	0,587618112	0,037423017
C4B63_10g455	ABC transporter	-0,748775059	0,000735618	C4B63_220g11	Beta propeller protein 1	0,585021556	0,022015125
C4B63_251g10	lipase	-0,748479878	0,03612984	C4B63_6g2514c	histone H3	0,583989763	0,035006462
C4B63_69g318c	conserved hypothetical protein	-0,74394346	0,000560111	C4B63_17g80	Staphylococcal nuclease domain-containing protein 1	0,582910667	3,51903E-08
C4B63_2g318	conserved hypothetical protein	-0,740422658	0,019587962	C4B63_91g43	conserved hypothetical protein	0,580169023	0,021876261
C4B63_22g32	conserved hypothetical protein	-0,735978625	0,000331377	C4B63_302g76c	histone H3	0,578943117	0,018596166
C4B63_354g1	electron transfer flavoprotein	-0,729021385	0,00045011	C4B63_76g52	pyrroline-5-carboxylate reductase	0,573909517	4,10915E-05
C4B63_24g194	microtubule-associated protein	-0,728587294	0,022633885	C4B63_7g316	ruvB-like DNA helicase	0,573603579	3,85402E-05
C4B63_109g67	conserved hypothetical protein	-0,725447073	0,000282911	C4B63_2g740	conserved hypothetical protein	0,572743864	0,023048591
C4B63_22g37	conserved hypothetical protein	-0,723623538	0,000224545	C4B63_6g305	heat shock protein 70 (hsp70)	0,572268981	0,017917356
C4B63_17g329	retrotransposon hot spot protein (RHS)	-0,722266815	0,031923171	C4B63_137g25	conserved hypothetical protein	0,567400826	0,015650146
C4B63_11g199	Mucin-associated surface protein (MASP)	-0,713262826	0,003689901	C4B63_84g67	conserved hypothetical protein	0,566932472	0,036955575
C4B63_323g3	unspecified product	-0,709228562	0,011542212	C4B63_83g36	ppnN	0,556545324	0,010888526
C4B63_36g184	trans-sialidase, Group V	-0,7046605	0,006203924	C4B63_80g43	conserved hypothetical protein	0,555326233	0,00866125
C4B63_1g702	trans-sialidase, Group V	-0,703233611	0,006118657	C4B63_19g45	isovaleryl-coA dehydrogenase	0,553881395	1,18064E-05
C4B63_10g472	conserved hypothetical protein	-0,703028112	0,006210407	C4B63_17g81	Staphylococcal nuclease domain-containing protein 1	0,553307191	7,08758E-06
C4B63_22g40	conserved hypothetical protein	-0,698675162	0,000365312	C4B63_25g279	J Biosynthesis Protein 2	0,552109013	0,001384542
C4B63_34g124	conserved hypothetical protein	-0,698524597	2,61393E-07	C4B63_156g14	Succinate-acetate transporter protein/GPR1/FUN34/yaaH family	0,551574906	0,009932793
C4B63_51g215	unspecified product	-0,695417503	0,033510301	C4B63_12g254	conserved hypothetical protein	0,55157336	0,01473025
C4B63_22g28	conserved hypothetical protein	-0,695394428	0,000529199	C4B63_171g4	phosphatidic acid phosphatase protein	0,548256283	0,010805506
C4B63_11g57	CHAP domain containing protein	-0,693551706	4,13934E-08	C4B63_252g23	Serine-threonine kinase receptor-associated protein	0,548046412	0,014899909
C4B63_95g4	unspecified product	-0,692674219	0,041674481	C4B63_2g219	conserved hypothetical protein	0,545781744	0,00193534
C4B63_24g988c	conserved hypothetical protein	-0,685757136	0,008568773	C4B63_26g272	conserved hypothetical protein	0,544108305	0,000468722
C4B63_17g328	retrotransposon hot spot protein (RHS)	-0,684236805	0,041713463	C4B63_51g842c	Uncharacterized protein At2g34160	0,543486057	1,17172E-05
C4B63_21g246	conserved hypothetical protein	-0,682023336	0,011595774	C4B63_43g2	kinesin	0,540619936	0,039335856
C4B63_17g61	conserved hypothetical protein	-0,681595232	0,004411358	C4B63_184g8	GTP-binding protein	0,536639201	0,015376685
C4B63_156g4	unspecified product	-0,681276864	0,020242584	C4B63_2g726	CRAL/TRIO domain containing protein	0,536236175	0,010184858
C4B63_152g45	unspecified product	-0,680126757	0,030970188	C4B63_7g340	beta-fructofuranosidase-like protein	0,534773214	0,019587962
C4B63_13g223	zinc-finger protein family member	-0,676653382	0,033612624	C4B63_4g440	conserved hypothetical protein	0,533839385	0,039093201
C4B63_96g34	trans-sialidase, Group V	-0,676154381	0,042950925	C4B63_13g226	conserved hypothetical protein	0,530950392	0,002861643
C4B63_168g180c	conserved hypothetical protein	-0,674389845	0,013963358	C4B63_32g205	notchless homolog	0,530409619	0,00275003
C4B63_5g369	unspecified product	-0,67225794	0,027878506	C4B63_2g218	PGAP1-like protein/Alpha/beta hydrolase family	0,529546389	0,004144332
C4B63_157g36	trans-sialidase, Group II	-0,667734994	0,016336897	C4B63_52g161	tyrosyl-tRNA synthetase	0,528101333	0,00303343
C4B63_22g738c	calpain-like cysteine peptidase	-0,666649282	1,66337E-10	C4B63_17g101	mitochondrial carrier protein	0,526943063	0,022838453
C4B63_44g179	RNA-binding protein	-0,665658482	0,036533312	C4B63_2g43	short-chain dehydrogenase	0,52630464	0,011749943
C4B63_1g1255	trans-sialidase, Group V	-0,664202507	0,017625445	C4B63_41g251	arginine N-methyltransferase	0,524972825	0,0178062
C4B63_417g21	unspecified product	-0,659228428	0,035607276	C4B63_79g20	conserved hypothetical protein	0,522924594	0,017625445
C4B63_39g138	surface protease GP63	-0,658175321	0,018449635	C4B63_8g467	NMD3 family	0,519458137	0,00065138
C4B63_351g27c	small myristoylated protein-1	-0,655559327	8,97075E-11	C4B63_81g21	Cell division cycle 20.4, cofactor of APC complex	0,517729381	0,013283186
C4B63_211g28	conserved hypothetical protein	-0,652634654	0,019536057	C4B63_86g2	chaperone protein DNAj	0,516095688	0,00206211
C4B63_67g15	trans-sialidase, Group VI	-0,651575213	0,048698031	C4B63_2g251	chaperone protein DNAj	0,512563204	0,008791658
C4B63_3g766	Mucin-associated surface protein (MASP)	-0,650292246	0,028568534	C4B63_112g19	proteasome beta 3 subunit	0,511073068	0,034200193
C4B63_1g792	unspecified product	-0,649102664	0,0280409	C4B63_10g167	conserved hypothetical protein	0,509960221	0,027216143
C4B63_8g240	trans-sialidase, Group VI	-0,648632256	0,035687063	C4B63_49g166	alanyl-tRNA synthetase	0,509604378	7,37971E-05
C4B63_9g383	conserved hypothetical protein	-0,645497274	0,000408049	C4B63_20g747c	cyclin-dependent kinases regulatory subunit	0,507569221	0,003926179
C4B63_66g170	Mucin-associated surface protein (MASP)	-0,643845448	0,023991967	C4B63_40g25	Biotin--acetyl-CoA-carboxylase ligase	0,504234664	0,020247783
C4B63_29g79	unspecified product	-0,639421072	0,023194212	C4B63_42g187	conserved hypothetical protein	0,503695039	0,016698569
C4B63_70g82	protein kinase	-0,633638637	0,000178439	C4B63_51g209	nucleoside 2-deoxyribosyltransferase	0,502615965	0,013048836
C4B63_15g5	zinc-finger CCCH domain containing protein 11	-0,629522983	0,000813155	C4B63_79g37	methionyl-tRNA synthetase	0,498496659	0,031724256
C4B63_13g208	conserved hypothetical protein,zinc finger protein family member	-0,628165956	0,005031804	C4B63_21g281	DNA repair and recombination helicase protein PIF5	0,498269516	0,021762787
C4B63_34g122	Trypomastigote, Alanine, Serine and Valine rich protein (TASV), subfamily C	-0,627936083	0,003581637	C4B63_52g28	catalytic subunit of the vacuolar transporter chaperone 4(fragmen	0,498133577	0,003326697
C4B63_52g153	conserved hypothetical protein	-0,624241045	0,02426574	C4B63_56g42	conserved hypothetical protein	0,497312013	0,04684336
C4B63_29g448	Mucin-associated surface protein (MASP)	-0,623432148	0,027554188	C4B63_2g486	Serine-threonine kinase receptor-associated protein	0,496876605	0,027357346
C4B63_4g242	protein kinase A catalytic subunit 3	-0,622576763	0,015999194	C4B63_20g212	enoyl-CoA hydratase/isomerase family protein	0,496650355	0,00045011
C4B63_181g36	mismatch repair protein MSH4	-0,621635062	0,001983533	C4B63_9g326	ubiquitin-conjugating enzyme	0,496462046	0,034308654
C4B63_323g5	unspecified product	-0,621612344	0,046068546	C4B63_18g61	conserved hypothetical protein	0,493433683	0,03451358
C4B63_397g11	zinc-finger CCCH domain containing protein 11	-0,620718374	0,000585906	C4B63_18g141	MORN repeat-containing protein 1	0,490577449	0,002326654
C4B63_66g68	trans-sialidase, Group V	-0,61925679	0,036409727	C4B63_113g34	ABC transporter	0,488832743	0,00569126
C4B63_16g181	trans-sialidase, Group VIII	-0,617961914	0,014873661	C4B63_114g25	conserved hypothetical protein	0,4880448	0,019274678
C4B63_5g648	unspecified product	-0,617748552	0,049378282	C4B63_84g49	RNA-binding protein	0,487288063	0,019247764
C4B63_47g122	trans-sialidase	-0,614710119	0,026708131	C4B63_308g4	kinetoplastid kinetochore protein 8	0,487017706	0,014562048
C4B63_4g545	RNA-binding protein 4	-0,613587711	0,026745416	C4B63_70g104	Ribosome biogenesis protein BOP1 homolog	0,485963173	0,002147315
C4B63_181g49	mismatch repair protein MSH4(fragment)	-0,61339406	0,049435869	C4B63_353g8	unspecified product	0,485520065	0,018969712
C4B63_3g1037	trans-sialidase, Group V	-0,610026059	0,039582611	C4B63_41g139	conserved hypothetical protein	0,485163312	0,021655529
C4B63_22g320	phosphate-repressible phosphate permease	-0,608253765	0,002065098	C4B63_81g22	dolichyl-P-Man:GDP-Man5GlcNAc2-PP-dolichyl alpha-1,3-manno	0,485103983	0,000474728
C4B63_16g95	conserved hypothetical protein	-0,608088265	0,015705403	C4B63_40g159	translation initiation factor 2 subunit	0,48423633	0,032122551
C4B63_19g141	electron transfer flavoprotein	-0,606089601	0,001010325	C4B63_19g82	RNA polymerase I second largest subunit	0,484088067	0,022049182
C4B63_13g332	RNA-binding protein	-0,604069609	0,035157974	C4B63_4g144	protein transport protein SEC13	0,484046006	0,013908372
C4B63_50g227	Mucin-associated surface protein (MASP)	-0,60128855	0,036143192	C4B63_2g217	PGAP1-like protein/Alpha/beta hydrolase family	0,484027632	0,002953282
C4B63_27g184	integral membrane transport protein	-0,600300724	0,000479068	C4B63_92g48	squalene synthase	0,483191316	0,018648042
C4B63_6g587	N-glycosidase YbiA	-0,599362701	0,017486004	C4B63_2g510	arginyl-tRNA synthetase	0,481395008	0,002323178
C4B63_419g2	trans-sialidase	-0,597676557	0,024417979	C4B63_7g421	coatomer alpha subunit	0,480911068	0,00614814
C4B63_6g611	unspecified product	-0,594107486	0,010184858	C4B63_63g12	protein disulfide isomerase 2	0,478142876	0,035717302
C4B63_11g343	Mucin-associated surface protein (MASP)	-0,590132282	0,016863748	C4B63_14g204	60S acidic ribosomal protein	0,47795789	0,039118382
C4B63_147g63	Mucin-associated surface protein (MASP)	-0,589205026	0,003860385	C4B63_19g205	glycosomal transporter (GAT3)	0,477626853	1,64772E-07
C4B63_427g3	surface protease GP63	-0,589141794	0,011134476	C4B63_14g148	NADH dehydrogenase	0,476425301	6,49989E-05
C4B63_13g256	conserved hypothetical protein	-0,589025276	0,022838453	C4B63_2g289	Hypoxia up-regulated protein 1 (Fragment)	0,476177303	0,004301655
C4B63_1g691	Mucin-associated surface protein (MASP)	-0,587165626	0,014593677	C4B63_57g98	succinyl-CoA ligase	0,475200675	0,041713463
C4B63_260g23	trans-sialidase, Group II	-0,587068188	0,001983576	C4B63_6g19	NAD(P)-dependent steroid dehydrogenase protein	0,474772525	0,028438538
C4B63_60g113	unspecified product	-0,587042482	0,030234176	C4B63_4g118	conserved hypothetical protein	0,474138453	0,016473042
C4B63_3g65	Mucin-associated surface protein (MASP)	-0,582539708	0,045679939	C4B63_14g46	ABC1 family protein YPL109C, mitochondrial	0,473541536	0,008586297
C4B63_5g26	trans-sialidase, Group V	-0,582069127	0,036035045	C4B63_81g96	conserved hypothetical protein	0,473249526	0,028438538
C4B63_149g38	unspecified product	-0,579651127	0,035540043	C4B63_45g97	eukaryotic translation initiation factor 3 subunit h	0,47253608	0,007194625
C4B63_3g207	Mucin-associated surface protein (MASP)	-0,578300302	0,030930299	C4B63_39g1477c	40S ribosomal protein S27	0,471933607	0,02731757
C4B63_41g261	trans-sialidase, Group IV	-0,576353102	0,00353577	C4B63_4g196	conserved hypothetical protein	0,470687861	0,014445349
C4B63_102g58	mismatch repair protein MSH4(fragment)	-0,57630155	0,000388484	C4B63_10g106	kinesin	0,469010572	0,000811106
C4B63_22g773c	small myristoylated protein-1	-0,576067431	5,82486E-09	C4B63_8g485	glutathione peroxidase-like protein	0,46885621	0,000211234
C4B63_1g946	unspecified product	-0,575385566	0,039148457	C4B63_114g4	40S ribosomal protein S15	0,468341086	0,024124458
C4B63_78g32	conserved hypothetical protein	-0,574255011	0,044569796	C4B63_7g318	peroxisome biogenesis factor 1	0,46813526	0,016239705
C4B63_43g74	retrotransposon hot spot (RHS) protein	-0,573325468	0,003420563	C4B63_168g32	conserved hypothetical protein	0,467657032	0,025026692
C4B63_16g35	unspecified product	-0,572971898	0,027026108	C4B63_114g16	conserved hypothetical protein	0,464697673	0,000913
C4B63_20g203	Zinc finger protein, C3H1 type-like	-0,572304943	0,011647401	C4B63_132g317c	40S ribosomal protein S27	0,464398461	0,026261386
C4B63_52g65	Protein LOL4	-0,572061853	0,0013737	C4B63_69g73	fatty acid desaturase	0,463628772	0,001144485
C4B63_19g227	calpain-like cysteine peptidase	-0,570091785	0,021451877	C4B63_516g5	retrotransposon hot spot (RHS) protein	0,46142102	0,046101072
C4B63_43g166	conserved hypothetical protein	-0,569162084	0,027652386	C4B63_163g18	kinesin	0,460880629	0,048340424
C4B63_14g106	conserved hypothetical protein	-0,56521654	6,86062E-06	C4B63_13g169	L-ribulokinase	0,460080934	0,004179992
C4B63_235g1	rhodanese-like domain containing protein	-0,556261235	0,031345961	C4B63_6g161	Eukaryotic initiation factor 4A-1	0,458325682	0,005201504
C4B63_21g17	arginine permease(fragment)	-0,55618565	0,018647063	C4B63_30g178	conserved hypothetical protein	0,456236869	0,003569673
C4B63_41g248	cyclin-like F-box protein	-0,553846716	0,011081989	C4B63_30g260	conserved hypothetical protein	0,455861559	0,034612802
C4B63_47g125	trans-sialidase	-0,552355907	0,016336897	C4B63_114g29	translocon-associated protein (TRAP) alpha subunit	0,45567202	0,005498099
C4B63_3g355	Mucin-associated surface protein (MASP)	-0,552202843	0,049417438	C4B63_18g211	conserved hypothetical protein	0,454350163	0,017704083
C4B63_25g220	5'-3' exoribonuclease B	-0,55169411	0,001083628	C4B63_4g72	fibrillarin	0,453123097	0,044508708
C4B63_203g11	dispersed gene family protein 1 (DGF-1)	-0,550432876	0,003282065	C4B63_233g13	conserved hypothetical protein	0,452367462	0,018264351
C4B63_4g132	NLI interacting factor-like phosphatase	-0,547694747	0,032299244	C4B63_19g134	predicted WD40 repeat protein	0,451943149	0,00084102
C4B63_45g173	unspecified product	-0,547168646	9,57845E-06	C4B63_151g36	notchless homolog	0,451383597	0,027924005
C4B63_37g106	trans-sialidase, Group VI	-0,544205493	0,03296846	C4B63_116g13	Nucleolar complex protein 2 homolog	0,450922792	0,00468062
C4B63_13g331	RNA-binding protein	-0,543995174	0,045985567	C4B63_18g230	conserved hypothetical protein	0,450705438	0,000208154
C4B63_1g1161	trans-sialidase, Group II	-0,543791675	0,021616298	C4B63_81g41	Suppressor of disruption of TFIIS	0,450700667	0,013963358
C4B63_98g20	trans-sialidase, Group IV	-0,541916325	0,018651996	C4B63_22g173	coatomer beta subunit	0,450162543	0,003677323
C4B63_14g43	centrin-3	-0,540729099	0,00353577	C4B63_235g10	conserved hypothetical protein	0,44967799	0,000439412
C4B63_36g170	unspecified product	-0,539066767	0,019759994	C4B63_268g10	conserved hypothetical protein	0,448635692	0,02286846
C4B63_5g424	Mucin-associated surface protein (MASP)	-0,537234226	0,009233534	C4B63_2g331	polyadenylate-binding protein 1	0,448229114	6,25367E-05
C4B63_36g84	unspecified product	-0,537068527	0,013264128	C4B63_2g349	Present in the outer mitochondrial membrane proteome 5	0,447174769	0,002746537
C4B63_166g26	trans-sialidase, Group VIII	-0,535157469	0,001899571	C4B63_353g7	unspecified product	0,447119306	0,018417804
C4B63_278g3	conserved hypothetical protein	-0,534067318	0,016135174	C4B63_30g193	Phosphoethanolamine/phosphocholine phosphatase	0,446872782	0,000408049
C4B63_15g218	Mucin-associated surface protein (MASP)	-0,53174621	0,045782874	C4B63_61g118	conserved hypothetical protein	0,446302264	0,028690559
C4B63_122g12	Domain of unknown function (DUF4464)	-0,52337901	0,048908903	C4B63_335g2	conserved hypothetical protein	0,445570287	0,004640321
C4B63_79g10	mismatch repair protein MSH4	-0,521465151	0,008526442	C4B63_163g20	conserved hypothetical protein	0,443278734	0,022838453
C4B63_4g283	conserved hypothetical protein	-0,520793162	0,001507046	C4B63_4g219	nucleolar RNA-associated proteins (Nrap)	0,443058321	0,006088918
C4B63_137g3	DNA repair and recombination helicase protein PIF1	-0,519892372	0,001562913	C4B63_116g5	P-type H+-ATPase	0,442992343	0,00206211
C4B63_68g91	trans-sialidase, Group VIII	-0,518403059	0,032047901	C4B63_8g517	conserved hypothetical protein	0,44222381	0,002320656
C4B63_45g139	serine/threonine protein kinase	-0,517504917	0,016427633	C4B63_14g55	hook complex protein, conserved	0,442002977	0,002200805
C4B63_246g16	conserved hypothetical protein	-0,515173843	0,012221673	C4B63_7g332	adenosine kinase	0,441882777	0,019003374
C4B63_35g612c	conserved hypothetical protein	-0,512708415	0,034359061	C4B63_7g223	eukaryotic translation initiation factor 3 subunit g	0,44184057	0,002784143
C4B63_73g74	retrotransposon hot spot (RHS) protein	-0,512336643	0,011405621	C4B63_22g312	phosphoglycerate kinase	0,439564434	0,024607989
C4B63_37g122	trans-sialidase, Group V	-0,512028401	0,029534677	C4B63_8g62	Nucleolar protein 10	0,438393099	0,038555382
C4B63_10g424	conserved hypothetical protein	-0,511987226	0,020412065	C4B63_2g395	kinesin	0,43803712	0,028690559
C4B63_97g13	conserved hypothetical protein	-0,508246712	6,48846E-05	C4B63_57g74	conserved hypothetical protein	0,437847743	0,046068546
C4B63_53g165	conserved hypothetical protein	-0,507604441	0,013899202	C4B63_9g324	conserved hypothetical protein	0,436774831	0,002507716
C4B63_109g60	trans-sialidase, Group IV	-0,501879585	0,005195667	C4B63_56g31	conserved hypothetical protein	0,436508571	0,009760243
C4B63_26g309	ABC transporter(fragment)	-0,50156996	0,018648042	C4B63_52g54	conserved hypothetical protein	0,435788537	0,036143192
C4B63_76g17	conserved hypothetical protein	-0,501261035	0,001640006	C4B63_23g23	conserved hypothetical protein	0,435665898	0,017657249
C4B63_45g193	unspecified product	-0,501143386	0,019841527	C4B63_20g226	Probable importin subunit beta-4	0,435576468	0,019430852
C4B63_52g43	protein phosphatase 2C	-0,500262179	0,005649036	C4B63_63g37	oligopeptidase b(fragment)	0,434320164	0,000294927
C4B63_99g112	alanine racemase	-0,500163	0,005870281	C4B63_286g18	eukaryotic translation initiation factor 3 subunit c	0,432905973	0,026099081
C4B63_127g46	conserved hypothetical protein	-0,497287182	0,047573709	C4B63_1g70	mitochondrial RNA-binding protein 2	0,432471105	0,034393341
C4B63_15g385	dispersed gene family protein 1 (DGF-1)	-0,496991266	0,026898793	C4B63_18g152	conserved hypothetical protein	0,431642348	0,041713463
C4B63_24g274	conserved hypothetical protein	-0,495040544	0,046453815	C4B63_44g234	CRAL/TRIO domain containing protein	0,429686227	0,014562362
C4B63_225g35	unspecified product	-0,49382312	0,010188448	C4B63_6g489	Something about silencing protein 10	0,429053307	0,020445396
C4B63_238g9	conserved hypothetical protein	-0,493579953	0,010407949	C4B63_25g231	conserved hypothetical protein	0,428251336	0,000766972
C4B63_44g235	Kinesin-13 4	-0,493200302	0,005511543	C4B63_57g119	conserved hypothetical protein	0,427540145	0,04909789
C4B63_6g572	5'-3' exoribonuclease A	-0,493034456	0,023147956	C4B63_54g169	conserved hypothetical protein	0,427060184	0,048403964
C4B63_12g151	conserved hypothetical protein	-0,489001117	0,018449635	C4B63_2g509	arginyl-tRNA synthetase	0,426291096	0,005947233
C4B63_13g327	conserved hypothetical protein	-0,488346675	0,033612624	C4B63_297g14	phenylalanyl-tRNA synthetase alpha chain	0,424876236	0,003474947
C4B63_39g347	conserved hypothetical protein	-0,486668078	0,035282579	C4B63_10g1870c	conserved hypothetical protein	0,424548898	0,022753296
C4B63_312g23	mismatch repair protein MSH4	-0,484651152	0,018427564	C4B63_128g58	basal body component	0,423558989	0,032831512
C4B63_44g231	amino acid transporter	-0,484143227	0,032912013	C4B63_6g445	prenyltransferase	0,423160425	0,005174677
C4B63_94g84	dispersed gene family protein 1 (DGF-1)	-0,482648176	0,045409958	C4B63_6g279	Coatomer subunit gamma	0,422768378	0,013677847
C4B63_19g218	calpain-like cysteine peptidase	-0,481538192	0,010805506	C4B63_75g68	calcium motive p-type ATPase	0,422321077	0,031849818
C4B63_8g495	Regulator of chromosome condensation (RCC1) repeat	-0,478091699	0,017486004	C4B63_9g314	DNA replication licensing factor MCM3	0,420020624	0,001461073
C4B63_168g15	mismatch repair protein MSH4	-0,477874367	0,000766972	C4B63_322g14	amino acid transporter	0,419529716	0,012624509
C4B63_301g2	conserved hypothetical protein	-0,476736644	0,041713463	C4B63_14g210	WD repeat-containing protein 55	0,417757661	0,047253514
C4B63_171g31	choline/carnitine O-acyltransferase	-0,47511537	0,01428227	C4B63_2g311	conserved hypothetical protein	0,417063735	0,000309296
C4B63_44g183	conserved hypothetical protein	-0,474972141	0,006510894	C4B63_348g21	ADP-ribosylation factor-like protein 3	0,416318009	0,047573709
C4B63_24g275	conserved hypothetical protein	-0,474848749	0,043259471	C4B63_32g30	translationally controlled tumor protein (TCTP)	0,415409061	0,005870281
C4B63_8g466	protein kinase	-0,474582744	0,004774203	C4B63_55g171	kinesin	0,414983572	0,002215459
C4B63_5g698	Mucin-associated surface protein (MASP)	-0,473044979	0,048403964	C4B63_49g78	conserved hypothetical protein	0,414247145	0,027490566
C4B63_97g15	Zinc finger CCCH domain-containing protein 35	-0,471818972	0,016204479	C4B63_11g63	D-alanine--D-alanine ligase	0,414238672	0,017447632
C4B63_70g87	NADH-cytochrome b5 reductase	-0,471470344	0,011783595	C4B63_22g108	Replication Factor C Subunit 1-related protein	0,413618603	0,049520567
C4B63_44g229	amino acid permease	-0,471315434	5,00548E-05	C4B63_38g147	spermidine synthase	0,413525397	0,041938564
C4B63_380g14	conserved hypothetical protein	-0,469784544	0,009420721	C4B63_6g118	Mitochondrial import receptor subunit ATOM46	0,413326375	0,039810592
C4B63_18g308	sphingosine 1-phosphate lyase	-0,469536505	0,006551143	C4B63_32g239	tubulin folding cofactor D	0,413000563	0,002914934
C4B63_13g388	Mucin-associated surface protein (MASP)	-0,466427701	0,0280409	C4B63_23g199	Flagellum attachment zone protein 19	0,411000772	0,007280475
C4B63_12g175	Protein Associated with Differentiation	-0,466249843	0,007730001	C4B63_12g385	Acyl carrier protein, mitochondrial	0,410297182	0,030957158
C4B63_16g142	trans-sialidase, Group II	-0,466073759	0,022623344	C4B63_2g244	conserved hypothetical protein	0,409630016	0,007290437
C4B63_26g310	ABC transporter	-0,465656509	0,030957158	C4B63_30g187	deoxyuridine triphosphatase	0,40945932	0,003626823
C4B63_20g312	conserved hypothetical protein	-0,465439699	0,001552337	C4B63_2g566	phenylalanyl-tRNA synthetase	0,408407001	0,031706884
C4B63_32g51	Failed axon connections homolog	-0,465226692	8,4158E-06	C4B63_10g498	conserved hypothetical protein	0,408156074	0,030957158
C4B63_25g236	conserved hypothetical protein	-0,464641005	0,044320928	C4B63_355g21	conserved hypothetical protein	0,407912977	0,028983564
C4B63_218g22	conserved hypothetical protein	-0,463357047	0,035540043	C4B63_13g271	fructose-bisphosphate aldolase, glycosomal	0,406926353	0,000438924
C4B63_24g225	serine/threonine protein phosphatase	-0,461426564	0,036685181	C4B63_2g305	Hsp70-Hsp90 organizing protein 3	0,406846252	0,024285996
C4B63_32g209	conserved hypothetical protein	-0,454983797	0,004179992	C4B63_38g45	conserved hypothetical protein	0,405345158	0,031115141
C4B63_10g443	conserved hypothetical protein	-0,453798424	0,014394483	C4B63_35g130	orotidine-5-phosphate decarboxylase/orotate phosphoribosyltrans	0,404481855	0,004710586
C4B63_32g53	conserved hypothetical protein	-0,453413302	0,006937298	C4B63_14g39	conserved hypothetical protein	0,404084216	0,035363206
C4B63_16g93	Uncharacterized protein in mobD 3'region	-0,45243949	0,031682345	C4B63_30g253	conserved hypothetical protein	0,403580363	0,037802648
C4B63_47g154	dispersed gene family protein 1 (DGF-1)	-0,450703506	0,023088161	C4B63_28g180	WASH complex subunit 4	0,403435344	0,046102101
C4B63_9g313	DnaJ chaperone protein	-0,450241293	0,036143192	C4B63_19g29	vesicular-fusion ATPase-like protein	0,403039787	0,02114448
C4B63_246g14	conserved hypothetical protein	-0,449072743	0,028748767	C4B63_58g143	Lupus La protein homolog	0,402769488	0,018449635
C4B63_14g20	hexose transporter	-0,449019519	0,009175913	C4B63_3g1120	conserved hypothetical protein	0,401960892	0,002600842
C4B63_246g15	conserved hypothetical protein	-0,448036551	0,03829681	C4B63_123g22	fatty acid desaturase	0,401723413	0,00851259
C4B63_45g174	unspecified product	-0,447265777	0,018836287	C4B63_20g179	CYC2-like cyclin	0,40146757	0,002449243
C4B63_25g189	3'5'-cyclic nucleotide phosphodiesterase	-0,447224633	0,017065484	C4B63_13g270	fructose-bisphosphate aldolase, glycosomal	0,401454738	0,001279679
C4B63_47g162	retrotransposon hot spot (RHS) protein	-0,446982846	0,042950925	C4B63_80g12	RING-variant domain containing protein	0,401406562	0,008107256
C4B63_4g511	zinc-finger protein	-0,446701308	0,048908903	C4B63_23g255	leucine-rich repeat protein (LRRP)	0,401195859	0,026583072
C4B63_16g33	trans-sialidase, Group VIII	-0,445196026	0,030659328	C4B63_23g112	Helicase required for RNAi-mediated heterochromatin assembly 1	0,400812833	0,017459093
C4B63_134g59	trans-sialidase, Group V	-0,44484198	0,026137749	C4B63_10g510	26S proteasome regulatory non-ATPase subunit	0,400297642	0,00723211
C4B63_308g6	conserved hypothetical protein	-0,444210566	0,013048836				
C4B63_2g776	conserved hypothetical protein	-0,442903259	0,034208075				
C4B63_45g175	unspecified product	-0,441754216	0,008112206				
C4B63_314g7	conserved hypothetical protein	-0,439411481	0,000551553				
C4B63_23g16	Battenin	-0,438864703	0,032578847				
C4B63_4g509	FKBP-type peptidylprolyl cis-trans isomerase	-0,437458055	0,001465609				
C4B63_42g60	amastin	-0,43656745	0,001564837				
C4B63_44g227	amino acid permease	-0,435546652	0,000389849				
C4B63_10g416	conserved hypothetical protein	-0,435264038	0,033785049				
C4B63_57g110	conserved hypothetical protein	-0,434949277	0,016176667				
C4B63_75g113	retrotransposon hot spot protein (RHS)	-0,434940924	0,045782874				
C4B63_45g80	conserved hypothetical protein	-0,434729463	0,04277622				
C4B63_18g326	zinc-finger protein ZFP1	-0,434084157	0,021762787				
C4B63_128g242c	dynein light chain	-0,433384672	0,01414948				
C4B63_49g109	protein kinase	-0,431918309	0,045782874				
C4B63_26g240	nucleobase transporter	-0,430711168	0,019437772				
C4B63_51g83	ferric reductase transmembrane protein	-0,430435018	0,00405047				
C4B63_95g6	trans-sialidase, Group V	-0,430190565	0,019143156				
C4B63_18g322	conserved hypothetical protein	-0,428500285	0,011405621				
C4B63_189g32	conserved hypothetical protein	-0,426991806	0,012592775				
C4B63_69g77	protein kinase	-0,425185789	0,011558596				
C4B63_212g8	conserved hypothetical protein	-0,425034461	0,004638435				
C4B63_180g10	retrotransposon hot spot (RHS) protein	-0,423357285	0,031274381				
C4B63_70g84	serine/threonine protein kinase	-0,421733611	0,036409727				
C4B63_51g80	ferric reductase transmembrane protein	-0,419081955	0,000872311				
C4B63_2g33	conserved hypothetical protein	-0,414044128	0,039714977				
C4B63_255g15	UDP-Gal or UDP-GlcNAc-dependent glycosyltransferase	-0,413508827	0,034407072				
C4B63_44g218	conserved hypothetical protein	-0,41311994	0,021040674				
C4B63_17g85	conserved hypothetical protein	-0,412885743	0,009206136				
C4B63_10g420	conserved hypothetical protein	-0,411555506	0,039917965				
C4B63_47g102	cytochrome c oxidase assembly protein	-0,410476915	0,035540043				
C4B63_17g196	calpain-like cysteine peptidase	-0,410180149	0,022838453				
C4B63_2g156	protein kinase A catalytic subunit 3	-0,409506577	0,013340872				
C4B63_6g273	DNA repair and recombination helicase protein PIF1	-0,409293535	0,01612218				
C4B63_57g121	meiosis recombination protein SPO11	-0,406988729	0,034308654				
C4B63_4g335	conserved hypothetical protein	-0,404274081	0,007899072				
C4B63_208g40	conserved hypothetical protein	-0,404052245	0,039917965				
C4B63_5g787	Mucin-associated surface protein (MASP)	-0,403907225	0,046896312				
C4B63_18g169	Zinc finger CCCH domain-containing protein 47	-0,4020007	0,021120659				
C4B63_51g82	ferric reductase transmembrane protein	-0,401274954	0,002914934				
C4B63_40g93	conserved hypothetical protein	-0,400973549	0,005285726				

Hb exclusive DEGs (t4 vs. t0)

Downregulated genes

ID	Annotation	log2FC	padj
C4B63_43g863c	Protein BOLA1, chloroplastic	-1,240155828	0,046271108
C4B63_244g7	trans-sialidase, Group I	-0,908535133	0,005027624
C4B63_20g344	conserved hypothetical protein	-0,824485449	0,03088001
C4B63_235g1	rhodanese-like domain containing protein	-0,822341523	0,000669743
C4B63_244g6	trans-sialidase, Group I	-0,792707358	0,039427911
C4B63_99g109	2OG-Fe(II) oxygenase superfamily	-0,763726033	0,00440518
C4B63_84g51	Coiled-coil domain-containing protein 40 homolog	-0,721547989	0,005545793
C4B63_19g175	Pleckstrin homology domain	-0,712245684	0,01351841
C4B63_7g246	Patatin-like phospholipase domain-containing protein 3	-0,70464162	0,005758503
C4B63_17g109	BCL-6 corepressor	-0,699200892	0,033998151
C4B63_233g27c	conserved hypothetical protein	-0,690051554	0,039893396
C4B63_43g123	conserved hypothetical protein	-0,683464229	0,009561388
C4B63_112g64	conserved hypothetical protein	-0,669165384	0,013804188
C4B63_84g19	conserved hypothetical protein	-0,668550004	0,031620556
C4B63_69g19	exopolyphosphatase	-0,665276472	0,039411221
C4B63_442g10	Uncharacterized protein C4orf22 homolog	-0,662668403	0,040386832
C4B63_3g1089	Alpha-ketoglutarate-dependent dioxygenase AlkB-like superfamily	-0,659607611	0,007816958
C4B63_25g29	trans-sialidase, Group I	-0,650846053	0,04703967
C4B63_26g9	conserved hypothetical protein	-0,646587215	0,009361249
C4B63_170g37	conserved hypothetical protein	-0,64408542	0,042412919
C4B63_7g258	conserved hypothetical protein	-0,637932517	0,023795098
C4B63_27g3	ribonucleoside-diphosphate reductase small chain	-0,629598731	0,037630308
C4B63_122g15	conserved hypothetical protein	-0,62377988	0,039512345
C4B63_69g151	conserved hypothetical protein	-0,614869715	0,004821723
C4B63_16g279	S-adenosyl-L-methionine-dependent methyltransferase	-0,612039104	0,017413919
C4B63_124g35	retrotransposon hot spot (RHS) protein	-0,60941608	0,008436548
C4B63_79g19	Calmodulin-like protein 11	-0,6094081	0,011642421
C4B63_18g795c	zinc-binding protein (Yippee)	-0,60657563	0,033160367
C4B63_122g12	Domain of unknown function (DUF4464)	-0,600072754	0,018913079
C4B63_221g21	conserved hypothetical protein	-0,598743846	0,046114862
C4B63_21g22	arginine permease(fragment)	-0,596267183	0,022722583
C4B63_1g128c	Heat shock protein 67Bb	-0,595297904	0,005545793
C4B63_2g335	conserved hypothetical protein	-0,592469651	0,040374691
C4B63_101g35	delta-4 fatty acid desaturase	-0,591160463	0,032090825
C4B63_280g10	prostaglandin F2alpha synthase	-0,589545686	0,028605053
C4B63_19g130	Dual specificity phosphatase, catalytic domain containing protein	-0,588483857	0,00295146
C4B63_7g251	Calcyphosin-like protein	-0,585586871	0,030507577
C4B63_117g53	protein kinase	-0,581379179	0,035190538
C4B63_2g4640c	conserved hypothetical protein	-0,578954846	0,045587485
C4B63_7g426	Double RNA binding domain protein 7	-0,572482818	0,022722583
C4B63_17g22	Ras-related protein Rab4	-0,56581602	0,030082599
C4B63_80g18	conserved hypothetical protein	-0,554672134	0,010158898
C4B63_2g39c	dynein-associated protein	-0,553342941	0,03825855
C4B63_4g417	conserved hypothetical protein	-0,542094502	0,020868051
C4B63_1g130c	Thiosulfate:glutathione sulfurtransferase	-0,538268357	0,009998568
C4B63_22g10	Enkuring domain-containig protein	-0,537177543	0,030969294
C4B63_18g80	ferric reductase transmembrane protein	-0,535739528	0,020771209
C4B63_11g6	conserved hypothetical protein	-0,53398733	0,01788802
C4B63_12g9	conserved hypothetical protein	-0,530869838	0,022610748
C4B63_4g418	conserved hypothetical protein	-0,526307197	0,01923482
C4B63_23g70	ubiquinone biosynthesis protein COQ7 homolog	-0,526278895	0,034799246
C4B63_17g176	serine carboxypeptidase S28	-0,520264245	0,034622974
C4B63_7g249	conserved hypothetical protein	-0,516884742	0,010227003
C4B63_49g55	conserved hypothetical protein	-0,515236282	0,031387066
C4B63_6g387	NADH dehydrogenase subunit NB6M	-0,512377919	0,018973873
C4B63_18g202	conserved hypothetical protein	-0,512090274	0,034942732
C4B63_184g6	conserved hypothetical protein	-0,51180815	0,007423155
C4B63_52g313c	calpain-like cysteine peptidase	-0,511344679	0,012486342
C4B63_12g182	PUF nine target 1	-0,51100523	0,047311923
C4B63_4g353	conserved hypothetical protein	-0,510356299	0,03882038
C4B63_18g345	conserved hypothetical protein	-0,505430353	0,004225262
C4B63_113g42	thiol-dependent reductase 1	-0,50353573	0,000000461
C4B63_10g278	conserved hypothetical protein	-0,502666865	0,030507577
C4B63_2g326	protein kinase	-0,502600423	0,006764196
C4B63_27g162	conserved hypothetical protein	-0,502502577	0,035191466
C4B63_70g65	conserved hypothetical protein	-0,502091689	0,009559678
C4B63_230g10	A/G-specific adenine glycosylase	-0,498192635	0,040794699
C4B63_2g689	Nucleoside diphosphate kinase 3	-0,496155507	0,039136862
C4B63_8g2705c	conserved hypothetical protein	-0,494716941	0,02478701
C4B63_52g312c	calpain-like cysteine peptidase	-0,494506823	0,028783305
C4B63_54g167	conserved hypothetical protein	-0,4939067	0,045000081
C4B63_4g243	cAMP phosphodiesterase A	-0,490477286	0,021842808
C4B63_127g27	Cilia- and flagella-associated protein 43	-0,490216582	0,014797999
C4B63_9g461	conserved hypothetical protein	-0,488721406	0,040957824
C4B63_7g372	dynein heavy chain	-0,487862576	0,010865466
C4B63_7g430	1,2-Dihydroxy-3-keto-5-methylthiopentene dioxygenase	-0,486247686	0,013638521
C4B63_405g4	glutamate dehydrogenase	-0,481596514	0,015825719
C4B63_17g97	conserved hypothetical protein	-0,480296557	0,010312581
C4B63_12g24	frataxin-like, mitochondrial precursor	-0,478187229	0,007045433
C4B63_14g59	conserved hypothetical protein	-0,476367666	0,000744325
C4B63_31g116	conserved hypothetical protein	-0,47625606	0,020882981
C4B63_20g157	NADH dehydrogenase subunit NI8M	-0,475238491	0,000604385
C4B63_48g142	serine/threonine protein kinase	-0,471603767	0,000543223
C4B63_6g230	conserved hypothetical protein	-0,470926746	0,00382844
C4B63_70g114	conserved hypothetical protein	-0,470254149	0,024888252
C4B63_70g115	protein kinase	-0,468885336	0,04222028
C4B63_47g115	fatty acyl CoA synthetase	-0,466900226	0,001201024
C4B63_83g40	glycine dehydrogenase [decarboxylating](fragment)	-0,46633828	0,049293304
C4B63_116g14	conserved hypothetical protein	-0,46332118	0,010158898
C4B63_14g63	Haloacid dehalogenase-like hydrolase	-0,46273929	0,036774946
C4B63_24g165	protein kinase	-0,461426646	0,011748225
C4B63_7g260	ADP/ATP mitochondrial carrier protein	-0,460657085	0,022722583
C4B63_102g69	katanin	-0,456761908	0,002360843
C4B63_11g265	conserved hypothetical protein	-0,456322449	0,004169035
C4B63_248g14	glucose-6-phosphate isomerase, glycosomal	-0,455948857	0,04351471
C4B63_136g35	conserved hypothetical protein	-0,454900882	0,024505347
C4B63_9g333	cysteine peptidase, Clan CA, family C51	-0,454252022	0,012998286
C4B63_14g209	conserved hypothetical protein	-0,454137608	0,029943886
C4B63_8g561	conserved hypothetical protein	-0,453691677	0,04349771
C4B63_7g438	glutamate dehydrogenase	-0,452746159	0,033548808
C4B63_13g170	pumilio/PUF RNA binding protein 1	-0,451985706	0,002844022
C4B63_43g190	Putative RING-H2 finger protein ATL71	-0,450982055	0,033548808
C4B63_18g267	conserved hypothetical protein	-0,450880374	0,046000233
C4B63_6g531	conserved hypothetical protein	-0,450821969	0,004274116
C4B63_12g259	phosphoinositide-specific phospholipase C	-0,450486051	0,003510809
C4B63_14g106	conserved hypothetical protein	-0,44698023	0,001212559
C4B63_2g63	conserved hypothetical protein	-0,446414405	0,031333477
C4B63_70g547c	conserved hypothetical protein	-0,445408471	0,038613176
C4B63_233g32	ubiquitin hydrolase	-0,445165125	0,039658983
C4B63_191g32	Cilia- and flagella-associated protein 43	-0,443350726	0,021797618
C4B63_1g20	conserved hypothetical protein	-0,441896937	0,04349771
C4B63_133g32	conserved hypothetical protein	-0,441416084	0,04999574
C4B63_113g57	dual specificity protein phosphatase or MAP kinase phosphatase	-0,441299408	0,041433691
C4B63_4g376	NADH-ubiquinone oxidoreductase complex I subunit,FT	-0,44005485	0,001189079
C4B63_18g1035c	conserved hypothetical protein	-0,439695078	0,049133439
C4B63_43g193	conserved hypothetical protein	-0,437672678	0,041572205
C4B63_227g3	NADH-ubiquinone oxidoreductase complex I subunit,FT	-0,437663774	0,011344441
C4B63_70g87	NADH-cytochrome b5 reductase	-0,436191996	0,03797988
C4B63_55g287c	cytochrome c	-0,43442792	0,027698415
C4B63_49g161	conserved hypothetical protein	-0,433401128	0,026700096
C4B63_18g52	protein kinase	-0,431108105	0,036352553
C4B63_120g44	phosphoinositide-specific phospholipase C	-0,429029573	0,015615853
C4B63_41g249	conserved hypothetical protein	-0,426512571	0,009665159
C4B63_39g312	conserved hypothetical protein	-0,426450687	0,033160367
C4B63_57g131	glutamate dehydrogenase	-0,424993219	0,026703969
C4B63_40g74	conserved hypothetical protein	-0,424587111	0,044306022
C4B63_3g1125	short chain dehydrogenase	-0,422310585	0,010158898
C4B63_69g78	protein kinase	-0,421349043	0,025373432
C4B63_289g4	glutamate dehydrogenase	-0,418895697	0,049934421
C4B63_186g5	conserved hypothetical protein	-0,417698373	0,022722583
C4B63_11g480	conserved hypothetical protein	-0,416125112	0,01788802
C4B63_35g55	flagellar calcium-binding protein	-0,415496632	0,021248307
C4B63_17g60	protein kinase	-0,413756847	0,013041359
C4B63_4g211	conserved hypothetical protein	-0,413510454	0,016366894
C4B63_14g155	Leucine-rich repeat-containing protein 74A	-0,41224649	0,02695635
C4B63_7g151	conserved hypothetical protein	-0,411748023	0,00229128
C4B63_12g252	conserved hypothetical protein	-0,411166235	0,003686365
C4B63_47g72	enolase	-0,410044042	0,021004555
C4B63_2g788	conserved hypothetical protein	-0,40932716	0,00277344
C4B63_183g11	conserved hypothetical protein	-0,407142273	0,000691651
C4B63_4g517	conserved hypothetical protein	-0,407102344	0,049133439
C4B63_13g117	conserved hypothetical protein	-0,405940792	0,002365881
C4B63_18g169	Zinc finger CCCH domain-containing protein 47	-0,405778172	0,021162993
C4B63_7g391	ubiquitin hydrolase	-0,404246862	0,028532938
C4B63_25g235	Protein OSCP1	-0,402202918	0,033160367
C4B63_6g259	nucleotidyl transferase	-0,400157258	0,018358864

Upregulated genes

ID	Annotation	log2FC	padj
C4B63_225g40	conserved hypothetical protein	0,748386504	0,00510403
C4B63_34g1472c	mucin TcSMUGL	0,726181544	0,003597327
C4B63_10g159	hypothetical Zinc-finger containing protein	0,722988221	0,012067303
C4B63_46g17	conserved hypothetical protein	0,696284952	0,007419168
C4B63_40g162	exosome-associated protein 4	0,689623871	0,020435922
C4B63_34g1526c	mucin TcSMUGL	0,677343387	0,015410034
C4B63_85g83	Eukaryotic initiation factor 4A-1	0,667834988	0,041562037
C4B63_15g15	oligosaccharyl transferase subunit	0,648235884	0,004714921
C4B63_504g2	unspecified product	0,634033759	0,035623058
C4B63_202g28	cysteine peptidase(fragment)	0,628768225	0,021378564
C4B63_188g148c	histone H4	0,6274261	0,014545101
C4B63_188g147c	histone H4	0,615462782	0,00395163
C4B63_44g113	conserved hypothetical protein	0,615444195	0,007571357
C4B63_9g299	ATP-dependent Clp protease subunit, heat shock protein 104	0,615430167	0,036290641
C4B63_480g17	cysteine peptidase	0,60499424	0,028739129
C4B63_188g145c	histone H4	0,604799599	0,010409787
C4B63_46g16	conserved hypothetical protein	0,600830335	0,02092142
C4B63_9g416	ATP-dependent Clp protease subunit, heat shock protein 104	0,591979759	0,03222943
C4B63_77g10	unspecified product	0,587680085	0,022371984
C4B63_188g154c	histone H4	0,586803851	0,025351461
C4B63_295g15	nucleosome assembly protein-like protein	0,585782087	0,015586429
C4B63_188g132c	histone H4	0,580859917	0,010983823
C4B63_200g30	p-nitrophenylphosphatase	0,580504076	0,016021319
C4B63_35g179	protein tyrosine phosphatase	0,575632998	0,040740429
C4B63_45g84	conserved hypothetical protein	0,573125616	0,01788802
C4B63_41g159	cysteine peptidase(fragment)	0,570377277	0,023003551
C4B63_2g782	ATP-dependent RNA helicase	0,570366618	0,014763793
C4B63_202g15	cysteine peptidase	0,570267867	0,027130166
C4B63_202g24	cysteine peptidase	0,56781098	0,028964441
C4B63_70g48	cystathionine beta-synthase	0,566823963	0,008269429
C4B63_202g17	cysteine peptidase	0,56598157	0,048207729
C4B63_202g25	cysteine peptidase	0,56258199	0,032449213
C4B63_78g13	Proteasome activator complex subunit 4	0,56256771	0,033792728
C4B63_202g20	cysteine peptidase	0,562108552	0,033548808
C4B63_202g27	cysteine peptidase	0,560811356	0,026438959
C4B63_202g16	cysteine peptidase	0,558178326	0,03207292
C4B63_275g2	polyubiquitin	0,557353592	0,04703967
C4B63_302g64c	histone H3	0,55575431	0,030074277
C4B63_89g71	cysteine peptidase	0,555518991	0,040789709
C4B63_89g67	cysteine peptidase	0,553549463	0,035191466
C4B63_89g68	cruzipain precursor	0,551401798	0,035623058
C4B63_41g158	cysteine peptidase	0,549770358	0,005306485
C4B63_202g29	cysteine peptidase(fragment)	0,548707346	0,033377916
C4B63_89g70	cysteine peptidase	0,547321677	0,037826269
C4B63_41g154	cysteine peptidase	0,546557898	0,004292901
C4B63_202g22	cysteine peptidase	0,54490258	0,03825855
C4B63_188g138c	histone H4	0,544456924	0,038296577
C4B63_202g18	cysteine peptidase	0,542799488	0,038944112
C4B63_302g68c	histone H3	0,541564241	0,044157687
C4B63_202g19	cysteine peptidase	0,540518174	0,042398105
C4B63_4g494	conserved hypothetical protein	0,536737557	0,043676254
C4B63_202g13	cysteine peptidase	0,534197282	0,037246791
C4B63_292g20	polyubiquitin	0,53144424	0,046061219
C4B63_42g187	conserved hypothetical protein	0,531153535	0,015083886
C4B63_178g40	conserved hypothetical protein	0,525218731	0,041562037
C4B63_202g23	cysteine peptidase	0,524719545	0,045925699
C4B63_77g15	dispersed gene family protein 1 (DGF-1)	0,524481705	0,043676254
C4B63_202g30	cysteine peptidase(fragment)	0,519534851	0,019797475
C4B63_114g34	conserved hypothetical protein	0,5154172	0,020072185
C4B63_38g165	conserved hypothetical protein	0,514381303	0,003161092
C4B63_188g159c	histone H4	0,512193169	0,034520192
C4B63_480g15	cysteine peptidase	0,511778035	0,027245911
C4B63_202g12	cysteine peptidase	0,509346736	0,040393224
C4B63_7g209	Ribonuclease P protein subunit p25-like protein	0,507884922	0,009996843
C4B63_89g69	cysteine peptidase	0,507183863	0,042521567
C4B63_148g34	pumilio/PUF RNA binding protein 7	0,504219652	0,045583398
C4B63_2g376	ribosome assembly protein RRB1	0,500684629	0,008634324
C4B63_10g167	conserved hypothetical protein	0,497832722	0,027694612
C4B63_302g52c	histone H3	0,496537106	0,030507577
C4B63_188g135c	histone H4	0,49495545	0,021126449
C4B63_40g87	WD40 repeat-containing protein	0,489002256	0,038213273
C4B63_58g56	conserved hypothetical protein	0,488718751	0,002185923
C4B63_128g70	ATP-dependent RNA helicase	0,487320011	0,031474408
C4B63_47g73	importin beta-1 subunit	0,485964654	0,005491483
C4B63_3g1106	peroxisome targeting signal 1 receptor	0,481702746	0,00636503
C4B63_70g93	DNA repair and recombination helicase protein PIF2	0,477279931	0,026901022
C4B63_128g44	major vault protein	0,476863742	0,02840431
C4B63_439g17	cysteine peptidase(fragment)	0,475572304	0,030419999
C4B63_128g61	50S ribosome-binding GTPase	0,474165376	0,005374318
C4B63_19g101	U-rich RNA-binding protein UBP-2	0,474065958	0,033998151
C4B63_51g115	Proliferation-associated protein 2G4	0,474010283	0,047311923
C4B63_135g30	unspecified product	0,471095105	0,040531493
C4B63_202g32	cysteine peptidase	0,468928734	0,037826269
C4B63_41g78	conserved hypothetical protein	0,467309088	0,006932193
C4B63_228g44	conserved hypothetical protein	0,466923098	0,026304888
C4B63_188g156c	histone H4	0,466736018	0,032487206
C4B63_28g51	ATP-dependent DEAD/H RNA helicase(fragment)	0,460530733	0,029696052
C4B63_16g99	cytosolic leucyl aminopeptidase	0,460230365	0,01812296
C4B63_188g130c	histone H4	0,459414542	0,048842294
C4B63_41g153	cysteine peptidase	0,458053543	0,016737791
C4B63_6g2516c	histone H3	0,455181289	0,046952736
C4B63_45g85	conserved hypothetical protein	0,454733608	0,022526483
C4B63_178g43	conserved hypothetical protein	0,453826386	0,03207292
C4B63_371g8	beta galactofuranosyl glycosyltransferase	0,452192364	0,033998151
C4B63_56g75	Vacuolar protein sorting-associated protein VTA1 homolog	0,449255722	0,03207292
C4B63_81g62	glutamine aminotransferase	0,441906455	0,014340776
C4B63_11g495	retrotransposon hot spot protein (RHS)	0,440984832	0,037680795
C4B63_480g11	cysteine peptidase	0,440896523	0,031620556
C4B63_30g940c	Histone H2B variant V	0,435403128	0,022891698
C4B63_308g2	kinetoplast DNA-associated protein(fragment)	0,433955675	0,048487549
C4B63_16g100	cytosolic leucyl aminopeptidase	0,433168356	0,043231054
C4B63_178g46	conserved hypothetical protein	0,428243428	0,02683434
C4B63_87g20	Brefeldin A-inhibited guanine nucleotide-exchange protein 1	0,427291904	0,00852017
C4B63_2g829	conserved hypothetical protein	0,427012686	0,003160363
C4B63_95g67	nucleoside 2-deoxyribosyltransferase	0,423210276	0,048604443
C4B63_480g19	cysteine peptidase	0,420835658	0,027610436
C4B63_353g3	unspecified product	0,418576924	0,023458273
C4B63_116g5	P-type H+-ATPase	0,418139012	0,006858805
C4B63_5g793	elongation factor 1-gamma (EF-1-gamma)	0,41813434	0,021004555
C4B63_211g35	conserved hypothetical protein	0,416046134	0,04066761
C4B63_361g15	trans-sialidase, Group V	0,414012068	0,012614008
C4B63_51g222	unspecified product	0,413551587	0,03207292
C4B63_56g94	conserved hypothetical protein	0,407488954	0,021422112
C4B63_46g107	NADH-cytochrome b5 reductase	0,406980431	0,030296037
C4B63_95g55	ubiquitin-like protein	0,406462525	0,040224268
C4B63_313g14	amino acid transporter	0,406412913	0,017533512
C4B63_28g151	formin-like protein	0,406343208	0,032356644
C4B63_41g155	cysteine peptidase	0,405634009	0,031837836
C4B63_89g66	conserved hypothetical protein	0,405541166	0,026304888
C4B63_38g160	ADP-ribosylation factor	0,40348227	0,047888596
C4B63_480g13	cysteine peptidase	0,400600565	0,020838019

Hb exclusive DEGs (t24 vs. t0)

Downregulated genes

ID	Annotation	log2FC	padj
C4B63_442g10	Uncharacterized protein C4orf22 homolog	-0,686055549	0,045677358
C4B63_3g1044	Mucin-associated surface protein (MASP)	-0,601749393	0,024298102
C4B63_83g44	retrotransposon hot spot (RHS) protein	-0,580620225	0,028925311
C4B63_85g422c	conserved hypothetical protein	-0,564153393	0,02035994
C4B63_39g97	dispersed gene family protein 1 (DGF-1)	-0,562213293	0,015648974
C4B63_77g33	conserved hypothetical protein	-0,542503306	0,010595508
C4B63_16g104	conserved hypothetical protein	-0,528548577	0,041076864
C4B63_30g183	conserved hypothetical protein	-0,517194945	0,02517909
C4B63_133g32	conserved hypothetical protein	-0,483531443	0,040327882
C4B63_134g54	trans-sialidase, Group V	-0,465816555	0,045323428
C4B63_5g29	surface protease GP63	-0,455920559	0,023235857
C4B63_40g21	Charged multivesicular body protein 3	-0,450412582	0,041057543
C4B63_47g60	conserved hypothetical protein	-0,442203732	0,004128351
C4B63_55g177	conserved hypothetical protein	-0,43775076	0,004058654
C4B63_41g249	conserved hypothetical protein	-0,429938038	0,013823208
C4B63_54g61	conserved hypothetical protein	-0,425155109	0,015648974
C4B63_70g99	conserved hypothetical protein	-0,421432874	0,042176862
C4B63_12g24	frataxin-like, mitochondrial precursor	-0,421387229	0,032168982
C4B63_4g135	conserved hypothetical protein	-0,408392016	0,022275137
C4B63_53g174	surface protease GP63	-0,403349858	0,008422994

Upregulated genes

ID	Annotation	log2FC	padj
C4B63_70g95	14-3-3 protein 2	0,94624206	0,000117864
C4B63_85g83	Eukaryotic initiation factor 4A-1	0,830748488	0,010598413
C4B63_46g16	conserved hypothetical protein	0,799189369	0,001720686
C4B63_46g17	conserved hypothetical protein	0,778783074	0,003470455
C4B63_46g18	conserved hypothetical protein	0,762512986	0,001486249
C4B63_31g80	Calcium ATPase SERCA-like	0,745740077	0,010256839
C4B63_15g18	NADH-dependent fumarate reductase	0,702636822	0,013823208
C4B63_12g330	ATP synthase subunit beta, mitochondrial	0,673431528	0,007787638
C4B63_114g34	conserved hypothetical protein	0,668635113	0,002105259
C4B63_148g35	zinc-finger protein 2	0,642550179	0,016088891
C4B63_61g93	conserved hypothetical protein	0,639931614	0,00149312
C4B63_2g507	zinc-finger protein	0,639767602	0,006296747
C4B63_20g172	chaperonin alpha subunit	0,635889887	0,022348217
C4B63_84g82	DNA mismatch repair protein MSH2	0,626637196	0,007986284
C4B63_4g90	ATP-dependent RNA helicase HEL67	0,623204622	0,033557941
C4B63_19g101	U-rich RNA-binding protein UBP-2	0,614614711	0,005218417
C4B63_135g38	retrotransposon hot spot (RHS) protein	0,605281209	0,02987447
C4B63_252g14	zinc-finger protein	0,603347938	0,00580587
C4B63_199g2	conserved hypothetical protein	0,596180794	0,033557941
C4B63_61g94	conserved hypothetical protein	0,5919266	0,036988403
C4B63_13g155	elongation factor 2	0,581084967	0,041615675
C4B63_20g178	dihydrolipoyl dehydrogenase	0,572910383	0,021388281
C4B63_13g154	elongation factor 2	0,570973797	0,028623579
C4B63_4g188	Elongation factor Tu, mitochondrial	0,567398678	0,000618794
C4B63_21g285	conserved hypothetical protein	0,555022963	0,040517462
C4B63_122g16	oligopeptidase b	0,549852681	0,020019923
C4B63_13g152	elongation factor 2	0,546847511	0,047628082
C4B63_13g153	elongation factor 2	0,546645343	0,041666099
C4B63_13g156	elongation factor 2	0,534903112	0,044108218
C4B63_13g157	elongation factor 2	0,511955196	0,039403081
C4B63_25g240	Macro domain containing protein	0,508154657	0,006709369
C4B63_72g79	inositol 5-phosphatase 1	0,496433928	0,010406012
C4B63_153g41	inositol 5-phosphatase 1(fragment)	0,483424401	0,0013735
C4B63_55g67	DNA-directed rna polymerase I largest subunit	0,482678336	0,000571227
C4B63_16g293	inositol 1,4,5-trisphosphate receptor	0,478986079	0,01683944
C4B63_54g186	retrotransposon hot spot (RHS) protein	0,47371581	0,036052354
C4B63_192g26	conserved hypothetical protein	0,47212596	0,046417241
C4B63_32g268	lysophospholipase	0,467707253	0,030478089
C4B63_138g113c	calmodulin	0,466976037	0,026426014
C4B63_153g40	inositol 5-phosphatase 1	0,46602356	0,021851252
C4B63_12g20	2-oxoglutarate-dependent dioxygenase tropC	0,463699866	0,029812791
C4B63_41g78	conserved hypothetical protein	0,460635701	0,012294855
C4B63_206g14	delta-1-pyrroline-5-carboxylate dehydrogenase	0,456909272	0,048959124
C4B63_57g65	membrane-bound acid phosphatase 2	0,456064019	0,019537051
C4B63_25g185	ATP-dependent RNA helicase	0,453036313	0,025024557
C4B63_133g42	dynein heavy chain	0,452789934	0,035326218
C4B63_89g45	Mitochondrial SSU ribosomal protein 29	0,451945363	0,015648974
C4B63_61g92	conserved hypothetical protein	0,446102262	0,001720686
C4B63_21g42	PAB1-binding protein	0,44188437	0,041439579
C4B63_42g220	transmembrane/endomembrane-like protein	0,440905941	0,046614419
C4B63_12g195	conserved hypothetical protein	0,438398571	0,006111694
C4B63_61g95	conserved hypothetical protein	0,437214353	0,0065861
C4B63_25g237	eukaryotic translation initiation factor 3 subunit b	0,435441248	0,007152993
C4B63_13g344	conserved hypothetical protein	0,43484463	0,030296061
C4B63_34g110	mitochondrial carrier protein	0,426491385	0,032866209
C4B63_26g260	solanesyl-diphosphate synthase	0,423504918	0,01095188
C4B63_6g223	DNA topoisomerase II	0,410844609	0,041888784
C4B63_31g104	conserved hypothetical protein	0,410047997	0,001786674
C4B63_38g167	spermidine synthase	0,406170396	0,018998446
C4B63_134g37	orotidine-5-phosphate decarboxylase/orotate phosphoribosyltra	0,405015126	0,035326218
C4B63_35g109	conserved hypothetical protein	0,403130009	0,031563786

No heme exclusive DEGs (t4 vs. t0)

Downregulated genes

ID	Annotation	log2FC	padj
C4B63_101g1	tyrosine aminotransferase	-0,7288	0,00511
C4B63_101g2	tyrosine aminotransferase	-0,7178	0,00323
C4B63_101g3	tyrosine aminotransferase	-0,7669	0,00101
C4B63_101g4	tyrosine aminotransferase	-0,7288	0,00255
C4B63_101g5	tyrosine aminotransferase	-0,7178	0,00072
C4B63_13g199	GPI transamidase component Tta2	-0,7669	0,02806
C4B63_21g103	citrate transporter	-0,4562	0,04182
C4B63_25g331	trans-sialidase, Group I	-1,0142	0,02476
C4B63_26g354	tyrosine aminotransferase	-0,7442	0,00196
C4B63_33g163	unspecified product	-0,4737	0,01724
C4B63_33g164	unspecified product	-0,5137	0,04286
C4B63_42g164	tyrosine aminotransferase	-0,7621	0,00150
C4B63_43g196	adenylate kinase 1	-0,5080	0,04341
C4B63_44g227	amino acid permease	-0,5240	0,00100
C4B63_4g336	CMP-sialic acid transporter	-0,4944	0,04398
C4B63_54g138	tyrosine aminotransferase	-0,7707	0,00046
C4B63_81g64	tyrosine aminotransferase	-0,7436	0,00191
C4B63_81g65	tyrosine aminotransferase	-0,6865	0,00247
C4B63_81g66	tyrosine aminotransferase	-0,7133	0,00250
C4B63_81g67	tyrosine aminotransferase	-0,7029	0,00327
C4B63_81g68	tyrosine aminotransferase	-0,7169	0,00144
C4B63_81g69	tyrosine aminotransferase	-0,7204	0,00118
C4B63_81g70	tyrosine aminotransferase	-0,7579	0,00450

Upregulated genes

ID	Annotation	log2FC	padj
C4B63_10g1811c	cofilin/actin depolymerizing factor	0,4488	0,0133
C4B63_20g177	chaperone DNAJ protein	0,4812	0,0010
C4B63_23g234	inosine-adenosine-guanosine-nucleosidehydrolase	0,4485	0,0033
C4B63_252g15	arginyl-tRNA synthetase	0,4827	0,0343
C4B63_253g15	haloacid dehalogenase-like hydrolase	0,4921	0,0329
C4B63_45g221	ATP-dependent DEAD/H RNA helicase	0,4464	0,0272
C4B63_51g80	ferric reductase transmembrane protein	0,4453	0,0077
C4B63_51g81	ferric reductase transmembrane protein	0,5492	0,0007
C4B63_51g82	ferric reductase transmembrane protein	0,4628	0,0048
C4B63_51g83	ferric reductase transmembrane protein	0,4835	0,0121
C4B63_51g85	ferric reductase transmembrane protein	0,5001	0,0026
C4B63_61g73	Acyl-CoA-binding domain-containing protein 5	0,5059	0,0146
C4B63_76g32	guide RNA associated protein, GAP2	0,4001	0,0385

No heme exclusive DEGs (t24 vs. t0)

Downregulated genes

ID	Annotation	log2FC	padj
C4B63_122g15	conserved hypothetical protein	-0,6850	0,0487
C4B63_127g52	serine/threonine-protein kinase A(fragment)	-0,4436	0,0034
C4B63_135g21	ascorbate peroxidase	-1,0347	0,0174
C4B63_13g200	Enriched in surface-labeled proteome protein 18	-0,7645	0,0008
C4B63_181g25	conserved hypothetical protein	-0,9024	0,0293
C4B63_22g121	unspecified product	-0,6816	0,0215
C4B63_248g12	Present in the outer mitochondrial membrane proteom	-0,7129	0,0470
C4B63_267g18	conserved hypothetical protein	-0,5594	0,0430
C4B63_26g51	Mucin-associated surface protein (MASP)	-0,6166	0,0407
C4B63_281g9	Ring finger domain containing protein	-0,6889	0,0411
C4B63_28g96	sugar transporter(fragment)	-0,7890	0,0407
C4B63_33g147	NADH-ubiquinone oxidoreductase, mitochondrial	-0,7577	0,0152
C4B63_38g176	conserved hypothetical protein	-0,6967	0,0337
C4B63_43g134	cytochrome c oxidase subunit VI	-0,5408	0,0060
C4B63_43g98	unspecified product	-0,6609	0,0408
C4B63_45g115	Protein kinase domain	-0,4218	0,0484
C4B63_47g23	ascorbate peroxidase	-0,9819	0,0364
C4B63_4g189	alcohol dehydrogenase	-0,9326	0,0020
C4B63_55g92	conserved hypothetical protein	-0,4839	0,0294
C4B63_6g259	nucleotidyl transferase	-0,4357	0,0145
C4B63_7g361	phosphomannomutase-like protein	-0,6676	0,0144
C4B63_82g448c	conserved hypothetical protein	-0,4751	0,0355
C4B63_89g64	hydroxyacylglutathione hydrolase	-0,5887	0,0398
C4B63_9g408	conserved hypothetical protein	-0,5091	0,0246

Upregulated genes

ID	Annotation	log2FC	padj
C4B63_18g39	CRAL/TRIO, N-terminal domain/CRAL/TRIO domai	0,6361	0,0011
C4B63_23g43	conserved hypothetical protein	0,5146	0,0261
C4B63_28g80	DnaJ chaperone protein	0,4633	0,0421
C4B63_302g66c	histone H3	0,7388	0,0394
C4B63_30g161	glutamine synthetase	0,4201	0,0474
C4B63_51g81	ferric reductase transmembrane protein	0,4310	0,0171
C4B63_51g82	ferric reductase transmembrane protein	0,4176	0,0171
C4B63_6g2538c	histone H3	0,7169	0,0407
C4B63_6g2542c	histone H3	0,6173	0,0355
C4B63_6g2554c	histone H3	0,6934	0,0322
C4B63_6g2562c	histone H3	0,8380	0,0101

Gene Ontology analysis of Hemin exclusive DEGs (downregulated, t4 vs. t0)									
	**CELLULAR COMPONENT**								
ID	Name	Bgd count	Result count	Result gene	Pct of bgd	Fold enrichm	Odds ratio	P-value	Benjamini	Bonferroni
GO:0042995	cell projection	163	31	C4B63_231g	19.0	13.25	21.22	6,73E-27	9,26E-26	3,70E-25
GO:0043227	membrane-bounded organelle	995	34	C4B63_231g	3.4	2.38	2.97	8,15E-07	8,97E-06	4,48E-05
	**MOLECULAR FUNCTION**									
ID	Name	Bgd count	Result count	Result gene	Pct of bgd	Fold enrichm	Odds ratio	P-value	Benjamini	Bonferroni
GO:0005516	calmodulin binding	24	11	C4B63_55g3	45.8	31.92	63.7	7,24E-15	1,01E-12	1,01E-12
GO:0046872	metal ion binding	792	31	C4B63_13g2	3.9	2.73	3.4	1,49E-07	6,69E-06	2,07E-05

Gene Ontology analysis of Hemin exclusive DEGs (upregulated, t4 vs. t0)										
	**MOLECULAR FUNCTION**									
ID	Name	Bgd count	Result count	Result gene	Pct of bgd	Fold enrichm	Odds ratio	P-value	Benjamini	Bonferroni
GO:0003723	RNA binding	425	18	C4B63_109g	4.2	3.05	3.52	2,07E-05	0.00394570728267	0.00394570728267
GO:0003676	nucleic acid binding	987	29	C4B63_109g	2.9	2.12	2.52	5,98E-05	0.00571378513375	0.0114275702675

Gene Ontology analysis of Hemin exclusive DEGs (downregulated, t24 vs. t0)										
	**CELLULAR COMPONENT**									
ID	Name	Bgd count	Result count	Result gene	Pct of bgd	Fold enrichm	Odds ratio	P-value	Benjamini	Bonferroni
GO:0016020	membrane	2776	85	C4B63_109g	3.1	1.51	2.02	3,94E-06	6,70E-05	0.000200902193868
	**MOLECULAR FUNCTION**									
ID	Name	Bgd count	Result count	Result gene	Pct of bgd	Fold enrichm	Odds ratio	P-value	Benjamini	Bonferroni
GO:0030983	mismatched DNA binding	16	6	C4B63_102g	37.5	18.46	29.94	4,36E-07	5,58E-05	5,58E-05
GO:0003690	double-stranded DNA binding	19	6	C4B63_102g	31.6	15.55	23.02	1,40E-06	8,98E-05	0.000179602487712
GO:0016997	alpha-sialidase activity	711	31	C4B63_109g	4.4	2.15	2.46	3,49E-05	0.00111708551683	0.00446834206732

Gene Ontology analysis of Hemin exclusive DEGs (upregulated, t24 vs. t0)										
	**BIOLOGICAL PROCESS**									
ID	Name	Bgd count	Result count	Result gene	Pct of bgd	Fold enrichm	Odds ratio	P-value	Benjamini	Bonferroni
GO:0019752	carboxylic acid metabolic process	307	19	C4B63_12g2	6.2	3.27	3.74	4,90E-06	0.000399771837744	0.00223393647037
GO:0006096	glycolytic process	22	5	C4B63_13g2	22.7	12.02	15.71	4,61E-05	0.00140249904726	0.0210374857089
GO:1901360	organic cyclic compound metabolic process	722	28	C4B63_10g4	3.9	2.05	2.32	0.000184754098715	0.00350964222004	0.0842478690139
GO:0008610	lipid biosynthetic process	85	8	C4B63_123g	9.4	4.98	5.62	0.000192414595397	0.00350964222004	0.0877410555011
GO:0043039	tRNA aminoacylation	65	7	C4B63_297g	10.8	5.7	6.5	0.000210465679428	0.00355453147479	0.0959723498193
	**CELLULAR COMPONENT**									
ID	Name	Bgd count	Result count	Result gene	Pct of bgd	Fold enrichm	Odds ratio	P-value	Benjamini	Bonferroni
GO:0005634	nucleus	424	21	C4B63_112g	5.0	2.62	2.96	4,59E-05	0.00140020490363	0.00560081961453
GO:0044815	DNA packaging complex	124	10	C4B63_128g	8.1	4.26	4.78	0.000113442888295	0.00221903678164	0.013840032372
GO:0030137	COPI-coated vesicle	7	3	C4B63_22g1	42.9	22.66	39.63	0.000219718471203	0.00297840594297	0.0268056534868
GO:0020015	glycosome	2	2	C4B63_18g2	100.0	52.88	inf	0.000355413609349	0.00309717573861	0.0433604603406
GO:0005798	Golgi-associated vesicle	9	3	C4B63_22g1	33.3	17.63	26.41	0.000512796726598	0.00368007062618	0.062561200645
GO:0005737	cytoplasm	813	29	C4B63_112g	3.6	1.89	2.12	0.000571835754917	0.00373538103579	0.0697639620999
GO:0032991	protein-containing complex	630	24	C4B63_10g5	3.8	2.01	2.24	0.000726724781263	0.0044330211657	0.0886604233141
	**MOLECULAR FUNCTION**									
ID	Name	Bgd count	Result count	Result gene	Pct of bgd	Fold enrichm	Odds ratio	P-value	Benjamini	Bonferroni
GO:1901363	heterocyclic compound binding	2258	66	C4B63_10g4	2.9	1.55	1.95	4,07E-05	0.00476664764343	0.00953329528686
GO:0016705	oxidoreductase activity, acting on paired donors, v	14	4	C4B63_123g	28.6	15.11	21.25	0.000106378177048	0.00711262363991	0.0248924934292
GO:0005524	ATP binding	1110	37	C4B63_113g	3.3	1.76	2.02	0.000336855217016	0.00711262363991	0.0788241207818
GO:0004332	fructose-bisphosphate aldolase activity	2	2	C4B63_13g2	100.0	52.88	inf	0.000355413609349	0.00711262363991	0.0831667845877
GO:0050242	pyruvate, phosphate dikinase activity	2	2	C4B63_12g2	100.0	52.88	inf	0.000355413609349	0.00711262363991	0.0831667845877

Gene Ontology analysis of Hb exclusive DEGs (downregulated, t4 vs. t0)										
	**CELLULAR COMPONENT**									
ID	Name	Bgd count	Result count	Result gene	Pct of bgd	Fold enrichm	Odds ratio	P-value	Benjamini	Bonferroni
GO:0005739	mitochondrion	131	9	C4B63_12g2	6.9	6.07	7.0	1,59E-05	0.00047775	0.000955550395816
	**MOLECULAR FUNCTION**									
ID	Name	Bgd count	Result count	Result gene	Pct of bgd	Fold enrichm	Odds ratio	P-value	Benjamini	Bonferroni
GO:0016491	oxidoreductase activity	402	19	C4B63_101g	4.7	4.17	5.14	8,89E-08	1,30E-05	1,30E-05
GO:0004435	phosphatidylinositol phospholipase C activity	2	2	C4B63_120g	100.0	88.32	inf	0.00012689	0.00463157	0.0185263125304

Gene Ontology analysis of Hb exclusive DEGs (upregulated, t4 vs. t0)										
	**BIOLOGICAL PROCESS**									
ID	Name	Bgd count	Result count	Result gene	Pct of bgd	Fold enrichm	Odds ratio	P-value	Benjamini	Bonferroni
GO:0006508	proteolysis	318	31	C4B63_16g1	9.7	9.18	14.74	2,86E-22	4,57E-20	4,57E-20
GO:0008380	RNA splicing	63	5	C4B63_178g	7.9	7.47	8.44	0.00052373	0.00761794	0.0837973837498
	**CELLULAR COMPONENT**									
ID	Name	Bgd count	Result count	Result gene	Pct of bgd	Fold enrichm	Odds ratio	P-value	Benjamini	Bonferroni
GO:0044815	DNA packaging complex	124	15	C4B63_188g	12.1	11.39	15.15	2,36E-12	2,52E-11	8,96E-11
GO:0005634	nucleus	424	15	C4B63_10g1	3.5	3.33	3.89	3,51E-05	0.00019062	0.00133437379986
	**MOLECULAR FUNCTION**									
ID	Name	Bgd count	Result count	Result gene	Pct of bgd	Fold enrichm	Odds ratio	P-value	Benjamini	Bonferroni
GO:0003676	nucleic acid binding	987	24	C4B63_10g1	2.4	2.29	2.79	6,70E-05	0.00064231	0.0077077624258

Gene Ontology analysis of Hb exclusive DEGs (downregulated, t24 vs. t0)										
	**MOLECULAR FUNCTION**									
ID	Name	Bgd count	Result count	Result gene	Pct of bgd	Fold enrichm	Odds ratio	P-value	Benjamini	Bonferroni
GO:0008199	ferric iron binding	1	1	C4B63_12g2	100.0	778.82	inf	0.00128399	0.00770398	0.0308159215598

Gene Ontology analysis of Hb exclusive DEGs (upregulated, t24 vs. t0)										
	**BIOLOGICAL PROCESS**									
ID	Name	Bgd count	Result count	Result gene	Pct of bgd	Fold enrichm	Odds ratio	P-value	Benjamini	Bonferroni
GO:0006816	calcium ion transport	5	2	C4B63_16g2	40.0	76.15	132.08	0.00026711	0.02555691	0.0512853154451
GO:0046856	phosphatidylinositol dephosphorylation	6	2	C4B63_153g	33.3	63.46	99.05	0.00039932	0.02555691	0.07667075578
	**MOLECULAR FUNCTION**									
ID	Name	Bgd count	Result count	Result gene	Pct of bgd	Fold enrichm	Odds ratio	P-value	Benjamini	Bonferroni
GO:0045182	translation regulator activity	128	9	C4B63_13g1	7.0	13.39	17.65	1,57E-08	6,19E-07	1,86E-06
GO:0003676	nucleic acid binding	987	15	C4B63_13g1	1.5	2.89	3.88	9,13E-05	0.00098822	0.0107720144924
GO:0043167	ion binding	2086	23	C4B63_12g2	1.1	2.1	3.27	9,21E-05	0.00098822	0.0108705236749

Gene Ontology analysis of No heme exclusive DEGs (downregulated, t4 vs. t0)										
	**BIOLOGICAL PROCESS**									
ID	Name	Bgd count	Result count	Result gene	Pct of bgd	Fold enrichm	Odds ratio	P-value	Benjamini	Bonferroni
GO:0009072	aromatic amino acid family metabolic process	13	11	C4B63_101g	84.6	362.45	5221.94	2,88E-29	1,80E-28	1,44E-27
	**CELLULAR COMPONENT**									
ID	Name	Bgd count	Result count	Result gene	Pct of bgd	Fold enrichm	Odds ratio	P-value	Benjamini	Bonferroni
GO:0005739	mitochondrion	131	9	C4B63_101g	6.9	29.43	56.5	5,05E-12	4,55E-11	9,09E-11
GO:0005737	cytoplasm	813	10	C4B63_101g	1.2	5.27	9.64	4,28E-06	2,57E-05	7,70E-05
	**MOLECULAR FUNCTION**									
ID	Name	Bgd count	Result count	Result gene	Pct of bgd	Fold enrichm	Odds ratio	P-value	Benjamini	Bonferroni
GO:0008483	transaminase activity	25	15	C4B63_101g	60.0	257.01	2561.1	6,79E-37	1,46E-35	2,92E-35
GO:0030170	pyridoxal phosphate binding	36	15	C4B63_101g	41.7	178.48	1218.0	1,15E-33	1,24E-32	4,94E-32

Gene Ontology analysis of No heme exclusive DEGs (upregulated, t4 vs. t0)										
	**BIOLOGICAL PROCESS**									
ID	Name	Bgd count	Result count	Result gene	Pct of bgd	Fold enrichm	Odds ratio	P-value	Benjamini	Bonferroni
GO:0030042	actin filament depolymerization	1	1	C4B63_10g1	100.0	713.92	inf	0.00140072370725	0.0280144741449	0.0840434224348
	**MOLECULAR FUNCTION**									
ID	Name	Bgd count	Result count	Result gene	Pct of bgd	Fold enrichm	Odds ratio	P-value	Benjamini	Bonferroni
GO:0016491	oxidoreductase activity	402	5	C4B63_51g8	1.2	8.88	14.68	0.000133649842961	0.00574694324732	0.00574694324732

Gene Ontology analysis of No heme exclusive DEGs (downregulated, t24 vs. t0)										
	**BIOLOGICAL PROCESS**									
ID	Name	Bgd count	Result count	Result gene	Pct of bgd	Fold enrichm	Odds ratio	P-value	Benjamini	Bonferroni
GO:0006979	response to oxidative stress	11	2	C4B63_135g	18.2	119.82	172.63	0.000116019957952	0.00359661869651	0.00359661869651
GO:0008643	carbohydrate transport	2	1	C4B63_28g9	50.0	329.5	712.75	0.00303277558837	0.0313386810798	0.0940160432395
	**CELLULAR COMPONENT**									
ID	Name	Bgd count	Result count	Result gene	Pct of bgd	Fold enrichm	Odds ratio	P-value	Benjamini	Bonferroni
GO:0070469	respirasome	15	2	C4B63_33g1	13.3	87.87	119.45	0.000220735212019	0.0057391155125	0.0057391155125
	**MOLECULAR FUNCTION**									
ID	Name	Bgd count	Result count	Result gene	Pct of bgd	Fold enrichm	Odds ratio	P-value	Benjamini	Bonferroni
GO:0004601	peroxidase activity	17	2	C4B63_135g	11.8	77.53	103.5	0.000285415414571	0.0044914450695	0.015983263216
GO:0043169	cation binding	801	6	C4B63_135g	0.7	4.94	8.37	0.000632667764084	0.00541458438806	0.0354293947887

Gene Ontology analysis of No heme exclusive DEGs (upregulated, t24 vs. t0)										
	**BIOLOGICAL PROCESS**									
ID	Name	Bgd count	Result count	Result gene	Pct of bgd	Fold enrichm	Odds ratio	P-value	Benjamini	Bonferroni
GO:0006542	glutamine biosynthetic process	2	1	C4B63_30g1	50.0	475.94	1069.62	0.00210010443286	0.05880292412	0.05880292412
	**CELLULAR COMPONENT**									
ID	Name	Bgd count	Result count	Result gene	Pct of bgd	Fold enrichm	Odds ratio	P-value	Benjamini	Bonferroni
GO:0044815	DNA packaging complex	124	5	C4B63_302g	4.0	38.38	88.64	7,05E-08	2,94E-07	1,13E-06
GO:0000785	chromatin	125	5	C4B63_302g	4.0	38.08	87.9	7,34E-08	2,94E-07	1,17E-06
	**MOLECULAR FUNCTION**									
ID	Name	Bgd count	Result count	Result gene	Pct of bgd	Fold enrichm	Odds ratio	P-value	Benjamini	Bonferroni
GO:0003677	DNA binding	291	5	C4B63_302g	1.7	16.36	36.15	4,92E-06	2,63E-05	7,88E-05

Table S4. List and Gene Ontology analysis of DEGs exclusively modulated in hemin, Hb or no heme conditions in the t_4 vs. t_0 and t_{24} vs. t_0 analysis. Highlighted in yellow: genes probably involved in signaling pathways.

Table S5

Hemin DEGs (t4 vs. t0)∩(t24 vs. t0)			
Downregulated		Upregulated	
ID	Annotation	ID	Annotation
C4B63_10g453	ABC transporter	C4B63_10g436	SPFH domain / Band 7 family
C4B63_10g454	ABC transporter(fragment)	C4B63_111g29	conserved hypothetical protein
C4B63_10g455	ABC transporter	C4B63_114g17	conserved hypothetical protein
C4B63_10g472	conserved hypothetical protein	C4B63_114g25	conserved hypothetical protein
C4B63_113g47	Generative cell specific 1 protein	C4B63_116g15	membrane transporter protein
C4B63_113g62	kinetoplast DNA-associated protein	C4B63_11g63	D-alanine--D-alanine ligase
C4B63_127g46	conserved hypothetical protein	C4B63_133g1	NAD-specific glutamate dehydrogenase
C4B63_12g151	conserved hypothetical protein	C4B63_137g10	Coatomer subunit gamma
C4B63_134g49	trans-sialidase, Group V	C4B63_142g6	GMP synthase
C4B63_134g59	trans-sialidase, Group V	C4B63_143g34	condensin complex subunit 2
C4B63_13g146	conserved hypothetical protein	C4B63_14g39	conserved hypothetical protein
C4B63_13g208	conserved hypothetical protein,zinc finger protein family member	C4B63_151g36	notchless homolog
C4B63_13g223	zinc-finger protein family member	C4B63_15g12	conserved hypothetical protein
C4B63_13g256	conserved hypothetical protein	C4B63_15g16	oligosaccharyl transferase subunit
C4B63_13g317	vesicle-associated membrane protein	C4B63_171g4	phosphatidic acid phosphatase protein
C4B63_14g101	conserved hypothetical protein	C4B63_183g14	conserved hypothetical protein
C4B63_14g158	serine/threonine kinase	C4B63_184g8	GTP-binding protein
C4B63_14g20	hexose transporter	C4B63_18g152	conserved hypothetical protein
C4B63_14g43	centrin-3	C4B63_19g205	glycosomal transporter (GAT3)
C4B63_156g3	unspecified product	C4B63_19g82	RNA polymerase I second largest subunit
C4B63_156g4	unspecified product	C4B63_1g57	AAA ATPase
C4B63_157g36	trans-sialidase, Group II	C4B63_1g70	mitochondrial RNA-binding protein 2
C4B63_16g183	surface protease GP63	C4B63_200g32	3,2-trans-enoyl-CoA isomerase, mitochondrial precursor
C4B63_16g93	Uncharacterized protein in mobD 3'region	C4B63_20g747c	cyclin-dependent kinases regulatory subunit
C4B63_17g196	calpain-like cysteine peptidase	C4B63_210g33	p-nitrophenylphosphatase
C4B63_17g61	conserved hypothetical protein	C4B63_220g11	Beta propeller protein 1
C4B63_180g10	retrotransposon hot spot (RHS) protein	C4B63_227g7	transporter
C4B63_18g308	sphingosine 1-phosphate lyase	C4B63_227g9	mevalonate-diphosphate decarboxylase
C4B63_18g322	conserved hypothetical protein	C4B63_23g116	Importin beta-like SAD2
C4B63_18g326	zinc-finger protein ZFP1	C4B63_252g23	Serine-threonine kinase receptor-associated protein
C4B63_18g330	zinc-finger protein ZFP1	C4B63_25g113	GMP reductase
C4B63_18g39	CRAL/TRIO, N-terminal domain/CRAL/TRIO domain containing protein	C4B63_25g117	conserved hypothetical protein
C4B63_196g33	unspecified product	C4B63_25g234	RNA cytidine acetyltransferase
C4B63_19g141	electron transfer flavoprotein	C4B63_263g7	ruvB-like DNA helicase
C4B63_19g257	conserved hypothetical protein	C4B63_282g15	cation transporter
C4B63_1g473	trans-sialidase	C4B63_286g18	eukaryotic translation initiation factor 3 subunit c
C4B63_1g480	trans-sialidase, Group V	C4B63_28g180	WASH complex subunit 4
C4B63_1g702	trans-sialidase, Group V	C4B63_28g56	TFIIF-stimulated CTD phosphatase
C4B63_20g203	Zinc finger protein, C3H1 type-like	C4B63_2g209	Present in the outer mitochondrial membrane proteome 6
C4B63_21g27	arginine permease(fragment)	C4B63_2g219	conserved hypothetical protein
C4B63_21g6	arginine permease	C4B63_2g289	Hypoxia up-regulated protein 1 (Fragment)
C4B63_244g8	trans-sialidase, Group I	C4B63_2g331	polyadenylate-binding protein 1
C4B63_24g194	microtubule-associated protein	C4B63_2g43	short-chain dehydrogenase
C4B63_24g236	microtubule-associated protein Gb4	C4B63_2g486	Serine-threonine kinase receptor-associated protein
C4B63_24g274	conserved hypothetical protein	C4B63_322g14	amino acid transporter
C4B63_24g275	conserved hypothetical protein	C4B63_35g130	orotidine-5-phosphate decarboxylase/orotate phosphoribosyltransferase
C4B63_24g313	protein kinase	C4B63_35g168	3-Beta-hydroxysteroid-delta(8), delta(7)-isomerase
C4B63_251g10	lipase	C4B63_38g147	spermidine synthase
C4B63_258g25	lipase	C4B63_396g14	Present in the outer mitochondrial membrane proteome 6
C4B63_25g207	hydrolase-like protein	C4B63_40g159	translation initiation factor 2 subunit
C4B63_25g220	5'-3' exoribonuclease B	C4B63_40g25	Biotin--acetyl-CoA-carboxylase ligase
C4B63_25g236	conserved hypothetical protein	C4B63_41g139	conserved hypothetical protein
C4B63_26g310	ABC transporter	C4B63_45g97	eukaryotic translation initiation factor 3 subunit h
C4B63_26g34	mucin TcMUCII	C4B63_4g118	conserved hypothetical protein
C4B63_277g19	metallo-peptidase, clan MH, family M18	C4B63_4g144	protein transport protein SEC13
C4B63_278g3	conserved hypothetical protein	C4B63_4g219	nucleolar RNA-associated proteins (Nrap)
C4B63_27g1020c	RNA-binding protein	C4B63_4g381	transporter
C4B63_27g184	integral membrane transport protein	C4B63_4g383	mevalonate-diphosphate decarboxylase
C4B63_294g3	metallo-peptidase, clan MH, family M18	C4B63_4g72	fibrillarin
C4B63_2g156	protein kinase A catalytic subunit 3	C4B63_51g842c	Uncharacterized protein At2g34160
C4B63_2g309	NIMA-related kinase	C4B63_52g161	tyrosyl-tRNA synthetase
C4B63_30g222	conserved hypothetical protein	C4B63_52g28	catalytic subunit of the vacuolar transporter chaperone 4(fragment)
C4B63_322g4	glutamamyl carboxypeptidase(fragment)	C4B63_52g54	conserved hypothetical protein
C4B63_323g3	unspecified product	C4B63_56g31	conserved hypothetical protein
C4B63_354g1	electron transfer flavoprotein	C4B63_57g98	succinyl-CoA ligase
C4B63_37g189	trans-sialidase, Group VI	C4B63_61g149	eukaryotic translation initiation factor 3 subunit a
C4B63_3g1092	alanine racemase	C4B63_63g145	retrotransposon hot spot (RHS) protein
C4B63_419g2	trans-sialidase	C4B63_63g7	eukaryotic translation initiation factor 3 subunit c
C4B63_43g74	retrotransposon hot spot (RHS) protein	C4B63_6g161	Eukaryotic initiation factor 4A-1
C4B63_44g183	conserved hypothetical protein	C4B63_6g19	NAD(P)-dependent steroid dehydrogenase protein
C4B63_44g231	amino acid transporter	C4B63_6g236	cation transporter
C4B63_44g235	Kinesin-13 4	C4B63_6g397	cation transporter
C4B63_44g243	conserved hypothetical protein	C4B63_6g562	NAD(P)-dependent steroid dehydrogenase protein
C4B63_459g4	conserved hypothetical protein	C4B63_70g104	Ribosome biogenesis protein BOP1 homolog
C4B63_45g139	serine/threonine protein kinase	C4B63_73g16	conserved hypothetical protein
C4B63_462g9	glutamamyl carboxypeptidase(fragment)	C4B63_79g20	conserved hypothetical protein
C4B63_47g122	trans-sialidase	C4B63_79g21	ABC transporter
C4B63_47g125	trans-sialidase	C4B63_79g37	methionyl-tRNA synthetase
C4B63_48g693c	conserved hypothetical protein	C4B63_7g266	conserved hypothetical protein
C4B63_49g168	conserved hypothetical protein	C4B63_7g316	ruvB-like DNA helicase
C4B63_4g132	NLI interacting factor-like phosphatase	C4B63_7g318	peroxisome biogenesis factor 1
C4B63_4g180	serine/threonine protein kinase	C4B63_7g340	beta-fructofuranosidase-like protein
C4B63_4g242	protein kinase A catalytic subunit 3	C4B63_7g347	V-type ATPase, A subunit
C4B63_4g283	conserved hypothetical protein	C4B63_80g17	ATP binding protein-like protein
C4B63_4g511	zinc-finger protein	C4B63_81g17	DNA mismatch repair protein MSH2(fragment)
C4B63_4g545	RNA-binding protein 4	C4B63_81g18	chaperonin HSP60, mitochondrial precursor(fragment)
C4B63_52g153	conserved hypothetical protein	C4B63_84g49	RNA-binding protein
C4B63_53g165	conserved hypothetical protein	C4B63_84g67	conserved hypothetical protein
C4B63_550g4	glutamamyl carboxypeptidase(fragment)	C4B63_86g57	conserved hypothetical protein
C4B63_550g5	glutamamyl carboxypeptidase(fragment)	C4B63_8g510	conserved hypothetical protein
C4B63_58g126	ubiquitin-conjugating enzyme variant Kua homologue	C4B63_8g62	Nucleolar protein 10
C4B63_59g207	mucin TcMUCII	C4B63_97g19	pyruvate dehydrogenase E1 component alpha subunit
C4B63_5g393	trans-sialidase, Group V	C4B63_97g34	P-type H+-ATPase
C4B63_65g5	unspecified product	C4B63_97g36	P-type H+-ATPase
C4B63_66g170	Mucin-associated surface protein (MASP)	C4B63_97g39	P-type H+-ATPase
C4B63_66g61	surface protease GP63	C4B63_98g4	glucose-6-phosphate 1-dehydrogenase
C4B63_66g68	trans-sialidase, Group V	C4B63_9g201	GTP-binding protein
C4B63_69g318c	conserved hypothetical protein		
C4B63_6g284	cytochrome c oxidase assembly protein		
C4B63_6g587	N-glycosidase YbiA		
C4B63_70g82	protein kinase		
C4B63_70g84	serine/threonine protein kinase		
C4B63_76g17	conserved hypothetical protein		
C4B63_76g66	conserved hypothetical protein		
C4B63_89g19	retrotransposon hot spot (RHS) protein		
C4B63_8g495	Regulator of chromosome condensation (RCC1) repeat		
C4B63_92g110	protein associated with differentiation 8		
C4B63_96g38	trans-sialidase, Group V		
C4B63_97g13	conserved hypothetical protein		
C4B63_97g15	Zinc finger CCCH domain-containing protein 35		
C4B63_99g112	alanine racemase		
C4B63_9g234	dolichyl-P-Man:GDP-Man7GlcNAc2-PP-dolichyl alpha-1,6-mannosyltransferase		
C4B63_9g376	Present in the outer mitochondrial membrane proteome 22		
C4B63_9g377	Present in the outer mitochondrial membrane proteome 22		
C4B63_9g383	conserved hypothetical protein		
C4B63_9g387	conserved hypothetical protein		

Hb DEGs (t4 vs t0)∩(t24 vs t0)			
Downregulated		Upregulated	
ID	Annotation	ID	Annotation
C4B63_113g47	Generative cell specific 1 protein	C4B63_10g436	SPFH domain / Band 7 family
C4B63_12g24	frataxin-like, mitochondrial precursor	C4B63_111g29	conserved hypothetical protein
C4B63_133g32	conserved hypothetical protein	C4B63_114g34	conserved hypothetical protein
C4B63_134g49	trans-sialidase, Group V	C4B63_122g16	oligopeptidase b
C4B63_134g54	trans-sialidase, Group V	C4B63_134g37	orotidine-5-phosphate decarboxylase/orotate phosphoribosyltransferase
C4B63_13g146	conserved hypothetical protein	C4B63_13g152	elongation factor 2
C4B63_13g317	vesicle-associated membrane protein	C4B63_13g153	elongation factor 2
C4B63_16g104	conserved hypothetical protein	C4B63_13g154	elongation factor 2
C4B63_19g130	Dual specificity phosphatase, catalytic domain containing protein	C4B63_13g155	elongation factor 2
C4B63_1g480	trans-sialidase, Group V	C4B63_13g156	elongation factor 2
C4B63_21g11	arginine permease	C4B63_13g157	elongation factor 2
C4B63_21g27	arginine permease(fragment)	C4B63_142g6	GMP synthase
C4B63_244g7	trans-sialidase, Group I	C4B63_15g12	conserved hypothetical protein
C4B63_24g313	protein kinase	C4B63_15g15	oligosaccharyl transferase subunit
C4B63_2g309	NIMA-related kinase	C4B63_15g16	oligosaccharyl transferase subunit
C4B63_30g222	conserved hypothetical protein	C4B63_16g293	inositol 1,4,5-trisphosphate receptor
C4B63_3g1089	Alpha-ketoglutarate-dependent dioxygenase AlkB-like superfamily	C4B63_188g156c	histone H4
C4B63_3g1092	alanine racemase	C4B63_192g26	conserved hypothetical protein
C4B63_41g249	conserved hypothetical protein	C4B63_19g101	U-rich RNA-binding protein UBP-2
C4B63_442g10	Uncharacterized protein C4orf22 homolog	C4B63_1g57	AAA ATPase
C4B63_47g60	conserved hypothetical protein	C4B63_200g32	3,2-trans-enoyl-CoA isomerase, mitochondrial precursor
C4B63_49g168	conserved hypothetical protein	C4B63_23g116	Importin beta-like SAD2
C4B63_4g180	serine/threonine protein kinase	C4B63_25g234	RNA cytidine acetyltransferase
C4B63_70g65	conserved hypothetical protein	C4B63_25g237	eukaryotic translation initiation factor 3 subunit b
C4B63_77g33	conserved hypothetical protein	C4B63_25g240	Macro domain containing protein
C4B63_9g387	conserved hypothetical protein	C4B63_2g209	Present in the outer mitochondrial membrane proteome 6
		C4B63_35g168	3-Beta-hydroxysteroid-delta(8), delta(7)-isomerase
		C4B63_396g14	Present in the outer mitochondrial membrane proteome 6
		C4B63_40g87	WD40 repeat-containing protein
		C4B63_41g78	conserved hypothetical protein
		C4B63_46g16	conserved hypothetical protein
		C4B63_46g17	conserved hypothetical protein
		C4B63_47g73	importin beta-1 subunit
		C4B63_55g67	DNA-directed rna polymerase I largest subunit
		C4B63_57g65	membrane-bound acid phosphatase 2
		C4B63_61g149	eukaryotic translation initiation factor 3 subunit a
		C4B63_61g92	conserved hypothetical protein
		C4B63_61g93	conserved hypothetical protein
		C4B63_61g94	conserved hypothetical protein
		C4B63_61g95	conserved hypothetical protein
		C4B63_6g236	cation transporter
		C4B63_70g93	DNA repair and recombination helicase protein PIF2
		C4B63_73g16	conserved hypothetical protein
		C4B63_7g209	Ribonuclease P protein subunit p25-like protein
		C4B63_80g17	ATP binding protein-like protein
		C4B63_81g18	chaperonin HSP60, mitochondrial precursor(fragment)
		C4B63_85g83	Eukaryotic initiation factor 4A-1
		C4B63_97g34	P-type H+-ATPase
		C4B63_97g36	P-type H+-ATPase
		C4B63_98g4	glucose-6-phosphate 1-dehydrogenase

Table S5. List of genes with sustained differential expression through the t_4 vs. t_0 and t_{24} vs. t_0 analysis in hemin and hemoglobin (Hb) conditions.

Table S6

Hemin vs. No heme (t4)			
ID	Annotaton	log2FC	padj
C4B63_18g39	CRAL/TRIO, N-terminal domain/CRAL/TRIO dom	-1,698147969	2,66001E-10
C4B63_27g1020c	RNA-binding protein	-0,939278435	8,07905E-07
C4B63_27g184	integral membrane transport protein	-0,672629407	0,016102027
C4B63_51g80	ferric reductase transmembrane protein	-0,450166135	0,001306312
C4B63_51g81	ferric reductase transmembrane protein	-0,519573589	1,24874E-06
C4B63_51g82	ferric reductase transmembrane protein	-0,461908344	9,80739E-06
C4B63_51g83	ferric reductase transmembrane protein	-0,48197602	2,01094E-05
C4B63_51g85	ferric reductase transmembrane protein	-0,530149195	3,29905E-06

Hemin vs. No heme (t24)			
ID	Annotaton	log2FC	padj
C4B63_18g39	CRAL/TRIO, N-terminal domain/CRAL/TRIO domain co	-1,781020136	2,83772E-29
C4B63_10g454	ABC transporter(fragment)	-1,147001515	0,023416274
C4B63_27g1020c	RNA-binding protein	-1,053472531	1,14104E-05
C4B63_10g455	ABC transporter	-1,02948615	0,001536276
C4B63_462g9	glutamamyl carboxypeptidase(fragment)	-0,910806702	0,023416274
C4B63_51g82	ferric reductase transmembrane protein	-0,822584392	1,46408E-15
C4B63_51g81	ferric reductase transmembrane protein	-0,799944792	5,08189E-14
C4B63_24g988c	conserved hypothetical protein	-0,798755109	0,003092612
C4B63_51g83	ferric reductase transmembrane protein	-0,786642986	1,03938E-10
C4B63_46g80	conserved hypothetical protein	-0,750841729	0,025368929
C4B63_51g85	ferric reductase transmembrane protein	-0,742654566	1,87681E-08
C4B63_51g80	ferric reductase transmembrane protein	-0,731765312	4,71039E-09
C4B63_69g318c	conserved hypothetical protein	-0,729726224	0,001060547
C4B63_32g209	*Tc* HRG	-0,729531427	1,87681E-08
C4B63_27g184	integral membrane transport protein	-0,623790412	0,000179134
C4B63_23g43	conserved hypothetical protein	-0,521299198	0,001035815
C4B63_22g738c	calpain-like cysteine peptidase	-0,457503214	0,03424526
C4B63_42g103	pyridoxal kinase	0,434936738	0,023416274
C4B63_44g117	conserved hypothetical protein	0,443705889	0,001690098
C4B63_7g135	Hydroxymethylglutaryl-CoA lyase, mitochondrial	0,461459445	0,039991998
C4B63_70g116	amino acid permease/transporter	0,508197543	0,001690098
C4B63_47g72	enolase	0,511176423	7,37702E-06

Hb vs. No heme (t4)			
ID	Annotaton	log2FC	padj
C4B63_18g39	CRAL/TRIO, N-terminal domain/CRAL/TRIO dom	-1,43222839	7,81732E-07
C4B63_27g1020c	RNA-binding protein	-1,005231966	1,71295E-07
C4B63_32g209	*Tc* HRG	-0,676872385	0,001798043
C4B63_51g81	ferric reductase transmembrane protein	-0,404398408	0,002429377
C4B63_51g83	ferric reductase transmembrane protein	-0,441116788	0,000799977
C4B63_51g85	ferric reductase transmembrane protein	-0,442097131	0,001384228

Hb vs. No heme (t24)			
ID	Annotaton	log2FC	padj
C4B63_18g39	CRAL/TRIO, N-terminal domain/CRAL/TRIO domain co	-1,009685067	1,41308E-07
C4B63_44g117	conserved hypothetical protein	0,426749137	0,034246414
C4B63_51g81	ferric reductase transmembrane protein	-0,41343673	0,048118011
C4B63_51g82	ferric reductase transmembrane protein	-0,426087127	0,031101978

Table S6. List of DEGs identified in the heme-supplemented conditions vs. no heme condition analysis at t_4 and t_{24}.

Table S7

Model 0

Name	Value
chain_iptm	[List]
chain_pair_iptm	[List]
chain_pair_pae_min	[List]
chain_ptm	[List]
fraction_disordered	0
has_clash	0
iptm	0,85
num_recycles	10
ptm	0,89
ranking_score	0,86

Model 1

Name	Value
chain_iptm	[List]
chain_pair_iptm	[List]
chain_pair_pae_min	[List]
chain_ptm	[List]
fraction_disordered	0
has_clash	0
iptm	0,85
num_recycles	10
ptm	0,89
ranking_score	0,86

Model 2

Name	Value
chain_iptm	[List]
chain_pair_iptm	[List]
chain_pair_pae_min	[List]
chain_ptm	[List]
fraction_disordered	0
has_clash	0
iptm	0,83
num_recycles	10
ptm	0,89
ranking_score	0,84

Model 3

Name	Value
chain_iptm	[List]
chain_pair_iptm	[List]
chain_pair_pae_min	[List]

chain_ptm	[List]
fraction_disordered	0
has_clash	0
iptm	0,79
num_recycles	10
ptm	0,88
ranking_score	0,8

Model 4

Name	Value
chain_iptm	[List]
chain_pair_iptm	[List]
chain_pair_pae_min	[List]
chain_ptm	[List]
fraction_disordered	0
has_clash	0
iptm	0,78
num_recycles	10
ptm	0,88
ranking_score	0,8

Table S7. Confidence scores of the five AlphaFold predicted 3D structures of *Tc*CRAL/TRIO. Tabulated version of the JSON data from Alphafold for ease of viewing. Raw output of confidence scores from AlphaFold for the five predicted 3D models of *Tc*CRAL/TRIO at UNR RDA (https://doi.org/10.57715/UNR/BPKC2V).
